# The Role of Filippi’s Glands in the Silk Moths Cocoon Construction

**DOI:** 10.3390/ijms222413523

**Published:** 2021-12-16

**Authors:** Hana Sehadova, Radka Zavodska, Lenka Rouhova, Michal Zurovec, Ivo Sauman

**Affiliations:** 1 Biology Centre of the Czech Academy of Sciences, Institute of Entomology, 37005 Ceske Budejovice, Czech Republic; sehadova@entu.cas.cz (H.S.); radkaz@pf.jcu.cz (R.Z.); rouhol00@jcu.cz (L.R.); 2 Faculty of Science, University of South Bohemia, 37005 Ceske Budejovice, Czech Republic; 3 Faculty of Education, University of South Bohemia, 37005 Ceske Budejovice, Czech Republic

**Keywords:** Filippi’s glands, Saturniidae, cocoon structure, *Bombyx mori*, silk, proteomic analysis

## Abstract

Filippi’s glands (FGs), formerly also called Lyonet’s glands, are accessory secretory structures of the labial (silk) glands of lepidopteran caterpillars, which were implicated to play an important role in the maturation of the silk material and the construction of the cocoon. In our previous study, we have identified several species of giant silk moths that completely lack the FGs. Interestingly, the absence of FGs in these species correlates with the construction of a loose cocoon architecture. We investigated the functions of FGs by their surgical extirpation in the last instar larvae of the silkworm, *Bombyx mori.* We found that the absence of FGs altered the structure of the resulting cocoon, in which the different layers of silk were separated. In further experiments, we found no effects of the absence of FGs on larval cocoon formation behavior or on changes in cocoon mass or lipid content. Differential proteomic analysis revealed no significant contribution of structural proteins from FGs to silk cocoon material, but we identified several low abundance proteins that may play a role in posttranslational modifications of some silk proteins. Proteomic analysis also revealed a difference in phosphorylation of the N-terminal sequence of fibroin-heavy chain molecule. Thus, FGs appear to affect silk stickiness during spinning by regulating posttranslational modifications. This could also explain the link that exists between the absence of these glands and the formation of loose cocoons in some giant silk moth species.

## 1. Introduction

The Filippi’s glands (FGs) are bilateral accessory glandular structures of lepidopteran silk glands (SGs). They were found in variety of lepidopteran species from ancestral to advanced families [1,2,3,4,5,6,7,8]. Based on their morphology the FGs were divided into two main groups: (1) glands with simple glandular lobes without any apparent gland canals, (2) glands with well-developed leaf-like or tubular lobes with tubular ducts entering the dorsal side of the anterior silk gland (ASG) [1].

It has been suggested that FGs may play several important roles in the process of maturation of silk material in ASGs as well as in the process of silk spinning. One of the proposed functions is the production of cementing substances for the silk fibers [9,10]. However, in several studies, such substances were never detected in FGs [11]. Recent studies of FGs in *B. mori* have suggested that FGs may be involved in regulating pH and transporting small solutes (ions, sugars, and amino acids) to ASGs [12,13]. Additional metabolomic analysis of FGs in *B. mori* revealed active fatty acid biosynthesis [11]. Such FGs products could contribute to the process of silk fiber formation and spinning. The changes in ion concentrations may affect the physical properties of the forming silk fibers (e.g., adhesivity or viscosity), and fatty acids could serve as lubricants during the spinning process. Clustered lipid granules were reported in the developed FGs of the tropical tasar silkworm, *Antheraea mylitta* [3]. Despite these findings, the exact role of FGs in the formation and silk threads and spinning of silk has not been fully elucidated. Considering that the complete removal of FGs in the larvae of the silkworm *B. mori* had no obvious effects on silk spinning behavior and silk quality [14,15], it is reasonable to assume that the role of FGs in cocoon spinning is probably not crucial, but rather complementary. Recent studies on some giant silkworm caterpillars (Saturniidae, superfamily Bombycoidea) have shown that FGs are completely absent throughout larval development in some of these species, although they spin remarkable silk cocoons [16]. It is known that giant silk moths can spin cocoons that consist of multiple layers of silk. Based on their overall morphology, cocoons can be divided into two distinct types: (1) compact cocoons, in which all silk layers are glued together and form a single cocoon shell; (2) loose cocoons in which the silk layers form three distinct compartments: the outer shell, the intermediate silk scaffolding, and the inner shell [17,18,19,20,21].

In this study we investigate a correlation between the presence or absence of FGs, and the type of cocoons constructed. We have previously shown that Saturniid species with well-developed FGs always spin compact cocoons, while species without FGs build loose cocoons [16]. To confirm the function of FGs in cocoon compactness, we surgically removed FGs in the last larval instar of the silkworm *B. mori*. Intact *B. mori* larvae produced compact cocoons. In contrast, larvae in which the FGs were removed produced loose cocoons with a less compact silk layer architecture. Previous speculation about the role of FGs in regulating silk pH in ASG and/or the amount of lipids in the cocoon was not confirmed. However, we detected relatively minor changes in the abundance of several proteins and in the post-translational modifications (phosphorylation) of the fibroin heavy chain (FibH) that could have an impact on regulating the compactness of the cocoon.

## 2. Results

### 2.1. Silk Moth Species without Filippi’s Glands Construct Loose Cocoons

**Figure 1:**
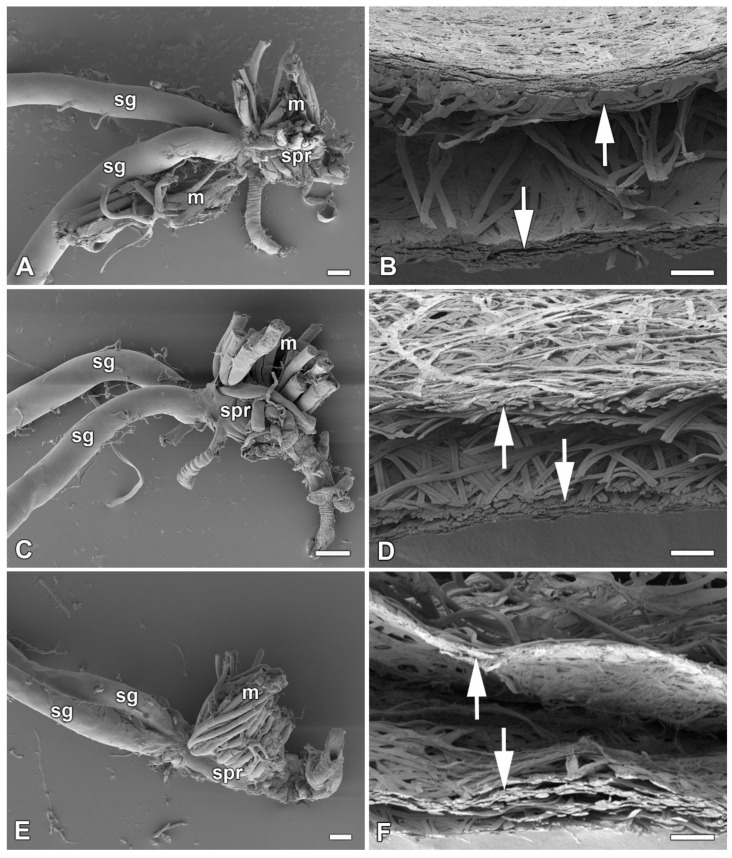
Spinning apparatus of last instar larvae without FGs and cross sections of their loose cocoons. (**A**,**B**) The cecropia silk moth, *Hyalophora cecropia.* (**C**,**D**) The ailanthus silk moth, *Samia cynthia.* (**E**,**F**) The Atlas moth, *Attacus atlas.* Scale bars: (**A**,**C**,**E**) = 100 µm; (**B**,**D**,**F**) = 200 µm. Abbreviations: silk glands (sg), silk press of the spinning apparatus (spr), muscles (m). The arrows depict two distinct layers of the cocoon.

**Figure 2:**
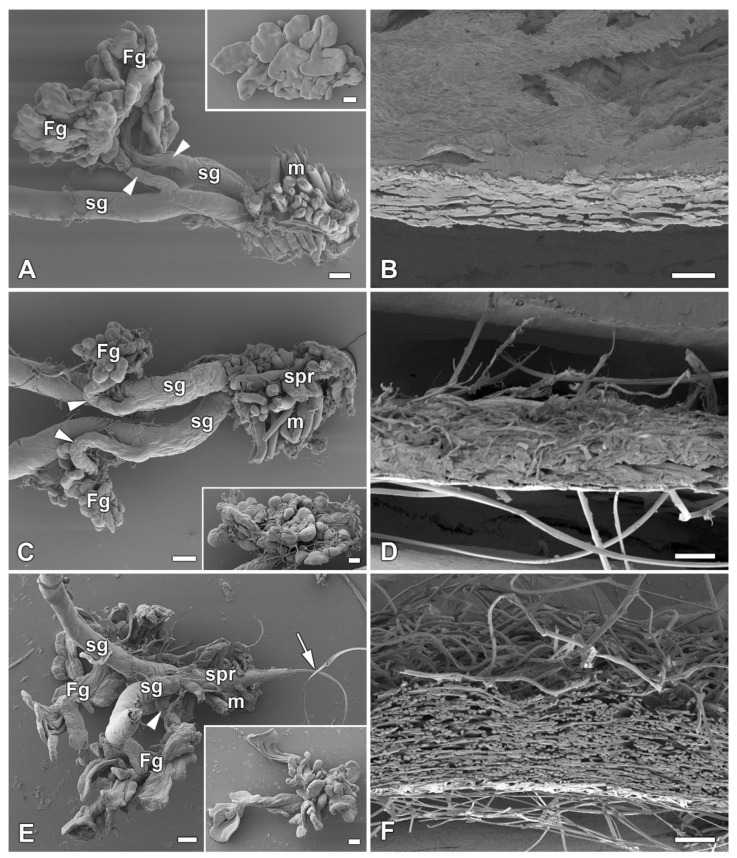
Spinning apparatus of last instar larvae with FGs and cross sections of their compact cocoons. (**A**,**B**) The Polyphemus moth, *Antheraea polyphemus.* (**C**,**D**) The Indian moon moth, *Actias selene.* (**E**,**F**) The commercial silkworm, *Bombyx mori*. Scale bars: (**A**,**C**,**E**) = 100 µm; (**B**,**D**,**F**) = 200 µm. Abbreviations: Filippi’s glands (Fg), silk glands (sg), silk press of the spinning apparatus (spr), muscles (m). The arrowheads indicate the ducts of the Filippi’s glands entering the lumen of the silk glands. The arrow points to the secreted silk fibre.

In our previous study [16], we identified six species of giant silkmoth (Saturniidae) that lack FGs throughout their larval development. Here we were interested whether the presence of FGs had effect on the type of cocoon constructed. We chose three representatives of the Saturniidae that lack FGs (*Hyalophora cecropia, Samia cynthia*, and *Attacus atlas*), two Saturniid species that have well-developed FGs (*Antheraea polyphemus* and *Actias selene*), and the silkworm *B. mori* from the sister family Bombycidae, which has well-developed FGs relative to the size of its spinneret. We found that species without FGs spin loose cocoons in which the silk layers formed outer and inner envelopes with an intermediate silk scaffold (Figure 1), whereas species with well-developed FGs exclusively form compact cocoons with silk in a compact layer (Figure 2). Thus, the presence or absence of FGs was clearly correlated with the type of cocoon spun.

**Figure 3:**
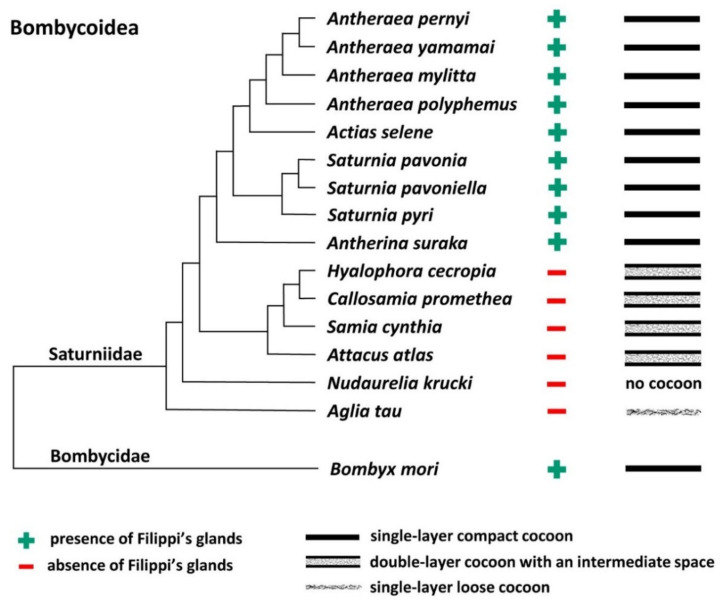
Phylogenetic tree of the superfamily Bombycoidea in relation to the presence/absence of FGs and the type of cocoon. The presence/absence of FGs is based on published data [3,16] and cocoon structure of *A. mylitta*, *A. pernyi*, *A. yamamai*, *S. pavonia*, *S. pyri*, and *C. promethea* was previously studied by Chen et al. [19]. The types of cocoons of the other species in this study are indicated in Figure 1 and Figure 2 and Figure S1 in the Supplementary Materials. The phylogenetic tree was based on Chen et al. [20] and Hamilton et al. [22].

To further support this conclusion, we also compared all published results demonstrating the presence or absence of FGs in giant silkworms [3,16] with their respective cocoon architecture [18,19,21]. For the following species, *Saturnia pavoniella*, *Antherina suraka*, and *Aglia tau*, where the complete absence of FGs has been demonstrated [16] and whose cocoon structure has not yet been described in the literature, we used SEM to show that they form compact cocoons (Supplementary Materials Figure S1). The correlation between the presence of FGs and the type of cocoon architecture is shown in Figure 3. Larvae of Saturniid species with FGs exclusively construct the compact cocoons (*Antheraea pernyi*, *Antheraea yamamai*, *Antheraea mylitta*, *A. polyphemus*, *A. selene*, *S. pavonia*, *S. pavoniella*, *Saturnia pyri*, and *A. suraka*). In contrast, species of silk moths that do not have FGs spin loose cocoons (*H. cecropia*, *C. promethea*, *S. cynthia*, *A. atlas*). The most ancestral Saturniid species examined in this study, *A. tau*, has no FGs and forms a sparse silk cocoon (Supplementary Materials Figure S1C).

### 2.2. Effect of Filippi’s Glands Removal in B. mori Larvae

To study the effect of FGs on cocoon architecture, we chose the silkworm *B. mori*, in which these glands are well developed and defined, and the larvae form compact cocoons. Before spinning begins, the larvae of the last instar empty their gut contents and then enter the wandering stage. After the onset of silk extrusion, we surgically removed the FGs and allowed the larvae to spin cocoons. The efficiency of cocoon construction after surgery was more than 61% (27 of 44 larvae) compared to control animals, where the efficiency was 75% (15/20). Removal of FGs had no obvious effect on larval spinning behavior. After resuming the spinning following the anesthesia and surgery (approximately 3–6 h), the time of cocoon construction was comparable to that of the non-operated control larvae (about 48 h).

**Figure 4:**
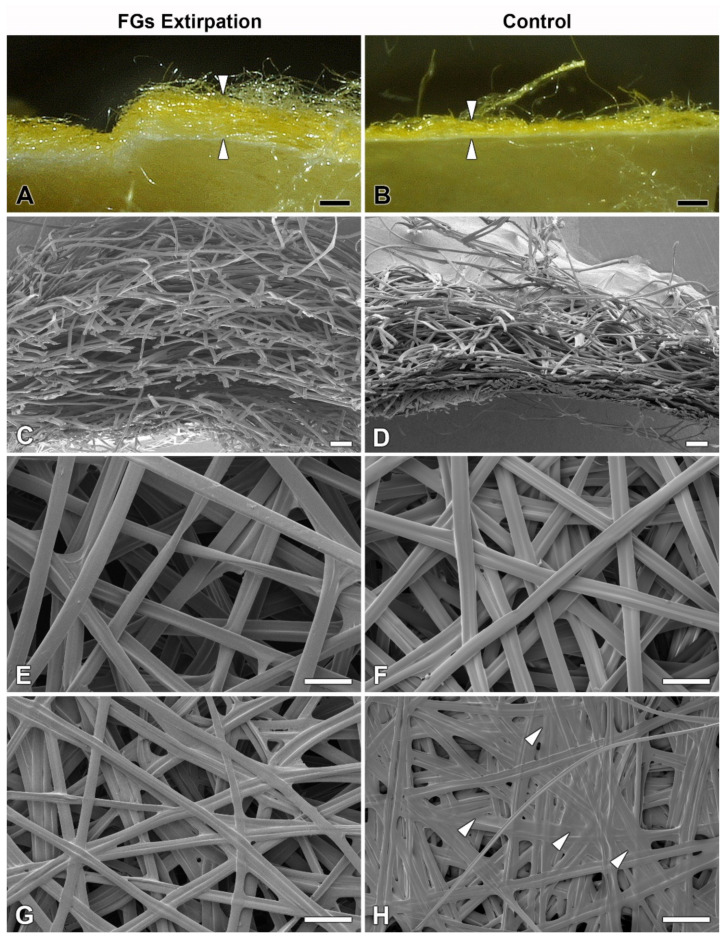
Effect of surgical removal of FGs in silkworm larvae on cocoon structure. (**A**) A macrophotographic image of the cross section of the cocoon wall spun by a *B. mori* larva after complete removal of both FGs. Note the much thicker and looser structure of the cocoon wall (arrowheads). (**B**) Corresponding image from a control animal with FGs intact. The arrowheads indicate the thickness of the normal compact cocoon wall. (**C**) A scanning electron micrograph showing a cross section of the architecture of the loose silk fibers of the cocoon wall of a *B. mori* larva with surgically removed FGs. (**D**) The silk fibers of a control larva with intact FGs are densely arranged and form a much more compact cocoon wall. (**E**,**F**) A microstructure of the cocoon silk fibers from the outside of the cocoon wall shows no visible differences between operated and control animals. (**G**,**H**) The inner sides of the cocoon walls show significant differences in the amount of a “glue-like” material deposited on the silk fibers of the control (arrowheads) compared to the operated larvae. Scale bars: (**A**,**B**) = 1 mm; (**C**,**D**) = 100 µm; (**E**–**H**) = 50 µm.

However, when we compared the cocoon architecture between the operated and control animals, we found a striking difference in the compactness and thickness of the cocoon wall structure. In the cocoons of the operated animals, the silk layers were loosely arranged compared to the very dense structure of the layers in the cocoons of the control animals (Figure 4). These differences were observed at the level of light microscopy (Figure 4A,B). Even more convincing evidence for these structural differences was provided by scanning electron microscopy (SEM) examination (Figure 4C,D). High-resolution SEM images of the outer and inner sides of the cocoon wall revealed striking differences in the arrangement of silk fibers. On both sides, cocoons from animals with extirpated FGs exhibited a much looser arrangement (Figure 4E–H). The most striking differences were observed on the inner side, where the cocoons of operated animals had a significantly lower coverage with sericins than the cocoons of control animals, where sericin covered a considerable part of the surface (Figure 4G,H).

### 2.3. The Filippi’s Glands Have No Effect on pH Regulation or Lipid Synthesis

**Figure 5:**
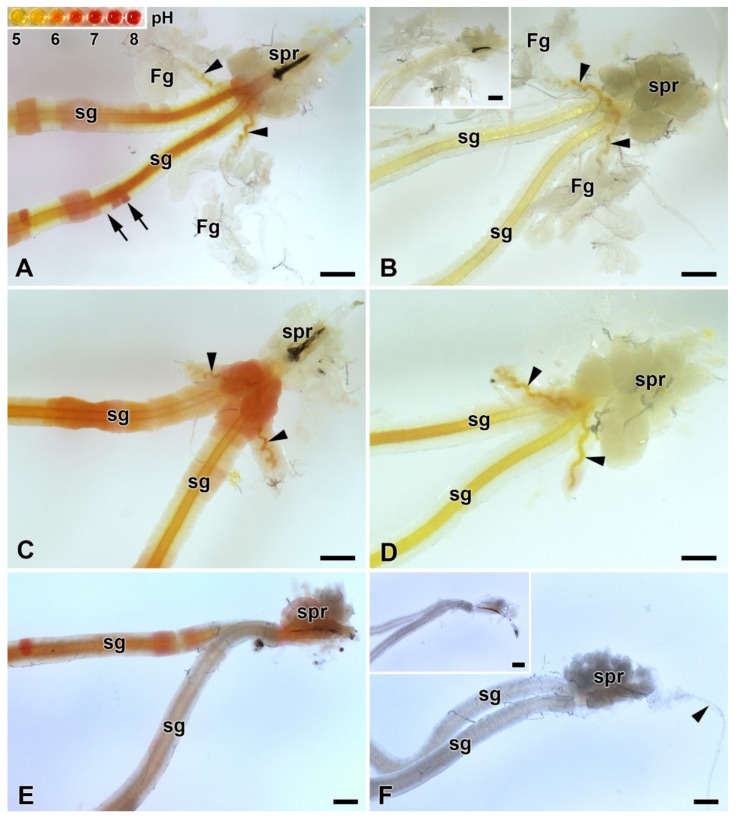
Acidification of the spinning apparatus of *B. mori and* the Eri silk moth (*Samia cynthia*) last instar larvae. (**A**) Brightfield micrograph of the intact spinning apparatus (control) of a “right now non-spinning” *B. mori* larva injected with the pH-sensitive dye (phenol red). The arrowheads show the ducts of the FGs entering the lumen of SGs. Inset: the pH scale of phenol red obtained with pH-calibrated solutions with 0.5 pH resolution (from pH 5 to 8). The arrows indicate significantly different pH values even in adjacent epithelial cells of the silk gland. (**B**) The spinning apparatus of a “right now spinning” *B. mori* larva injected with the phenol red dye. Arrowheads indicate the ducts of the FGs, which open into the lumen of sSGs. Inset: A spinning apparatus of *Bombyx* larva without phenol red injection (negative control for the effect of the phenol red injection). (**C**) Same as in (**A**) but in a larva that has had both FGs surgically removed. The arrowheads show the remains of the FGs ducts after their surgical removal. (**D**) The same as in (**B**) in a larva with extirpated FGs. The arrowheads show the residual ducts of the FGs opening into the lumen of the SGs. (**E**) Brightfield micrograph of the spinning apparatus of a “right now non-spinning” *S. cynthia* larva injected with the pH-sensitive dye phenol red. (**F**) The spinning apparatus of a “right now spinning” *S. cynthia* larva injected with the phenol red dye. Inset: a spinning apparatus of a *Samia* larva without phenol red injection (negative control for the effect of phenol red injection). All scale bars = 200 µm. Abbreviations: Filippi’s glands (Fg), silk (labial) glands (sg), silk press of the spinning apparatus (spr).

Because the FGs can be involved in the transport of ions [13], we investigated their possible role in regulating the pH of silk in ASGs, during spinning. To test this possibility, pH was measured in vivo using the indicator dye phenol red. No differences in silk pH or in the epithelial cells of the SGs were observed between the FGs extirpated and the control larvae. Significant differences in the pH of the different parts of the spinning apparatus were found only between the *B. mori* larvae that resumed silk extrusion after application of the phenol dye and those that did not. In both, operated and unoperated larvae, that did not resume silk spinning, the anterior part of the SGs contained clusters of cells with higher pH (approximately between 6.5 and 7.5) and silk-like material in the lumen of the SGs and FG ducts with pH values around 6.5 (Figure 5A,C). In contrast, larvae (both, operated and control) that had resumed silk extrusion had a much more acidic pH of the entire spinning apparatus including the silk material (pH below 5.0; Figure 5B,D). 

Similar changes in pH in ASGs between spinning and non-spinning larvae were also observed in a silk moth species lacking FGs (*S. cynthia*) when, after resumption of spinning, the pH values were lower than 5 and in those that did not start re-spinning after dye injection, the pH in some epithelial cells reached pH about 7.0 (Figure 5E,F).

**Figure 6:**
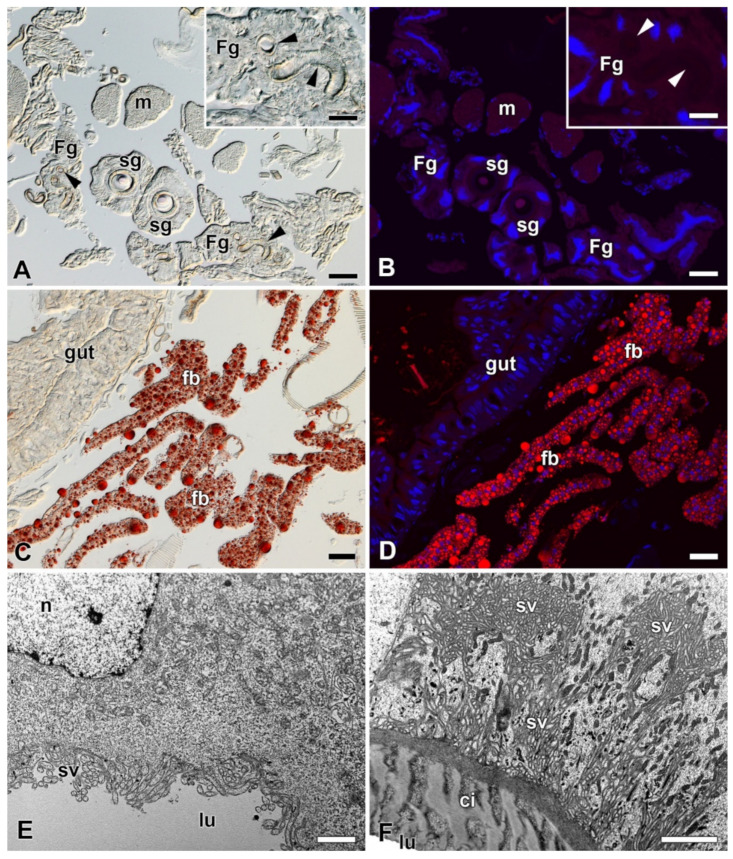
Detection of fat in the FGs and SGs of *B. mori*. (**A**) Brightfield micrograph of a cross section of FGs and SGs stained with Oil Red O dye for the presence of fat granules. No positive signal is visible. Arrowheads show the ducts of the FGs. Inset: A higher magnification of the FG. Arrowheads show the duct of the FG. (**B**) The same section as in (**A**) shown under a fluorescence microscope. No fat granules were detected with Oil Red O staining. The cell nuclei (blue) were counterstained with DAPI. (**C**) A section through an anterior part of the larval abdomen stained with Oil Red O dye showing the fat granules in the perivisceral fat body lobes. (**D**) The same as in (**C**) under a fluorescent microscope. Fat granules stained with Oil Red O are clearly visible in the fat body lobes. Blue, cell nuclei stained with DAPI. (**E**) A transmission electron micrograph of a secretory cell from the FGs. No fat granules are found in the cytoplasm of the cell. (**F**) A transmission electron micrograph of an epithelial cell from the SG. No lipid granules are found in the cell cytoplasm. Scale bars: (**A**–**D**) = 25 µm; insets in (**A**,**B**) = 25 µm; (**E**,**F**) = 1 µm. Abbreviations: Cuticular intima (ci), fat body (fb), Filippi’s glands (Fg), midgut epithelium (gut), lumen of FGs and SG duct (lu), muscles of silk press (m), nucleus (n), silk glands (sg), secretory vesicles (sv).

Considering the published data demonstrating the presence of lipids in the FGs of *A. mylitta* [3] and active fatty acid biosynthesis in the FGs of *B. mori* [11], we examined the presence of lipids and fatty acids in FGs of *B. mori, A. selene*, and *A. polyphemus.* The presence of lipids in the FGs was not detected by Oil Red O staining in any of the species tested (Figure 6A,B; Supplementary Materials Figure S2A,B). Examination of the ultrastructure of FGs in *B. mori* also did not confirm the presence of lipid droplets or other vesicles with lubricating substances (Figure 6E,F). Since the presence of lipid droplets has been demonstrated in ASGs of some lepidopteran species [23,24], we also thoroughly analyzed this part of SGs. However, the presence of lipid components was not detected in the ASGs either, both in the above-mentioned species with FGs (Figure 6A,B,E,F; Supplementary Materials Figure S2A,B), and in species without FGs: *H. cecropia* and *S. cynthia* (Supplementary Materials Figure S2C–F).

### 2.4. Proteomic Analysis of Silk from FGs Ablated and Non-Ablated Larvae

To determine whether any specific proteins are added to the silk material from FGs, we performed a differential proteomic analysis of *B. mori* cocoons. We detected 2535 tryptic peptides within the reference in the UniProt database and identified more than 240 proteins (Supplementary Materials Table S2). Protein abundance was calculated using MaxQuant/Andromeda software (MPI of Biochemistry, Martinsried, Germany). Annotation of the identified proteins revealed that they included all previously known highly abundant structural silk proteins (fibroins, sericins, protease inhibitors or seroins) as well as with a number of less abundant enzymes and cellular proteins deposited in the silk by apocrine secretion. Comparison of the amounts of individual silk proteins from the cocoons of control and FGs ablated larvae revealed relatively minor differences (Supplementary Materials Table S2). Only two proteins showed statistically significant decreases in the cocoons of ablated larvae using Perseus analysis software, the enzyme apyrase (H9JTT3) and a small molecule of a Kazal-type trypsin protease inhibitor (P51902). While apyrase was 13-fold lower, the trypsin inhibitor appeared to be completely absent in the cocoons of larvae with ablated FGs. We concluded that trypsin inhibitor may be involved in the regulation of proteolytic processing required for changes in adhesion strength or silk compactness.

### 2.5. Comparison of Our Proteomic Results with Previously Published Expression Data from a Sample of FG-Enriched Transcripts

**Figure 7:**
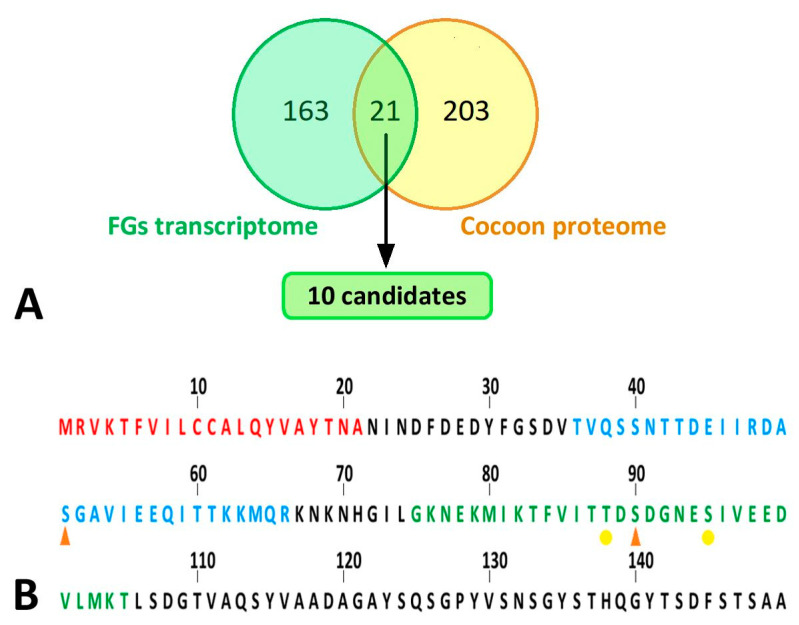
Bioinformatic analyses of silk proteins. (**A**) Comparison of results of proteomic analysis with published transcriptomic data. Yellow circle: all peptides detected by peptide mass fingerprinting; green circle: differentially expressed genes in the FG-enriched sample of wandering larvae spinnerets as published in Wang et al. [13]. The cut set contained a total of 21 proteins (18 of which were upregulated, 3 downregulated in FG-enriched transcriptome). This list of proteins was further narrowed down to 10 candidates (highlighted in green), which are listed in Table 1. (**B**) N-terminal part of *B. mori* FibH. Features indicated by letter colors: red—signal sequence; blue—peptide with significantly lower phosphorylation in larvae with ablated FGs; green—sequence not detected in FG-ablated larvae but containing phosphorylation in control larvae. Phosphorylation sites: orange arrowhead—major; yellow dot—minor.

Because the FGs are relatively small compared to the bulky SGs, candidate proteins from the FGs were expected to have rather low abundance in silk. To identify such candidate proteins with low abundance in the FGs, we compared our proteomic results with previously published expression data from a sample of FG-enriched transcripts from wandering stage larvae [13]. We found a total of 21 common proteins, as shown in the Venn diagram (Figure 7A). Only the proteins also included in the study by Wang et al. [11,13] were considered for further analysis (18 in total). Student’s t-test revealed no protein that was significantly decreased by ablation of FGs. However, the statistics were affected by missing values in the samples of treated larvae. This was true for ten proteins detected in no more than one cocoon of FG-ablated larvae. These ten proteins were added to the candidates (Table 1). Based on the SilkDB 3.0 platform [25,26], eight of the ten candidate proteins (Table 1) are predominantly expressed in SGs (including FGs); the remaining two proteins are less specific and mainly originate from the fat body. Not all candidate proteins also contained a signal peptide for secretion (Table 1). A total of 3 candidate proteins remained, including two enzymes and one protein with unknown function.

### 2.6. Identification and Quantification of Phosphorylation of Silk Proteins

Because two of the three secretory proteins described in the previous paragraph as candidates for differential presence of silks from FG ablated and control larvae were involved in the transfer or removal of phosphate groups, we compared the phosphorylation of the silk proteins using a mass spectrometric approach. The significance of differences was assessed in Perseus using the false discovery rate (Supplementary Materials Table S3, (q values)). Quantitative comparison of silk proteins between the control and ablated larval cocoons revealed a single site of significantly higher phosphorylation in the control group. This was a phospho-serine (S51-ph) within the sequence TVQSSNTTDEIIRDA**S**GAVIEEQITTKKMQR originating from the region near the N-terminus of the FibH protein (Uniprot code P05790) (Figure 7B). Interestingly, another phospho-serine (S90-ph) GKNEKMIKTFVITTD**S**DGNESIVEEDVLMKT of FibH (Figure 7B) located near the first sequence also showed a similar trend. However, it could not be accurately quantified because no signal was detected in cocoons produced by ablated larvae. Overall, the N-terminus of FibH appeared to be phosphorylated at serine 51 in t cocoons of control larvae, and this phosphorylation was absent in cocoons from FG-ablated larvae.

## 3. Discussion

The main objective of this study was to investigate the effect of FGs on the cocoon structure of silkworm larvae. The adhesion strength of the different layers of the cocoon changed after removal of the FGs. The control larvae spun very compact cocoon walls, whereas the operated larvae had loose cocoons in which the individual silk layers were only very loosely connected. This finding suggests that the FGs affect the physical properties of the extruded silk fibers. We successively tested the possible additions of FGs in the form of lipids, and structural proteins. Finally, using proteomics methods, we found significant differences in the presence of some enzymes and in the phosphorylation of serine residues at the N-terminus of FibH in control cocoons compared to cocoons from caterpillars that had their glands removed.

Previous studies performing comparative transcriptome analysis have shown that FGs contain many different transcripts of genes encoding ion channels and inorganic and organic ion carriers. Previous results therefore suggest that FGs may regulate ion flux and thus influence pH changes in forming silk fibers within ASGs. It was hypothesized that physical properties such as viscosity and adhesion of silk fibers may be affected by pH changes [13,28,29,30]. However, our experiments with the pH-indicating phenol red dye contradict this hypothesis, as we did not observe significant changes in pH lin the silk material within ASGs between individuals with extirpated FGs and intact control individuals. Thus, either the FGs have no effect on the pH of the silk material in the ASGs or there are only minor changes that are below the resolution limits of the phenol red method used. However, we found that the pH of some epithelial cell clusters of the SGs changed significantly and thus the SGs themselves can regulate the pH of the silk material in the ASGs.

To verify the previously suggested synthesis and presence of lipids in FGs, we used Oil Red O staining in *B. mori* and two representatives of the giant silk moths that also possess FGs (*A. polyphemus* and *A. selene*). Using this detection method we were unable to detect fat granules in any of the species examined. An examination of the ultrastructure of FGs in *B. mori* also confirmed the absence of fat or other lipid vesicles in FGs, consistent with previously published data on the ultrastructure of FGs in *B. mori* [2,31]. Lipid granules were also not detected in the ultrastructure of FGs from *D. saccharalis* [23]. Thus, the presence of lipid granules in FGs of *A. mylitta* [3] is not typical for all members of the family Saturniidae and could be an exception. However, *Diatraea saccharalis*, has been shown to synthesize and secrete lipids in ASG epithelial cells [23]. Therefore, we subjected the ASGs in both *A. polyphemus* and *A. selene* and in *H. cecropia* and *S. cynthia*, which lack FGs, to a thorough analysis for the presence of lipid. Fat granules were not detected in the epithelial cells of ASG in any of these species. Therefore, it is unlikely that lipid secretion from FGs is responsible for the observed changes in the adhesive strength of the cocoon of *B. mori*.

Thus, the secretions of FGs, which affect the physical properties of extruded silk fibers, do not appear to contain lipids or alter pH. There is a possibility that the secretion of FGs increases or prolongs the adhesiveness of the sericin proteins so that they attach to the individual fibroin fibers, allowing tight cross-linking of the cocoon layers. Ions transported from FGs to ASG might somehow be involved in regulating the conformational change and mechanical properties of silk fibers, although the pH of the silk material does not change dramatically. A comparison of the amounts of individual cocoon proteins between larvae without FGs and control larvae revealed that several proteins were much less abundant or completely absent in the silk of larvae without FGs. The proteins detected include a significant amount of enzymes and cellular proteins secreted into the silk by an apocrine secretion process similar to that found in the salivary glands of *D. melanogaster* [32]. Although our proteomic analysis of silkworm cocoons may lack some low abundance protein components, overall we found similar protein abundance to previous studies [33,34]. In the cocoons of ablated larvae (line 176, Supplementary Materials Table S1), we detected a significantly lower amount of the enzyme apyrase (H9JTT3). Apyrase catalyzes the hydrolysis of ATP to AMP and inorganic phosphate. We also observed the complete absence of a small Kazal-type trypsin inhibitor molecule (P51902) in the cocoons of ablated larvae (line 217, Supplementary Materials Table S1). It seems likely that both apyrase and Kazal-type trypsin protease inhibitor are involved in antimicrobial activity and protection of the cocoon [35,36]. However, Kazal-type protease inhibitors have also been shown to have several other functions in invertebrates, including preventing premature activation of zymogens, preventing coagulation of blood meals in blood-feeding insects, or stimulating cell growth [37]. It will be interesting to determine whether it could be involved in the regulation of sericin adhesion, for example by blocking proteolytic processing of sericin protein.

Another possibility for the regulation of adhesiveness/silk compactness could be at the level of protein phosphorylation. We found a significantly higher level of phosphorylated serine 51 within the TVQSSNTTDEIIRDA**S**GAVIEEQITTKKMQR sequence of FibH. This phosphorylation site is located near the N-terminus, which is relatively conserved among species and relatively accessible to the kinase. Interestingly, we also observed a trend toward phosphorylation of serine 90 near the first site on Fib-H (Table S3). The N-terminal amino acids of FibH are highly hydrophilic and are in contact with the surrounding aqueous solution.

According to the model of silk processing [38], the water here serves as a lubricant to prevent premature crystallization of the silk. The kinase could most likely reach the fibroin core during “physical shearing” at the spinneret. We assume that the phosphorylation of FibH we observed is only the tip of an iceberg. The method used for the analysis has its limitations for the detection of phospho-serine in sericin-specific peptides. This could be due to the extremely high proportion of serine residues and the repetitive nature of the sequences. We suggest that sericins are good candidates for phosphorylation by FG secretion, especially at the surface of the produced filament. We hypothesize that only a minority of the resulting sericin-specific peptides become phosphorylated, but their surface localization may be responsible for the observed change in adhesiveness. More data are needed to confirm the involvement of phosphorylation in regulating adhesion/compaction of silk fibers in the cocoon. For example, phosphorylated serines could contribute to silk fiber self-adhesion via Ca^2+^ crossbridging, as suggested by Stuart and Wang 2010 for caddisfly fibroin [39].

Our current results may explain the correlation between the absence of FGs in giant silk moths (e.g., *H. cecropia*, *C. promethaea*, *S. cynthia*, *A. atlas*) and the formation of loose cocoons. It is possible that most species originally formed compact cocoons and possessed functional FGs, but during evolution FGs lost their importance in cocoon formation, which may have led to their gradual decline and to the evolution of cocoon architecture characterized by loose silk layers.

Overall, FGs seem to influence the stickiness of the silk. *B. mori* larvae in which the FGs have been removed do not form compact cocoons, but the individual cocoon forming layers are loose. The mechanism of this change is unclear, but our data suggest that FGs provide some enzymes that can cause posttranslational modifications of silk proteins, including specific phosphorylation of serine at the N-terminus of the FibH molecule and possible regulation of proteolytic cleavage of unknown silk components. Such a role of FGs could also explain the observed association between the absence of these glands and the formation of loose cocoons in some other silkworm species.

## 4. Materials and Methods

### 4.1. Animals

Larvae of the silkworm, *Bombyx mori* strain N4, were obtained from our laboratory colony and reared on the standard artificial diet as described elsewhere [40]. Eggs of the wild silk moth species used in this study were purchased from Worldwide Butterflies Ltd. (Dorset, UK, www.wwb.co.uk, accessed on 15 November 2021). After hatching, the larvae were reared in a standard insect rearing facility and fed on freshly cut branches of their respective food plants: Indian moon moth, *Actias selene*—weeping willow (*Salix babylonica*); tau emperor moth, *Aglia tau*—small-leaved lime (*Tilia cordata*); Polyphemus moth, *Antheraea polyphemus*—common oak (*Quercus robur*); Suraka silk moth, *Antherina suraka*—Korean privet (*Ligustrum ovalifolium*); Atlas moth, *Attacus atlas*—tree of heaven (*Ailanthus altissima*) or cherry laurel (*Prunus laurocerasus*); cecropia moth, *Hyalophora cecropia*—wild cherry (*Prunus avium*); African silk moth, *Nudaurelia krucki*—cherry laurel (*Prunus laurocerasus*); ailanthus silk moth, *Samia cynthia*—tree of heaven (*Ailanthus altissima*); Ligurian emperor moth, *Saturnia pavoniella*—goat willow (*Salix caprea*).

### 4.2. Scanning and Transmission Electron Microscopy

For scanning electron microscopy (SEM), tissues dissected in the phosphate buffer saline (PBS) were fixed in 2.5% glutaraldehyde in PBS for at least 4 h at room temperature (RT) or overnight at 4 °C. The samples were washed in washing solution containing PBS supplemented with 4% glucose (three times for 15 min at RT) and subsequently treated with 1:1 mixture of PBS and 4% solution of osmium tetroxide (2 h at RT). After application of the washing solution (three times for 15 min at RT) the tissues were dehydrated in acetone series (30%, 50%, 70%, 80%, 90%, 95%, and 100% for 15 min each), and critical point dried. Dried samples were glued to aluminum holders, sputter-coated with gold, and observed under Jeol JSM-7401F scanning electron microscope (JEOL Ltd., Tokyo, Japan).

For transmission electron microscopy (TEM), dehydrated specimens were embedded in the Epon resin. Semithin sections were cut with a glass knife on a water surface and placed on a microscope slide containing 10% acetone. Dried sections were stained with 1% toluidine blue. Ultrathin sections were cut with a diamond knife on a water surface. After stretching by chloroform, the sections were placed on copper grids and contrasted with uranyl acetate and lead citrate and coated with carbon. The samples were imaged under Joel 1010 transmission electron microscope.

### 4.3. Extirpation of Filippi’s Glands

The FGs were removed from the last instar larvae, which had begun silk spinning. The operation was performed under deep carbon dioxide anesthesia. To achieve continuous anesthesia the caterpillar was placed into a small plastic bag with a carbon dioxide supply, and only a small window was made in the bag at the site of surgery. The cuticle under the spinneret was first sterilized by wetting with 95% ethanol and then the FGs were removed from both SGs with fine microsurgical forceps through miniature incisions in the cuticle. The FGs are located ventrolateraly from the spinneret in the close vicinity to the head capsule cuticle. The wounds were sealed with a thin layer of a silicone grease after surgery to prevent hemolymph leakage. The treated animals were placed in rearing containers to spin cocoons. The efficiency of the surgery was verified by dissections of several randomly selected operated larvae. In all cases FGs were successfully and completely removed.

### 4.4. Injection of pH-Sensitive Phenol Red Dye

Both control and FGs-ablated larvae that began spinning cocoons were anesthetized with carbon dioxide and injected into the abdominal hemocoel with 80 µL of 5 mM phenol red in 5 mM Mops (3-morpholinopropanesulfonic acid)-Tris buffer (pH 7.0). Larvae were then placed in plastic containers where about half of them resumed cocoon spinning once they recovered from anesthesia. Larvae were sacrificed 3–4 h after dye injection and dissected ASGs of 5 animals from each group were examined under a stereomicroscope (Olympus SZX16, Olympus Corporation, Tokyo, Japan). Final micrographs were reconstructed by stitching together multiple Z-stack images.

### 4.5. Staining of Lipids

A portion of the head containing spinning apparatus including the spinneret, ASGs, and FGs was dissected in Ringer’s solution and fixed overnight at 4 °C in 3.7% formaldehyde in phosphate buffered saline (PBS). Samples were rinsed three times for 10 min in PBS and then macerated in 25% sucrose (overnight at 4 °C) followed by Tissue-Tek (Sakura; overnight at 4°C). Then, 5 µm cryosections were cut, rinsed with distilled water followed by 60 % isopropanol, and stained with Oil Red O (Sigma-Aldrich, Inc., St. Louis, MO, USA) solution (0.5% in isopropanol) for 10 min at room temperature. After rinsing with 60% isopropanol and PBS, samples were mounted in Fluoroshield medium with DAPI DNA stain (Sigma-Aldrich). Stained tissues were visualized under a brightfield (Olympus BX51), and confocal (Olympus FV3000) microscopes.

### 4.6. Cocoon Protein Digestion, nLC-MS/MS Analysis, and Database Search

Protein samples were prepared for mass spectrometry as previously described [41]. *B. mori* silk cocoon samples (from FGs ablated and control non-ablated larvae) were analyzed in quadruplicates. Silk samples (5 mg) were dissolved in 8 M urea, trypsinized and acidified with trifluoroacetic acid (to 1% final concentration). Peptides were desalted and analyzed by nanoscale liquid chromatography coupled to tandem mass spectrometry (nLC-MS/MS). Peptides were analyzed and quantified using MaxQuant algorithms (MaxQuant, version 1.5.3.8; [42]). The search engine Andromeda [43] was used to match peptide MS/MS spectra with a UniProt database (http://www.uniprot.org). For analysis of phosphorylation sites STY was set as a variable modification for MaxQuant search. Statistical analysis was performed using Perseus 1.5.2.4 software [44]. We also compared a protein database from our proteomic data and compared it with the gene products previously discovered by Wang et al. [13] to be differentially expressed between spinneret samples with or without FGs in *B. mori* wandering larvae after gut purging. The BLASTp algorithm was used to identify shared proteins between these two databases. The resulting candidates were screened for the presence of the signal peptide for secretion SignalP-5.0 [27], and their tissue specificity was assessed using SilkDB 3.0 [26].

## Figures and Tables

**Table 1:** A definitive list of candidate proteins expressed in both the FG-enriched transcriptome [13] and proteins underrepresented in the silk of FG-depleted larvae. Data on their expression specificity were found in SilkDB 3.0 [25,26] and the presence of a signal peptide for secretion was predicted using SignalP 5.0 [27].

Uniprot ID	SilkDB 3.0 ID	Expressed in SG/FG	Annotation	Signal Peptide
H9IWH6	BGIBMGA001608	Yes	Alpha-1,6-mannosyl-glycoprotein 2-beta-N-Acetylglucosaminyltransferase	No
H9J8H0	BGIBMGA005812	Yes	Arginine kinase	No
H9J9P6	BGIBMGA006239	No	Uncharacterized protein	Yes
H9JBW7	BGIBMGA007012	Yes	Extracellular serine/threonine protein kinase	No
H9JET3	BGIBMGA008030	Yes	Allantoate amidinohydrolase	No
H9JI83	BGIBMGA009232	No	Aldose 1-epimerase OS	No
H9JL76	BGIBMGA010277	Yes	Venom acid phosphatase Acph-1-like	Yes
H9JLC2	BGIBMGA010323	Yes	Uncharacterized protein OS	Yes
H9JPD2	BGIBMGA011386	Yes	Uncharacterized protein OS	No
H9JQ96	BGIBMGA011702	Yes	EN protein binding/engrailed nuclear Homeoprotein-regulated protein	No

## Data Availability

The data presented in this study are available on request from the corresponding author.

## Supplementary Materials

The following are available online at https://www.mdpi.com/article/10.3390/ijms222413523/s1.
