# Supplementary material for: The Role of Filippi’s Glands in the Silk Moths Cocoon Construction
Source: Int J Mol Sci. 2021 Dec 16;22(24):13523. doi: 10.3390/ijms222413523 (PMC8708004; doi:10.3390/ijms222413523)
Supplement: Supplementary file 1 [file ijms-22-13523-s001.zip › ijms-1473911 - Supplementary Figure.pdf]

Supplementary Figure

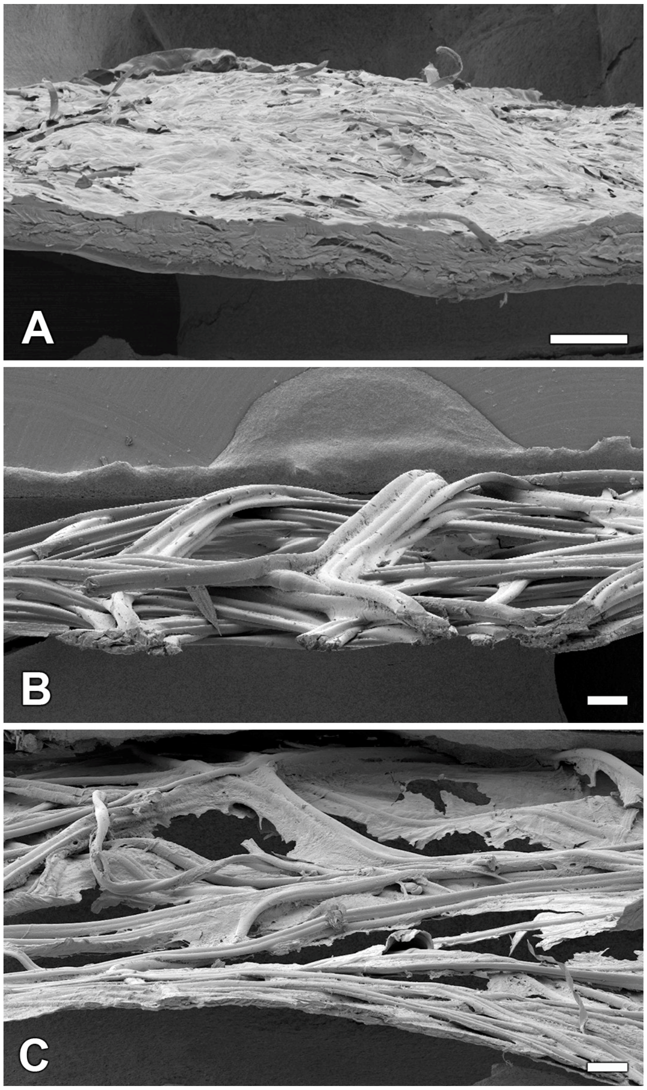

Figure S1. The cross sections of compact cocoons of *Saturnia pavoniella* (**A**), *Antherina suraka* (**B**), and *Aglia tau* (C). Scale bars = 100 μm.

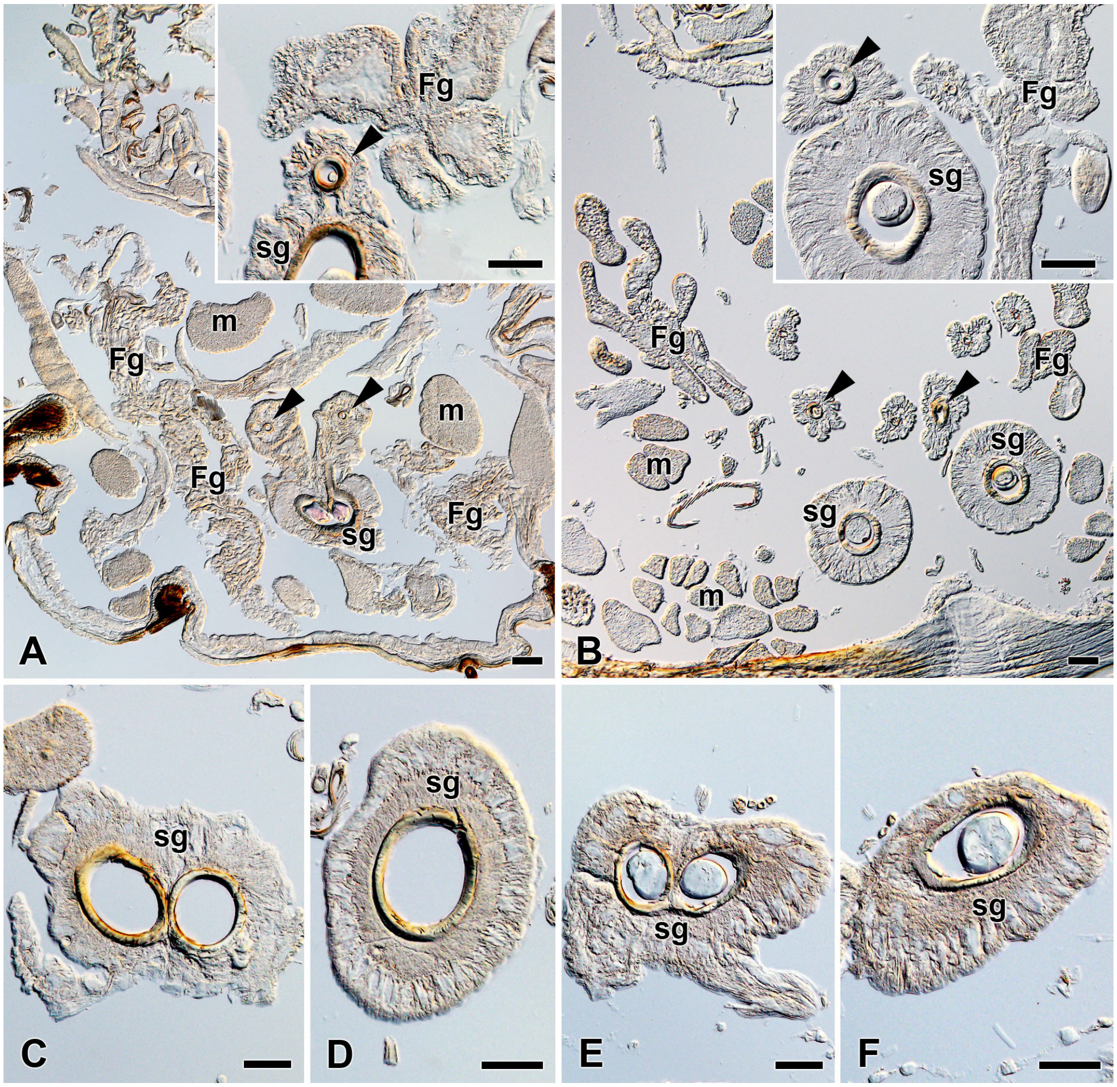

Figure S2. Detection of fat in the Filippi's and silk glands of saturniids last instar larvae. (**A**) Brightfield micrograph of a cross section of Filippi's and silk glands of *Actias selene* stained with Oil Red O dye for the presence of fat granules. No positive signal is visible. Inset: a higher magnification of the Filippi's gland and part of the silk gland. The arrows show the excretory ducts of the Filippi's glands. (**B**) The same as in (**A**), but in the larva of *Antheraea polyphemus*. Arrows show the ducts of the Filippi's glands. (**C**) and (**D**) Transverse sections through the silk glands in the larva of *Cecropia*. No fat staining was detected. (**E**) and (**F**) Transverse sections through the silk glands in the *Samia cynthia* larva. No positive Oil Red O signal was detected. Scale bars = 50 μm. Abbreviations: Filippi's glands (Fg), muscles (m), silk glands (sg).
