# Supplementary material for: The Role of Filippi’s Glands in the Silk Moths Cocoon Construction
Source: Int J Mol Sci. 2021 Dec 16;22(24):13523. doi: 10.3390/ijms222413523 (PMC8708004; doi:10.3390/ijms222413523)
Supplement: Supplementary file 1 [file ijms-22-13523-s001.zip › Table S1.pdf]

Table S1. Comparison of proteins from cocoons spun by control larvae or larvae with ablated FG (experiment was performed in quadruplicate). Quantification (LFQ) Intensities 1-4 and 5-8 show sample data in binary logarithms from ablated larval cocoons and controls, respectively. Detected proteins were annotated using UniProt and Silkdb3.0 databases; quantification was performed using a label-free algorithm and MaxQuant software; statistical analysis was performed using Perseus software (including a one-sample T-test). Mean- and mean+ denote values for ablated and control cocoons, respectively. “NaN” (Non-Assigned Number) values correspond to expression values that were originally zero when proteins were below the detection limit. Quality control (QC) parameters include the number of peptides in the detected protein.

	Protein identifiers		Quantification and statistics											QC Parametrs of identification											Samples data binary logarithm							
	Protein Ids	Fasta headers	Student's T-test Difference FG-_FG+	Fold change	"-Log Student's T-test p-value FG-_FG+"	Mean -	Median FG-	% of valid values	Number of valid values FG-	Mean +	Mediane FG+	% of valid values +	Number of valid values zluta+	Peptides	Razor + unique peptides	Unique peptides	Sequence coverage [%]	Unique + razor sequence coverage [%]	Unique sequence coverage [%]	Mol. weight [kDa]	Q-value	Score	Intensity	MS/MS count	Intensity FG- (1)	Intensity FG- (2)	Intensity FG- (3)	Intensity FG- (4)	Intensity FG+ (1)	Intensity FG+ (2)	Intensity FG+ (3)	Intensity FG+ (4)
1	B9X1S5;H9JJE6;H9JYG5	Histone H4 OS;Histone H4 OS;Histone H4 OS	0.839476108551025	1.79	0.788916663027456	23.88	23.77	100.00	4.00	23.04	22.71	100.00	4.00	6	6	6	48.5	48.5	48.5	11.381	0	41.258	354820000	73	23.27	23.49	24.71	24.06	22.47	24.28	22.77	22.65
2	C0H6H7	Putative cuticle protein OS	NaN	NaN	0	NaN	NaN	0.00	0.00	21.17	21.39	75.00	3.00	1	1	1	14.8	14.8	14.8	14.647	0	8.4524	20277000	11	NaN	NaN	NaN	NaN	NaN	20.71	21.39	21.39
3	C0H6R0;C0H6R2	Putative cuticle protein OS	NaN	NaN	0	NaN	NaN	0.00	0.00	19.93	19.93	50.00	2.00	4	4	4	27.5	27.5	27.5	31.754	0	15.381	6959700	4	NaN	NaN	NaN	NaN	NaN	NaN	19.68	20.19
4	C7EPE2	Ecdysone oxidase OS	NaN	NaN	0	NaN	NaN	0.00	0.00	18.61	18.61	50.00	2.00	2	2	2	4.8	4.8	4.8	61.805	0	4.8555	6047800	6	NaN	NaN	NaN	NaN	NaN	NaN	20.18	17.04
5	E5EVW2	30K protein 2 OS	-1.23119735717773	-2.35	0.208573194409128	21.67	21.71	100.00	4.00	22.90	24.16	100.00	4.00	12	9	6	44.1	33.6	23.4	29.391	0	54.71	534190000	70	19.90	23.36	20.74	22.68	16.76	22.70	26.52	25.62
6	E5EVW3	30kDa protein OS	-1.44965744018555	-2.73	1.23064728491467	19.80	19.96	75.00	3.00	21.24	21.12	75.00	3.00	12	12	12	71	71	71	27.823	0	31.965	94371000	41	NaN	19.96	19.22	20.21	NaN	21.12	20.50	22.11
7	E5EVW5	Inorganic pyrophosphatase OS	NaN	NaN	0	NaN	NaN	0.00	0.00	18.42	18.42	50.00	2.00	2	2	2	13.9	13.9	13.9	32.24	0	5.4642	2034700	3	NaN	NaN	NaN	NaN	NaN	NaN	19.06	17.78
8	F8UN44	Heat shock protein 70-3 OS	-0.355382442474365	-1.28	0.241024710856768	22.42	22.40	100.00	4.00	22.77	22.42	100.00	4.00	22	22	19	44.1	44.1	38.2	72.745	0	144.89	264140000	96	22.42	22.66	22.37	22.21	21.77	22.29	22.56	24.48
9	H9B457	30K protein 24 OS	-2.59820175170898	-6.06	0	NaN	NaN	25.00	1.00	20.46	20.46	50.00	2.00	4	4	4	19.7	19.7	19.7	28.495	0	36.914	11549000	10	NaN	17.86	NaN	NaN	NaN	NaN	20.78	20.14
10	H9IRZ3	Uncharacterized protein OS	0.97142219543457	1.96	0.736675292371702	29.89	29.97	100.00	4.00	28.92	29.27	100.00	4.00	16	16	16	67.4	67.4	67.4	25.909	0	323.31	67867000000	521	29.75	30.26	29.37	30.18	27.18	29.02	29.96	29.52
11	H9IS45	neutral alpha-glucosidase AB-like isoform X1	-2.24265638987223	-4.73	1.10824827456397	21.37	21.77	100.00	4.00	23.62	23.62	75.00	3.00	32	32	32	48.6	48.6	48.6	96.288	0	250.93	349540000	151	19.17	22.01	21.53	22.78	NaN	22.74	23.62	24.49
12	H9ISH9	Calcium-binding EGF domain	NaN	NaN	0	NaN	NaN	0.00	0.00	NaN	NaN	25.00	1.00	4	4	4	14.8	14.8	14.8	48.278	0	17.837	21249000	12	NaN	NaN	NaN	NaN	NaN	NaN	17.41	NaN
13	H9ISI0	fibrillin-1-like	NaN	NaN	NaN	NaN	NaN	0.00	0.00	NaN	NaN	0.00	0.00	2	2	2	5.5	5.5	5.5	60.007	0	17.075	4774700	7	NaN	NaN	NaN	NaN	NaN	NaN	NaN	NaN
14	H9ISI2	fibrillin-1-like	NaN	NaN	NaN	NaN	NaN	0.00	0.00	NaN	NaN	0.00	0.00	6	6	6	12.8	12.8	12.8	92.073	0	15.721	11399000	9	NaN	NaN	NaN	NaN	NaN	NaN	NaN	NaN
15	H9ISJ8	general odorant-binding protein 70-like	0.407791137695313	1.33	0	NaN	NaN	25.00	1.00	NaN	NaN	25.00	1.00	3	3	3	36.3	36.3	36.3	13.985	0	18.966	5807600	12	NaN	NaN	NaN	19.04	NaN	NaN	NaN	18.63
16	H9IT39	Uncharacterized protein OS	0.262575149536133	1.20	0.0305859883253986	20.10	20.10	50.00	2.00	19.84	19.84	50.00	2.00	12	12	12	55.8	55.8	55.8	35.354	0	50.397	81715000	29	NaN	21.76	NaN	18.45	NaN	NaN	17.68	22.00
17	H9ITH3	Peroxidase	NaN	NaN	NaN	NaN	NaN	0.00	0.00	NaN	NaN	0.00	0.00	6	6	6	6.8	6.8	6.8	151.53	0	13.507	7682100	10	NaN	NaN	NaN	NaN	NaN	NaN	NaN	NaN
18	H9ITR8	Chitinase-like protein EN03 OS	-1.35868708292643	-2.56	0.459625679760538	20.22	20.22	50.00	2.00	21.58	21.73	75.00	3.00	12	12	2	45.3	45.3	9.2	43.706	0	69.887	54621000	42	NaN	20.92	NaN	19.53	NaN	20.03	22.98	21.73
19	H9ITX3	procollagen-lysine,2-oxoglutarate 5-dioxygenase 1 isoform X1	NaN	NaN	NaN	NaN	NaN	0.00	0.00	NaN	NaN	0.00	0.00	3	3	3	8.6	8.6	8.6	51.878	0	8.4095	2081100	2	NaN	NaN	NaN	NaN	NaN	NaN	NaN	NaN
20	H9IU45;H9IU46	COesterase domain-containing protein OS	NaN	NaN	0	NaN	NaN	0.00	0.00	NaN	NaN	25.00	1.00	13	13	13	30.5	30.5	30.5	63.58	0	154.6	86398000	45	NaN	NaN	NaN	NaN	NaN	19.90	NaN	NaN
21	H9IV18	Sulfatase domain-containing protein OS	-2.12621307373047	-4.37	0	NaN	NaN	25.00	1.00	20.63	20.63	50.00	2.00	7	7	6	18.9	18.9	17.4	50.776	0	51.025	44617000	11	NaN	NaN	NaN	18.50	NaN	23.55	NaN	17.71
22	H9IV39	Rab GDP dissociation inhibitor OS	0.164913177490234	1.12	0.137900404140688	17.45	17.58	75.00	3.00	17.28	17.28	50.00	2.00	2	2	2	7.2	7.2	7.2	49.968	0	22.358	3968000	22	NaN	16.82	17.94	17.58	NaN	NaN	17.35	17.21
23	H9IVR6	Fibrohexamerin OS	-1.1791524887085	-2.26	0.437863714075233	25.52	26.00	100.00	4.00	26.70	27.27	100.00	4.00	10	10	2	51.8	51.8	9.1	25.149	0	74.443	3522600000	126	22.91	25.85	26.14	27.16	24.39	27.09	27.45	27.85
24	H9IVS0	Aldo-keto reductase OS	NaN	NaN	0	NaN	NaN	0.00	0.00	19.34	19.34	50.00	2.00	3	3	3	13.2	13.2	13.2	38.892	0	7.4199	4736200	5	NaN	NaN	NaN	NaN	NaN	NaN	19.52	19.17
25	H9IVS7	Uncharacterized protein OS	0.570236206054688	1.48	0.221119275484644	28.21	28.45	100.00	4.00	27.64	27.37	100.00	4.00	2	2	2	15.2	15.2	15.2	18.534	0	160.85	7412600000	59	27.81	26.78	29.09	29.15	26.33	26.10	28.41	29.71
26	H9IVS9	Uncharacterized protein OS	-1.47086572647095	-2.77	0.676826460591756	22.17	21.94	100.00	4.00	23.65	23.97	100.00	4.00	1	1	1	18.4	18.4	18.4	13.31	0	96.102	247260000	42	20.52	22.12	21.76	24.29	21.70	23.68	24.25	24.94
27	H9IW88;H9JP19;Q9XXZ4;H9JVY4	Ubiquitin family	0.341594219207764	1.27	0.371374203512393	23.06	22.97	100.00	4.00	22.72	22.88	100.00	4.00	5	5	5	52.3	52.3	52.3	17.217	0	59.725	226590000	47	23.02	22.92	23.63	22.67	23.36	22.97	21.76	22.78
28	H9IWH6	alpha-1,6-mannosyl-glycoprotein 2-beta-N-acetylglucosaminyltransferase isoform X1	NaN	NaN	0	NaN	NaN	0.00	0.00	19.49	19.49	50.00	2.00	3	3	3	13.6	13.6	13.6	46.722	0	35.658	15418000	10	NaN	NaN	NaN	NaN	NaN	NaN	18.71	20.27
29	H9IWH9	Uncharacterized protein OS	NaN	NaN	NaN	NaN	NaN	0.00	0.00	NaN	NaN	0.00	0.00	4	4	4	20.3	20.3	20.3	35.431	0	11.833	35933000	7	NaN	NaN	NaN	NaN	NaN	NaN	NaN	NaN
30	H9IWJ0	probable cyclin-dependent serine/threonine-protein kinase DDB	NaN	NaN	0	NaN	NaN	0.00	0.00	NaN	NaN	25.00	1.00	4	4	4	15.1	15.1	15.1	29.911	0	116.85	59666000	14	NaN	NaN	NaN	NaN	NaN	NaN	20.43	NaN
31	H9IWJ8	Carboxylic ester hydrolase OS	NaN	NaN	NaN	NaN	NaN	0.00	0.00	NaN	NaN	0.00	0.00	9	9	9	21.9	21.9	21.9	54.2	0	36.118	51024000	13	NaN	NaN	NaN	NaN	NaN	NaN	NaN	NaN
32	H9IX15	Uncharacterized protein OS	-0.446235974629719	-1.36	0.235563864310707	19.26	19.26	50.00	2.00	19.71	19.42	75.00	3.00	9	9	9	18.8	18.8	18.8	98.863	0	43.083	35696000	33	NaN	19.13	NaN	19.40	NaN	18.93	19.42	20.78
33	H9IX29	protein LLP homolog	NaN	NaN	0	NaN	NaN	0.00	0.00	19.43	19.43	50.00	2.00	4	4	4	24.1	24.1	24.1	26.875	0	33.891	22414000	12	NaN	NaN	NaN	NaN	NaN	NaN	18.84	20.03
34	H9IX30	Amino_oxidase domain-containing protein OS	-1.13750076293945	-2.20	0.31164426296723	20.41	20.41	50.00	2.00	21.55	21.76	75.00	3.00	5	5	5	28.5	28.5	28.5	28.612	0	19.713	113500000	27	NaN	19.84	NaN	20.98	NaN	19.60	21.76	23.28
35	H9IXK0	Antichymotrypsin-1 OS	-1.89270687103271	-3.71	1.05680487852107	18.33	18.33	50.00	2.00	20.22	20.22	50.00	2.00	5	5	2	18.8	18.8	6.1	41.918	0	12.761	11051000	8	NaN	18.00	NaN	18.65	NaN	NaN	20.72	19.71
36	H9IY55	Dnaj-20	NaN	NaN	0	NaN	NaN	0.00	0.00	18.02	18.02	50.00	2.00	5	5	5	16.5	16.5	16.5	54.479	0	26.669	7338700	14	NaN	NaN	NaN	NaN	NaN	NaN	17.71	18.34
37	H9IYS1	Thioredoxin domain-containing protein OS	NaN	NaN	0	NaN	NaN	0.00	0.00	18.45	18.45	50.00	2.00	3	3	3	32	32	32	22.038	0	5.02	9919600	6	NaN	NaN	NaN	NaN	NaN	NaN	18.65	18.25
38	H9IZ00	alpha 1,2-mannosidase	NaN	NaN	NaN	NaN	NaN	0.00	0.00	NaN	NaN	0.00	0.00	2	2	2	19.8	19.8	19.8	22.756	0	3.8616	1163800	3	NaN	NaN	NaN	NaN	NaN	NaN	NaN	NaN
39	H9IZ19	GTP-binding protein SAR1b	NaN	NaN	0	NaN	NaN	0.00	0.00	18.13	18.13	50.00	2.00	3	3	3	24.4	24.4	24.4	21.832	0	7.4107	7431600	8	NaN	NaN	NaN	NaN	NaN	NaN	18.58	17.68
40	H9IZ57	Tubulin alpha chain OS	0.655312061309814	1.57	0.749332700636318	22.02	21.97	100.00	4.00	21.36	21.47	100.00	4.00	14	14	5	55	55	23.8	49.948	0	147.66	112260000	74	22.01	21.54	22.60	21.93	20.38	22.13	21.64	21.31
41	H9IZE1;H9IZE2	general odorant-binding protein 56d-like	0.237597942352295	1.18	0.14320911539797	24.26	24.36	100.00	4.00	24.03	23.70	100.00	4.00	11	11	11	65.7	65.7	65.7	15.063	0	177.15	1033600000	127	24.12	22.96	25.37	24.61	23.74	23.66	23.55	25.15
42	H9IZE4	odorant binding protein LOC100301497 precursor	0.548959732055664	1.46	0	NaN	NaN	25.00	1.00	NaN	NaN	25.00	1.00	12	12	12	69.7	69.7	69.7	15.844	0	97.12	158340000	42	20.37	NaN	NaN	NaN	19.82	NaN	NaN	NaN
43	H9IZI4	pancreatic triacylglycerol lipase-like	NaN	NaN	0	NaN	NaN	0.00	0.00	19.28	19.28	50.00	2.00	4	4	4	7.2	7.2	7.2	67.566	0	8.3873	5529400	4	NaN	NaN	NaN	NaN	NaN	17.61	20.94	NaN
44	H9IZK4	seroin 1 precursor	0.773374557495117	1.71	0.518346720512494	29.75	29.53	100.00	4.00	28.98	28.97	100.00	4.00	7	7	7	83.1	83.1	83.1	7.6522	0	293.62	29589000000	229	29.09	28.77	29.96	31.19	28.06	29.42	29.92	28.51
45	H9IZL9	Lipocln_cytosolic_FA-bd_dom domain-containing protein OS	-0.921727180480957	-1.89	0.510875584902615	18.62	18.62	50.00	2.00	19.54	19.54	50.00	2.00	4	4	4	19.4	19.4	19.4	19.585	0	22.685	78354000	18	NaN	17.94	NaN	19.30	NaN	NaN	19.52	19.55
46	H9J012	Uncharacterized protein OS	-0.518580913543701	-1.43	0.196002408170237	20.70	20.05	100.00	4.00	21.21	21.57	100.00	4.00	10	10	10	13.4	13.4	13.4	86.703	0	75.545	66736000	39	20.65	19.21	23.48	19.45	20.13	21.56	21.58	21.59
47	H9J168	ZP domain-containing protein OS	0.0708651542663574	1.05	0.0317410287704327	19.24	19.17	100.00	4.00	19.17	19.17	50.00	2.00	4	4	4	12.3	12.3	12.3	41.649	0	12.141	14343000	14	18.98	18.22	19.35	20.41	NaN	NaN	18.65	19.69
48	H9J186	Chitin-binding type-2 domain-containing protein OS	NaN	NaN	0	NaN	NaN	0.00	0.00	18.52	18.52	50.00	2.00	4	4	4	3.9	3.9	3.9	117.83	0	8.2222	8701800	12	NaN	NaN	NaN	NaN	NaN	18.37	18.67	NaN
49	H9J1A5	SERPIN domain-containing protein OS	-1.46437899271647	-2.76	0	NaN	NaN	25.00	1.00	20.11	19.35	75.00	3.00	9	9	9	28.6	28.6	28.6	44.367	0	96.404	46800000	26	NaN	NaN	NaN	18.65	NaN	21.89	19.10	19.35
50	H9J1E3	probable uridine nucleosidase 2 isoform X1	NaN	NaN	0	NaN	NaN	0.00	0.00	18.78	18.78	50.00	2.00	12	12	12	54.5	54.5	54.5	36.001	0	187.41	1084000000	95	NaN	NaN	NaN	NaN	NaN	20.48	17.08	NaN
51	H9J1F8	juvenile hormone binding protein brP-1649 precursor	-1.51090431213379	-2.85	0.436700945367712	18.75	18.75	50.00	2.00	20.26	19.39	75.00	3.00	4	4	4	20.5	20.5	20.5	29.445	0	8.4655	16800000	10	NaN	18.18	NaN	19.32	NaN	19.04	22.35	19.39
52	H9J1X0	MCM domain-containing protein OS	-1.09704351425171	-2.14	0.457069735667228	21.02	21.20	100.00	4.00	22.11	22.79	100.00	4.00	14	14	14	12.1	12.1	12.1	167.88	0	163.32	138870000	53	19.97	21.71	20.72	21.67	19.26	22.25	23.34	23.61
53	H9J250	protein disulfide-isomerase like protein ERp57 precursor	-1.67905902862549	-3.20	0	NaN	NaN	25.00	1.00	20.14	20.14	50.00	2.00	9	9	9	28.3	28.3	28.3	48.813	0	34.477	13444000	10	18.47	NaN	NaN	NaN	NaN	NaN	18.54	21.74
54	H9J271;P29520;H9JDB9	Elongation factor 1-alpha OS;Elongation factor 1-alpha OS;Elongation factor 1-alpha OS	0.415050506591797	1.33	0.548876524300242	22.98	22.91	100.00	4.00	22.57	22.57	100.00	4.00	9	9	9	30.9	30.9	30.9	50.388	0	27.617	192310000	47	22.82	23.00	23.66	22.44	21.99	23.13	22.73	22.41
55	H9J2B8	Death domain-containing protein OS	-0.929911613464355	-1.91	0.1183828589348	18.99	18.99	50.00	2.00	19.92	19.25	75.00	3.00	10	10	5	27.7	27.7	15.9	65.01	0	50.059	60571000	32	NaN	18.73	NaN	19.26	NaN	16.56	19.25	23.96
56	H9J2I2	Uncharacterized protein OS	0.266263961791992	1.20	0	19.61	19.61	50.00	2.00	NaN	NaN	25.00	1.00	2	2	2	36	36	36	12.1	0	9.4493	6253300	7	NaN	18.87	NaN	20.36	NaN	NaN	19.35	NaN
57	H9J386;H9J3A7	Uncharacterized protein OS	-0.179073333740234	-1.13	0.945819852613347	18.94	18.98	75.00	3.00	19.12	19.12	50.00	2.00	8	8	8	9.3	9.3	9.3	125.58	0	22.658	86391000	34	NaN	18.98	19.02	18.82	NaN	NaN	19.14	19.09
58	H9J3H2	Peptidyl-prolyl cis-trans isomerase OS	-1.73700761795044	-3.33	0.525469897341285	21.64	21.13	100.00	4.00	23.38	24.37	100.00	4.00	12	12	12	66	66	66	21.893	0	158.1	312260000	105	20.69	21.43	20.82	23.63	19.35	25.42	24.36	24.39
59	H9J452;H9J453;H9J454	Uncharacterized protein OS;Uncharacterized protein OS;Uncharacterized protein OS	-1.64905834197998	-3.14	0	NaN	NaN	25.00	1.00	20.72	20.72	50.00	2.00	2	2	2	27.7	27.7	27.7	13.248	0	128.38	23935000	6	NaN	19.07	NaN	NaN	NaN	NaN	23.03	18.42
60	H9J4A1	salivary cysteine-rich peptide precursor	-1.5396105448405	-2.91	0.343462564070129	17.15	17.15	50.00	2.00	18.69	17.32	75.00	3.00	1	1	1	13	13	13	11.482	0	50.084	9506300	5	NaN	16.96	NaN	17.35	NaN	17.32	21.46	17.30
61	H9J4F6;P09336;Q00802	30K protein 1 OS;Low molecular 30 kDa lipoprotein PBMHPC-19 OS;Low molecular mass 30 kDa lipoprotein 19G1 OS	-1.54226175944011	-2.91	0.428017545713862	22.25	22.26	75.00	3.00	23.79	24.81	75.00	3.00	11	5	5	40.6	19.9	19.9	29.414	0	58.016	265340000	34	NaN	23.84	20.64	22.26	NaN	21.34	25.22	24.81
62	H9J4F9	Low molecular 30 kDa lipoprotein PBMHP-12 OS	-3.46083704630534	-11.01	0.774526235146152	22.16	23.47	100.00	4.00	25.62	26.14	75.00	3.00	20	20	3	66.8	66.8	16	29.251	0	323.31	841710000	128	17.45	24.23	22.71	24.23	NaN	23.35	27.36	26.14
63	H9J4G0;H9J4L5	AFC87801.1 30K protein 3	-2.86860227584839	-7.30	1.042037915302	21.38	21.45	100.00	4.00	24.25	24.38	75.00	3.00	15	15	7	39.1	39.1	18.8	43.772	0	323.31	332060000	73	20.48	23.01	19.63	22.41	NaN	22.12	26.25	24.38
64	H9J4K5	Peptidase S1 domain-containing protein OS	-1.01436201731364	-2.02	0.326182064447042	22.24	22.24	50.00	2.00	23.26	23.98	75.00	3.00	14	14	14	64.6	64.6	64.6	32.99	0	222.1	220860000	77	NaN	21.38	NaN	23.11	NaN	21.63	23.98	24.17
65	H9J4L6	low molecular mass 30 kDa lipoprotein 21G1-like	-2.89095687866211	-7.42	1.3144759820441	17.89	17.89	50.00	2.00	20.78	20.78	50.00	2.00	4	2	2	16.8	7.8	7.8	29.247	0	9.8702	16820000	6	NaN	18.37	NaN	17.40	NaN	NaN	21.23	20.33
66	H9J4N6;H9JVX1	microvitellogenin-like	NaN	NaN	0	NaN	NaN	0.00	0.00	NaN	NaN	25.00	1.00	10	10	8	37.9	37.9	30.4	34.692	0	51.423	869090000	46	NaN	NaN	NaN	NaN	21.45	NaN	NaN	NaN
67	H9J502	calsyntenin-1	NaN	NaN	NaN	NaN	NaN	0.00	0.00	NaN	NaN	0.00	0.00	3	3	3	5.9	5.9	5.9	99.422	0	4.8222	7636600	4	NaN	NaN	NaN	NaN	NaN	NaN	NaN	NaN
68	H9J524;F8UN43	Heat shock protein 68 OS	-0.155914942423504	-1.11	0.11190456774076	19.12	18.95	75.00	3.00	19.27	19.13	75.00	3.00	5	2	1	10.1	5	2.2	60.384	0	14.781	18185000	19	18.44	NaN	19.96	18.95	NaN	19.13	18.96	19.73
69	H9J585	Uncharacterized protein OS	-4.12332661946614	-17.43	1.56556880843814	19.12	19.61	75.00	3.00	23.24	24.11	100.00	4.00	38	38	38	42.5	42.5	42.5	140.76	0	323.31	981860000	291	17.78	19.96	NaN	19.61	20.19	24.55	24.10	24.12
70	H9J593	Carboxylic ester hydrolase OS	-3.43243217468262	-10.80	0	NaN	NaN	25.00	1.00	19.18	19.18	50.00	2.00	11	11	11	30.5	30.5	30.5	53.787	0	53.622	13678000	12	NaN	15.75	NaN	NaN	NaN	NaN	22.42	15.94
71	H9J5D6	serine protease inhibitor 28 isoform X2	0.0879926681518555	1.06	0.0491843446337505	22.94	22.93	100.00	4.00	22.85	22.94	100.00	4.00	9	5	5	36.5	24.6	24.6	31.922	0	259.37	1608100000	137	22.18	23.68	21.94	23.95	21.91	22.77	23.62	23.11
72	H9J5D7	Uncharacterized protein OS	0.332019329071045	1.26	0.418141263029174	25.65	25.71	100.00	4.00	25.32	25.42	100.00	4.00	13	13	13	49.1	49.1	49.1	30.031	0	215.83	4035300000	187	25.97	24.97	26.20	25.46	24.73	25.21	25.70	25.62
73	H9J5D8	Uncharacterized protein OS	0.124201774597168	1.09	0.10669358629154	26.58	26.54	100.00	4.00	26.46	26.50	100.00	4.00	37	37	33	60.4	60.4	55.8	80.34	0	323.31	10648000000	434	26.58	26.51	26.86	26.38	25.39	26.45	27.43	26.55
74	H9J5E5	SERPIN domain-containing protein OS	-1.20991516113281	-2.31	0	NaN	NaN	25.00	1.00	20.04	20.04	50.00	2.00	3	3	3	5.5	5.5	5.5	80.194	0	5.9025	8873400	6	NaN	NaN	NaN	18.83	NaN	19.08	21.01	NaN
75	H9J5L6	Uncharacterized protein OS	-4.91066694259644	-30.08	0	NaN	NaN	25.00	1.00	20.42	20.42	50.00	2.00	36	36	36	21.9	21.9	21.9	315.47	0	323.31	102880000	50	NaN	15.51	NaN	NaN	NaN	NaN	25.30	15.53
76	H9J5L9	Uncharacterized protein OS	-0.376588821411133	-1.30	0.383820781435838	21.27	21.27	75.00	3.00	21.64	21.30	75.00	3.00	4	4	4	31.2	31.2	31.2	16.971	0	50.708	239540000	36	21.50	21.27	NaN	21.03	NaN	21.21	22.42	21.30
77	H9J657	Ig_mannosidase domain-containing protein OS	NaN	NaN	0	NaN	NaN	0.00	0.00	21.11	20.85	75.00	3.00	11	11	11	17.3	17.3	17.3	101.55	0	39.445	40243000	35	NaN	NaN	NaN	NaN	NaN	20.85	20.35	22.12
78	H9J6C7	Uncharacterized protein OS	0.652673244476318	1.57	0.421373683244911	26.88	26.73	100.00	4.00	26.22	26.37	100.00	4.00	22	22	22	57.4	57.4	57.4	50.924	0	323.31	19649000000	371	28.37	26.25	25.68	27.20	25.23	26.37	26.92	26.37
79	H9J6I9	Uncharacterized protein OS	-1.63650862375895	-3.11	1.31890586828802	20.04	20.15	100.00	4.00	21.67	22.22	75.00	3.00	18	18	18	13.6	13.6	13.6	206.84	0	120.16	80964000	56	19.10	19.89	20.75	20.41	NaN	20.57	22.22	22.24
80	H9J709	Uncharacterized protein OS	-0.861912727355957	-1.82	0.201581681986499	21.04	20.91	100.00	4.00	21.91	23.35	100.00	4.00	7	7	7	51.2	51.2	51.2	26.803	0	40.129	116960000	36	19.86	19.65	21.95	22.71	17.37	23.30	23.40	23.55
81	H9J730	COesterase domain-containing protein OS	NaN	NaN	0	NaN	NaN	0.00	0.00	NaN	NaN	25.00	1.00	5	5	5	36.1	36.1	36.1	21.032	0	23.458	14590000	13	NaN	NaN	NaN	NaN	NaN	NaN	NaN	17.44
82	H9J7K1	Similar to enolase	NaN	NaN	0	NaN	NaN	0.00	0.00	19.52	19.52	50.00	2.00	5	5	5	14.8	14.8	14.8	47.053	0	22.473	4476000	7	NaN	NaN	NaN	NaN	NaN	NaN	19.86	19.17
83	H9J7K8	Beta-glucuronidase OS	-0.217092037200928	-1.16	0.0898836213045784	18.86	18.68	100.00	4.00	19.08	19.07	75.00	3.00	22	22	22	47.4	47.4	47.4	61.022	0	204.55	476650000	122	17.64	18.88	18.49	20.45	NaN	19.07	20.17	18.01
84	H9J7R4	Peptidase_M28 domain-containing protein OS	-1.27513154347738	-2.42	0.948897798526315	19.72	19.44	100.00	4.00	20.99	21.11	75.00	3.00	9	9	9	41.8	41.8	41.8	33.511	0	21.663	63529000	47	19.23	19.64	19.01	20.98	NaN	20.09	21.11	21.77
85	H9J7X3	PKS_ER domain-containing protein OS	NaN	NaN	NaN	NaN	NaN	0.00	0.00	NaN	NaN	0.00	0.00	4	4	4	24.3	24.3	24.3	25.991	0	37.133	10120000	16	NaN	NaN	NaN	NaN	NaN	NaN	NaN	NaN
86	H9J850;H9JI92	GMC_oxred_C domain-containing protein OS	NaN	NaN	NaN	NaN	NaN	0.00	0.00	NaN	NaN	0.00	0.00	3	3	3	17.8	17.8	17.8	31.44	0	6.3132	5935700	5	NaN	NaN	NaN	NaN	NaN	NaN	NaN	NaN
87	H9J868	GMC_OxRdtase_N domain-containing protein OS	-0.206106185913086	-1.15	0.0469019377906532	21.09	20.83	100.00	4.00	21.29	22.04	100.00	4.00	28	28	27	57.3	57.3	55.6	65.475	0	311.21	1048700000	185	19.10	21.31	20.34	23.60	17.82	22.45	23.28	21.62
88	H9J894	fibulin-1-like	-3.34424018859863	-10.16	1.23777850446945	20.05	19.54	100.00	4.00	23.39	24.28	100.00	4.00	26	26	26	31.6	31.6	31.6	133.02	0	323.31	472250000	155	18.48	19.72	22.64	19.36	20.10	24.27	24.29	24.92
89	H9J8B3	SHSP domain-containing protein OS	-1.12034670511882	-2.17	0.662951007446323	20.77	21.01	75.00	3.00	21.89	22.53	75.00	3.00	7	7	7	32.8	32.8	32.8	26.587	0	18.257	233160000	36	NaN	21.01	20.23	21.06	NaN	20.46	22.53	22.68
90	H9J8H0	Arginine kinase	-2.39073085784912	-5.24	0	NaN	NaN	25.00	1.00	21.26	21.26	50.00	2.00	10	10	10	30.4	30.4	30.4	50.27	0	27.018	20867000	18	NaN	18.86	NaN	NaN	NaN	NaN	20.35	22.16
91	H9J8I5;H9J8I4	15-hydroxyprostaglandin dehydrogenase [D(+)]	NaN	NaN	0	NaN	NaN	0.00	0.00	NaN	NaN	25.00	1.00	3	3	3	11.5	11.5	11.5	31.689	0	7.1653	4944600	6	NaN	NaN	NaN	NaN	NaN	NaN	NaN	16.41
92	H9J8I6	alcohol dehydrogenase 2	-0.0249676704406738	-1.02	0.00844986368385632	25.03	24.90	100.00	4.00	25.06	25.77	100.00	4.00	20	20	20	75.5	75.5	75.5	35.344	0	165.78	2796900000	219	25.10	24.70	24.33	26.01	22.37	25.32	26.33	26.21
93	H9J8I8	alcohol dehydrogenase precursor	NaN	NaN	NaN	NaN	NaN	0.00	0.00	NaN	NaN	0.00	0.00	9	2	2	34.4	14.7	14.7	31.336	0	14.536	32082000	15	NaN	NaN	NaN	NaN	NaN	NaN	NaN	NaN
94	H9J8I9	alcohol dehydrogenase precursor	-1.01958322525024	-2.03	0.763712740738704	21.66	21.73	100.00	4.00	22.68	22.72	75.00	3.00	16	16	5	58.8	58.8	30.1	32.021	0	296.72	1037800000	104	21.05	22.41	20.47	22.70	NaN	22.88	22.72	22.43
95	H9J8J0	15-hydroxyprostaglandin dehydrogenase [D(+)]	-0.1900954246521	-1.14	0.102735405490193	21.58	21.68	100.00	4.00	21.77	21.73	100.00	4.00	14	9	9	59.2	45	45	31.745	0	172.67	1141700000	82	21.25	22.10	20.46	22.51	20.74	21.12	22.34	22.88
96	H9J9I7	15-hydroxyprostaglandin dehydrogenase [D(+)]	-2.60310490926107	-6.08	0.827916695481615	18.60	18.60	50.00	2.00	21.20	20.84	75.00	3.00	19	19	19	22.6	22.6	22.6	137.38	0	192.96	162680000	91	NaN	17.51	NaN	19.68	NaN	19.97	20.84	22.79
97	H9J9P2	balbiani ring protein 3-like isoform X3	0.554196834564209	1.47	0.30188149941233	25.25	25.22	100.00	4.00	24.69	24.84	100.00	4.00	14	14	14	77.4	77.4	77.4	26.912	0	234	1861300000	222	25.25	23.58	26.98	25.18	23.76	24.82	24.86	25.34
98	H9J9P6	Uncharacterized protein OS	NaN	NaN	0	NaN	NaN	0.00	0.00	19.61	19.40	75.00	3.00	2	2	2	26.7	26.7	26.7	9.9994	0	8.772	6159200	4	NaN	NaN	NaN	NaN	NaN	19.40	20.40	19.04
99	H9J9Q8	TIL domain-containing protein OS	-0.206595897674561	-1.15	0.137309106443269	22.33	22.03	100.00	4.00	22.54	22.70	100.00	4.00	5	5	5	56.1	56.1	56.1	6.9021	0	27.23	411160000	44	22.43	21.46	23.81	21.63	22.67	21.97	22.73	22.80
100	H9JA74	Malic enzyme OS	-1.72412586212158	-3.30	0.366002086722457	18.72	18.72	50.00	2.00	20.45	21.51	100.00	4.00	8	8	8	18.6	18.6	18.6	60.14	0	51.755	30836000	20	NaN	19.57	NaN	17.88	16.67	21.61	22.09	21.42
101	H9JAH3	Uncharacterized protein OS	0.163957595825195	1.12	0	NaN	NaN	25.00	1.00	NaN	NaN	25.00	1.00	6	6	6	10.6	10.6	10.6	84.452	0	32.465	4180600	11	NaN	17.83	NaN	NaN	NaN	NaN	17.67	NaN
102	H9JB50	Uncharacterized protein OS	NaN	NaN	0	NaN	NaN	0.00	0.00	20.32	20.60	75.00	3.00	7	7	7	13.5	13.5	13.5	78.347	0	34.909	17307000	16	NaN	NaN	NaN	NaN	NaN	19.02	20.60	21.33
103	H9JBC0	Beta-galactosidase OS	0.573026180267334	1.49	0.118510262183083	21.96	22.20	100.00	4.00	21.38	22.38	100.00	4.00	23	23	23	34.2	34.2	34.2	66.671	0	167.12	512360000	132	20.58	22.75	21.65	22.84	16.43	21.97	22.79	24.34
104	H9JBF8	Uncharacterized protein OS	0.151576995849609	1.11	0.197606330115263	23.64	23.64	50.00	2.00	23.49	23.46	75.00	3.00	2	2	2	2.2	2.2	2.2	70.518	0	3.3748	587190000	7	NaN	NaN	23.94	23.34	23.74	23.26	23.46	NaN
105	H9JBL0	Fucosidase_C domain-containing protein OS	-1.62242190043132	-3.08	1.08452599649159	18.50	18.50	50.00	2.00	20.13	19.90	75.00	3.00	7	7	7	18.9	18.9	18.9	61.827	0	16.033	29517000	18	NaN	18.73	NaN	18.28	NaN	19.90	19.45	21.03
106	H9JBL2;Q1HQ47	Isocitrate dehydrogenase [NADP] OS	NaN	NaN	0	NaN	NaN	0.00	0.00	17.92	17.92	50.00	2.00	3	3	3	16.2	16.2	16.2	46.16	0	4.8196	1951200	4	NaN	NaN	NaN	NaN	NaN	NaN	18.30	17.53
107	H9JBP6	Thioredoxin domain-containing protein OS	-3.06940269470215	-8.39	1.29780247507404	18.67	18.90	100.00	4.00	21.74	22.18	100.00	4.00	16	16	16	51.3	51.3	51.3	39.268	0	48.385	135380000	66	18.71	17.48	19.09	19.42	18.48	22.37	24.13	21.99
108	H9JBW7	Fam20C domain-containing protein OS	NaN	NaN	0	NaN	NaN	0.00	0.00	21.84	21.89	75.00	3.00	10	10	10	31.9	31.9	31.9	51.434	0	29.781	64245000	40	NaN	NaN	NaN	NaN	NaN	21.39	22.23	21.89
109	H9JBZ4	Inhibitor_I29 domain-containing protein OS	-0.3094482421875	-1.24	0.112507520744537	21.75	21.50	100.00	4.00	22.06	22.62	100.00	4.00	7	7	7	60.4	60.4	60.4	10.79	0	90.505	148140000	53	20.44	21.23	23.57	21.77	19.80	22.44	22.79	23.23
110	H9JBZ7	Uncharacterized protein OS	-1.50157483418783	-2.83	0.970980831620223	19.46	19.48	75.00	3.00	20.96	21.50	75.00	3.00	22	22	22	23.1	23.1	23.1	150.98	0	71.569	58307000	44	NaN	18.97	19.94	19.48	NaN	19.64	21.76	21.50
111	H9JC16	Pept_C1 domain-containing protein OS	0.62704308827718	1.54	0.101051665382321	24.03	23.87	75.00	3.00	23.41	24.32	100.00	4.00	19	19	19	57.9	57.9	57.9	37.56	0	323.31	803210000	175	NaN	23.87	23.37	24.85	18.55	22.30	26.44	26.33
112	H9JC27	yellow-b	-0.695773442586262	-1.62	0.249850763291576	20.94	20.94	50.00	2.00	21.64	21.51	75.00	3.00	10	10	10	35.1	35.1	35.1	49.684	0	201.9	106300000	70	NaN	20.43	NaN	21.46	NaN	20.37	21.51	23.04
113	H9JCK9	ATP synthase subunit O, mitochondrial isoform X1	0.322422504425049	1.25	0.116421863867157	24.86	24.87	100.00	4.00	24.53	24.41	100.00	4.00	17	17	17	43.8	43.8	43.8	53.031	0	251.29	3014000000	105	24.73	25.00	24.20	25.49	22.36	23.60	26.94	25.22
114	H9JDE9;H9JDF0	Carboxylic ester hydrolase OS	0.446070671081543	1.36	0.111989674507504	20.65	20.86	100.00	4.00	20.21	20.47	100.00	4.00	19	19	19	43.8	43.8	43.8	61.701	0	199.06	557980000	135	21.45	20.43	19.44	21.28	16.96	18.80	22.14	22.92
115	H9JDV4	Nucleoside diphosphate kinase OS	NaN	NaN	0	NaN	NaN	0.00	0.00	18.87	18.87	50.00	2.00	4	4	4	35.3	35.3	35.3	17.182	0	6.4535	3811800	7	NaN	NaN	NaN	NaN	NaN	NaN	18.72	19.02
116	H9JDX3	serine protease inhibitor 2 isoform X1	-0.234428405761719	-1.18	0	NaN	NaN	25.00	1.00	19.71	19.71	50.00	2.00	4	4	4	12.4	12.4	12.4	38.699	0	9.9308	12970000	7	NaN	NaN	19.48	NaN	NaN	NaN	20.06	19.36
117	H9JET3	Allantoate amidinohydrolas	NaN	NaN	0	NaN	NaN	0.00	0.00	19.32	19.32	50.00	2.00	4	4	4	13.8	13.8	13.8	38.481	0	7.7191	13108000	7	NaN	NaN	NaN	NaN	NaN	NaN	19.15	19.49
118	H9JFR3	gamma-interferon-inducible lysosomal thiol reductase-like [Papilio xuthus]	-1.51121536890666	-2.85	0.701836783171584	20.13	19.99	75.00	3.00	21.64	21.60	100.00	4.00	9	9	9	65.4	65.4	65.4	23.186	0	56.19	99733000	50	NaN	18.98	21.41	19.99	19.98	21.26	21.93	23.37
119	H9JGT9	Uncharacterized protein OS	1.4387690226237	2.71	0	NaN	NaN	25.00	1.00	21.63	19.86	75.00	3.00	17	17	17	60.3	60.3	60.3	41.717	0	315.53	242080000	25	NaN	23.07	NaN	NaN	NaN	18.57	26.46	19.86
120	H9JGX1	Ferritin OS	-0.0454762776692696	-1.03	0	NaN	NaN	25.00	1.00	18.69	18.60	75.00	3.00	5	5	5	30.1	30.1	30.1	19.526	0	11.785	20107000	14	NaN	18.64	NaN	NaN	17.46	18.60	NaN	20.00
121	H9JGY3	Ferritin OS	-0.51173210144043	-1.43	0.623820172645629	19.12	19.12	50.00	2.00	19.63	19.58	75.00	3.00	7	7	7	38.9	38.9	38.9	26.061	0	42.029	42486000	23	19.57	NaN	NaN	18.66	NaN	19.58	19.73	19.57
122	H9JH30	serine protease inhibitor 20 precursor	-0.970556259155273	-1.96	0	NaN	NaN	25.00	1.00	18.73	17.75	75.00	3.00	7	7	7	19.9	19.9	19.9	39.528	0	18.258	6864700	9	NaN	NaN	NaN	17.76	NaN	17.75	21.19	17.26
123	H9JH31	SERPIN domain-containing protein OS	-2.1592534383138	-4.47	0.870214796353039	20.71	20.96	75.00	3.00	22.87	22.29	75.00	3.00	9	9	9	51.5	51.5	51.5	18.475	0	103.03	272830000	52	19.60	20.96	NaN	21.57	NaN	22.29	21.50	24.81
124	H9JH32	alaserpin-like	-1.20341571172078	-2.30	0.616579263111925	21.39	21.72	100.00	4.00	22.59	22.02	75.00	3.00	10	10	10	47.5	47.5	47.5	24.231	0	70.668	582240000	61	21.75	21.69	20.37	21.75	NaN	22.02	21.27	24.49
125	H9JH50	Uncharacterized protein OS	NaN	NaN	0	NaN	NaN	0.00	0.00	18.59	18.59	50.00	2.00	2	2	2	23.6	23.6	23.6	17.871	0	9.1119	8801900	5	NaN	NaN	NaN	NaN	NaN	NaN	18.05	19.14
126	H9JH97	ADP-ribosylation factor 2	-0.0818328857421875	-1.06	0.148871496685401	17.68	17.68	50.00	2.00	17.77	17.77	50.00	2.00	3	3	1	27.6	27.6	6.1	18.498	0	15.742	7415500	8	NaN	NaN	17.87	17.49	NaN	17.76	NaN	17.77
127	H9JHU5	Trypsin Inhibitor like cysteine rich domain	1.12655289967855	2.18	0.379520919360147	24.13	24.13	50.00	2.00	23.00	23.21	75.00	3.00	3	3	3	69.1	69.1	69.1	5.9337	0	11.715	246850000	13	25.50	NaN	NaN	22.76	22.08	23.72	NaN	23.21
128	H9JI83	Aldose 1-epimerase OS	NaN	NaN	0	NaN	NaN	0.00	0.00	18.19	18.19	50.00	2.00	3	3	3	17.6	17.6	17.6	39.711	0	40.008	3729900	4	NaN	NaN	NaN	NaN	NaN	NaN	16.97	19.41
129	H9JIB1	Uncharacterized protein OS	-0.491569995880127	-1.41	0.174340810472282	23.16	23.43	100.00	4.00	23.66	24.44	100.00	4.00	6	6	6	33	33	33	21.838	0	31.001	921770000	92	23.47	23.38	21.65	24.15	20.84	24.10	24.91	24.78
130	H9JIB3	Uncharacterized protein OS	NaN	NaN	0	NaN	NaN	0.00	0.00	NaN	NaN	25.00	1.00	5	5	5	6.6	6.6	6.6	122.7	0	13.639	10839000	5	NaN	NaN	NaN	NaN	NaN	NaN	NaN	18.42
131	H9JIY7	venom acid phosphatase Acph-1-like	-0.48623498280843	-1.40	0.104247284972311	19.39	19.79	75.00	3.00	19.88	20.53	100.00	4.00	9	9	9	33.2	33.2	33.2	45.142	0	128.12	97753000	46	NaN	20.86	17.54	19.79	16.34	19.93	21.14	22.11
132	H9JJ43	Carboxylic ester hydrolase OS	-0.941205024719238	-1.92	0	NaN	NaN	25.00	1.00	22.35	22.35	50.00	2.00	11	11	11	25.6	25.6	25.6	71.943	0	102	75915000	24	NaN	21.41	NaN	NaN	NaN	NaN	24.62	20.08

133	H9JJP9	Peptidase S1 domain-containing protein OS	NaN	NaN	0	NaN	NaN	0.00	0.00	19.19	19.19	50.00	2.00	4	4	4	10.6	10.6	10.6	69.144	0	10.184	4436900	6	NaN	NaN	NaN	NaN	NaN	NaN	19.45	18.93
134	H9JJU8;H9JJU9	Aldo_ket_red domain-containing protein OS	-0.520703633626301	-1.43	0.0838323475661753	20.48	19.73	75.00	3.00	21.00	20.95	75.00	3.00	11	11	11	32.3	32.3	32.3	41.843	0	93.299	78331000	25	NaN	23.65	18.06	19.73	NaN	18.52	20.95	23.53
135	H9JK23	Carboxylic ester hydrolase OS	-2.65096791585286	-6.28	0.8618994801901	19.72	19.72	50.00	2.00	22.37	22.72	75.00	3.00	13	13	13	36.4	36.4	36.4	61.619	0	44.187	90094000	32	NaN	20.20	NaN	19.24	NaN	20.52	22.72	23.87
136	H9JK39	Glyco_18 domain-containing protein OS	NaN	NaN	0	NaN	NaN	0.00	0.00	NaN	NaN	25.00	1.00	7	7	7	41.8	41.8	41.8	23.483	0	52.317	79139000	34	NaN	NaN	NaN	NaN	NaN	NaN	NaN	16.57
137	H9JK40	Chitin-binding type-2 domain-containing protein OS	NaN	NaN	NaN	NaN	NaN	0.00	0.00	NaN	NaN	0.00	0.00	9	9	9	4.3	4.3	4.3	211.87	0	26.679	78687000	27	NaN	NaN	NaN	NaN	NaN	NaN	NaN	NaN
138	H9JK41	Chitin-binding type-2 domain-containing protein OS	NaN	NaN	NaN	NaN	NaN	0.00	0.00	NaN	NaN	0.00	0.00	5	5	5	11.2	11.2	11.2	67.314	0	72.532	52526000	26	NaN	NaN	NaN	NaN	NaN	NaN	NaN	NaN
139	H9JKA2;P22922	Antichymotrypsin-2 OS;Antitrypsin OS	-3.04728539784749	-8.27	1.26721631052318	20.90	20.23	100.00	4.00	23.95	23.88	75.00	3.00	14	14	1	43.6	43.6	5	37.745	0	323.31	232710000	30	20.14	23.24	20.32	19.91	NaN	23.88	25.61	22.36
140	H9JKT2	SGL domain-containing protein OS	-0.522937774658203	-1.44	0	NaN	NaN	25.00	1.00	NaN	NaN	25.00	1.00	4	4	4	28.2	28.2	28.2	29.839	0	15.944	10993000	12	NaN	17.45	NaN	NaN	NaN	NaN	17.97	NaN
141	H9JL11	SERPIN domain-containing protein OS	-2.06098254521688	-4.17	0.481734257952255	20.70	21.38	75.00	3.00	22.77	24.00	100.00	4.00	21	21	21	59.6	59.6	59.6	51.598	0	323.31	203130000	65	NaN	21.38	19.29	21.44	18.27	23.33	24.79	24.67
142	H9JL76	venom acid phosphatase Acph-1-like	NaN	NaN	0	NaN	NaN	0.00	0.00	19.39	18.41	75.00	3.00	4	4	4	18.8	18.8	18.8	26.845	0	24.5	19347000	16	NaN	NaN	NaN	NaN	NaN	18.41	17.89	21.86
143	H9JLC2	Uncharacterized protein OS	-1.59456062316895	-3.02	0	NaN	NaN	25.00	1.00	19.76	19.78	75.00	3.00	4	4	4	31.3	31.3	31.3	21.769	0	28.893	30598000	27	NaN	18.16	NaN	NaN	NaN	19.42	19.78	20.07
144	H9JLC3	ZP domain-containing protein OS	-2.31364186604818	-4.97	0	NaN	NaN	25.00	1.00	20.70	21.06	75.00	3.00	9	9	9	37.5	37.5	37.5	36.719	0	117.85	37298000	33	NaN	18.39	NaN	NaN	NaN	19.88	21.06	21.16
145	H9JLM2	Malate dehydrogenase OS	NaN	NaN	0	NaN	NaN	0.00	0.00	18.12	18.12	50.00	2.00	2	2	2	7.6	7.6	7.6	35.435	0	3.614	1852200	4	NaN	NaN	NaN	NaN	NaN	NaN	18.13	18.11
146	H9JLW4;H9JLW5;H9JWN7	GMC_OxRdtase_N domain-containing protein OS	-3.15064160029094	-8.88	1.81506833798031	22.18	22.23	100.00	4.00	25.33	25.34	75.00	3.00	22	21	10	57.8	55.7	25.2	51.954	0	271.64	719430000	168	21.26	23.19	20.66	23.60	NaN	24.91	25.73	25.34
147	H9JMY6;H9JMY9	zonadhesin isoform X1	0.147010326385498	1.11	0.0440805554625863	25.57	25.68	100.00	4.00	25.42	25.42	100.00	4.00	19	19	19	58.8	58.8	58.8	40.209	0	323.31	1335600000	196	26.99	23.10	27.81	24.37	25.19	24.54	25.65	26.30
148	H9JMY7	TIL domain-containing protein OS	NaN	NaN	NaN	NaN	NaN	0.00	0.00	NaN	NaN	0.00	0.00	3	3	3	36.4	36.4	36.4	9.6721	0	49.548	230570000	15	NaN	NaN	NaN	NaN	NaN	NaN	NaN	NaN
149	H9JMY8	TIL domain-containing protein OS	-0.110466321309406	-1.08	0.0126682214506043	22.81	24.29	75.00	3.00	22.92	22.92	50.00	2.00	2	2	2	35.4	35.4	35.4	10.81	0	40.157	698970000	37	24.29	18.51	25.63	NaN	NaN	NaN	23.15	22.69
150	H9JN73	Carboxylic ester hydrolase OS	0.210079193115234	1.16	0.131415682486433	18.91	18.84	75.00	3.00	18.70	18.56	75.00	3.00	5	5	5	13.4	13.4	13.4	61.127	0	10.471	6687000	6	18.84	18.27	NaN	19.62	NaN	18.56	19.52	18.01
151	H9JN76	Uncharacterized protein OS	-1.80440298716227	-3.49	0.864345521378932	18.93	18.78	100.00	4.00	20.73	20.71	75.00	3.00	6	6	6	34.4	34.4	34.4	23.487	0	94.699	25884000	27	18.47	18.30	19.10	19.84	NaN	20.71	22.67	18.82
152	H9JPD2	lachesin isoform X2	NaN	NaN	0	NaN	NaN	0.00	0.00	19.26	19.35	75.00	3.00	2	2	2	3.1	3.1	3.1	106.64	0	15.384	9786100	6	NaN	NaN	NaN	NaN	NaN	19.35	18.48	19.96
153	H9JPD3	lachesin isoform X2	NaN	NaN	0	NaN	NaN	0.00	0.00	21.40	21.30	75.00	3.00	5	5	5	27.1	27.1	27.1	22.976	0	18.534	69415000	15	NaN	NaN	NaN	NaN	NaN	21.63	21.28	21.30
154	H9JPE5	SVWC domain-containing protein OS	0.56846809387207	1.48	0	NaN	NaN	25.00	1.00	18.46	18.39	75.00	3.00	1	1	1	16.3	16.3	16.3	11.442	0	26.238	4615100	8	NaN	NaN	NaN	19.02	NaN	18.39	18.32	18.66
155	H9JPG9	Transferrin OS	NaN	NaN	0	NaN	NaN	0.00	0.00	NaN	NaN	25.00	1.00	4	4	4	9.3	9.3	9.3	71.651	0	11.837	7132700	5	NaN	NaN	NaN	NaN	NaN	NaN	19.34	NaN
156	H9JPS6	Varsurf_PPLC domain-containing protein OS	0.0167535146077498	1.01	0.00870750946178482	19.43	19.30	75.00	3.00	19.42	19.77	75.00	3.00	12	12	12	41.6	41.6	41.6	49.839	0	85.646	165920000	56	NaN	18.94	19.30	20.07	18.36	NaN	19.77	20.13
157	H9JPW9	Uncharacterized protein OS	NaN	NaN	0	NaN	NaN	0.00	0.00	20.25	20.25	50.00	2.00	2	2	2	8.3	8.3	8.3	64.38	0	5.9591	4158500	3	NaN	NaN	NaN	NaN	NaN	20.70	19.80	NaN
158	H9JQ04	GH16 domain-containing protein OS	-2.09204959869385	-4.26	0	NaN	NaN	25.00	1.00	19.73	19.73	50.00	2.00	5	5	5	18.3	18.3	18.3	47.632	0	49.638	13811000	7	NaN	17.64	NaN	NaN	NaN	NaN	21.69	17.78
159	H9JQ40	Beta-hexosaminidase OS	-0.0543789863586426	-1.04	0.0221737241562839	25.95	25.96	100.00	4.00	26.00	26.31	100.00	4.00	30	30	30	49.1	49.1	49.1	72.147	0	323.31	1107200000[illegible]	585	25.09	26.35	25.57	26.78	23.93	26.09	26.54	27.44
160	H9JQ96	EN protein binding/engrailed nuclear homeoprotein-regulated protein	NaN	NaN	0	NaN	NaN	0.00	0.00	19.89	19.67	75.00	3.00	6	6	6	48.1	48.1	48.1	26.161	0	14.912	8965300	12	NaN	NaN	NaN	NaN	NaN	19.67	19.16	20.83
161	H9JQN8	Protein disulfide-isomerase OS	-2.9526252746582	-7.74	1.25354174768994	20.46	20.59	100.00	4.00	23.42	23.91	100.00	4.00	20	20	20	44.7	44.7	44.7	55.584	0	186.83	292490000	87	21.58	20.28	19.09	20.90	20.34	23.20	25.51	24.62
162	H9JQU0	Uncharacterized protein OS	1.75939146677653	3.39	0.360786779355661	23.30	24.11	75.00	3.00	21.54	23.01	100.00	4.00	52	52	52	49.5	49.5	49.5	177.61	0	323.31	2172400000	373	24.48	21.31	NaN	24.11	22.96	23.07	23.40	16.73
163	H9JQU5	sericin 2 precursor	-0.175334771474201	-1.13	0.0279669776504867	20.49	19.22	75.00	3.00	20.66	20.81	100.00	4.00	14	14	14	14.4	14.4	14.4	125.2	0	153.15	99968000	59	25.16	17.08	NaN	19.22	19.24	20.25	21.37	21.79
164	H9JR22	low-density lipoprotein receptor-related protein 4-like	-1.74154790242513	-3.34	0	NaN	NaN	25.00	1.00	18.40	18.79	75.00	3.00	24	24	24	7.8	7.8	7.8	479.7	0	68.257	38138000	52	NaN	16.66	NaN	NaN	NaN	17.55	18.79	18.85
165	H9JR42	sericin 3	0.0138230323791504	1.01	0.00536151290399933	26.39	26.85	100.00	4.00	26.37	26.63	100.00	4.00	7	7	7	29.3	29.3	29.3	32.802	0	323.31	5998900000	100	27.65	24.19	27.51	26.20	26.68	25.43	26.81	26.59
166	H9JRI2	mannosyl-oligosaccharide glucosidase	NaN	NaN	NaN	NaN	NaN	0.00	0.00	NaN	NaN	0.00	0.00	3	3	3	4.6	4.6	4.6	107.74	0	7.1531	5175800	4	NaN	NaN	NaN	NaN	NaN	NaN	NaN	NaN
167	H9JRT0	basement membrane-specific heparan sulfate proteoglycan core protein-like isoform X3	-0.564088503519695	-1.48	0.217985264761719	19.37	19.67	100.00	4.00	19.94	20.81	75.00	3.00	25	25	25	11.3	11.3	11.3	425.72	0	180.6	52333000	71	19.16	20.18	17.82	20.33	18.12	NaN	20.81	20.88
168	H9JRZ9	D-3-phosphoglycerate dehydrogenase	NaN	NaN	0	NaN	NaN	0.00	0.00	18.72	18.72	50.00	2.00	3	3	3	8.1	8.1	8.1	46.581	0	5.1533	3844000	8	NaN	NaN	NaN	NaN	NaN	NaN	18.78	18.67
169	H9JS17	Uncharacterized protein OS	0.135478496551514	1.10	0.297653796540759	19.83	19.79	100.00	4.00	19.70	19.70	50.00	2.00	2	2	2	0.6	0.6	0.6	449.08	0.0022272	2.5045	30237000	3	19.78	19.97	19.79	19.78	NaN	19.42	NaN	19.98
170	H9JSJ9	Sulfatase domain-containing protein OS	0.746551354726154	1.68	0.271608787480157	18.25	18.78	75.00	3.00	17.51	17.04	100.00	4.00	22	22	22	59.8	59.8	59.8	42.345	0	95.454	622200000	109	19.09	16.88	NaN	18.78	16.11	17.22	19.84	16.85
171	H9JSK0	Sulfatase domain-containing protein OS	NaN	NaN	NaN	NaN	NaN	0.00	0.00	NaN	NaN	0.00	0.00	3	2	1	5.1	3.8	1.7	60.177	0	4.0783	25639000	12	NaN	NaN	NaN	NaN	NaN	NaN	NaN	NaN
172	H9JSY0	ATP-dependent (S)-NAD(P)H-hydrate dehydratase OS	NaN	NaN	0	NaN	NaN	0.00	0.00	21.01	20.93	75.00	3.00	10	10	10	41.8	41.8	41.8	32.733	0	46.377	149340000	66	NaN	NaN	NaN	NaN	NaN	20.65	20.93	21.44
173	H9JSY6	Lipase OS	0.811665534973145	1.76	0.22254316819518	20.88	20.80	100.00	4.00	20.07	21.02	100.00	4.00	22	22	22	56.4	56.4	56.4	46.569	0	112.55	355350000	106	20.29	21.31	20.05	21.86	16.10	20.09	22.12	21.96
174	H9JT91	heat shock protein 83	NaN	NaN	0	NaN	NaN	0.00	0.00	19.34	19.30	75.00	3.00	3	2	2	4.6	3.7	3.7	93.673	0	20.846	22246000	17	NaN	NaN	NaN	NaN	19.30	NaN	19.25	19.48
175	H9JTP8	PA2c domain-containing protein OS	-2.26846520105998	-4.82	0.970932962122098	22.73	22.68	100.00	4.00	25.00	24.93	75.00	3.00	6	6	6	52.3	52.3	52.3	15.272	0	78.74	437110000	69	20.46	22.79	22.58	25.10	NaN	24.93	24.47	25.60
176	H9JTT6	Apyrase	-4.47915395100912	-22.30	3.68559615007559	18.13	18.26	75.00	3.00	22.61	22.85	75.00	3.00	10	10	10	28.7	28.7	28.7	54.712	0	130.2	60620000	30	NaN	18.40	17.74	18.26	NaN	22.05	22.94	22.85
177	H9JTU0	Sulfatase domain-containing protein OS	-0.569838523864746	-1.48	0	NaN	NaN	25.00	1.00	18.14	18.14	50.00	2.00	4	4	4	15.9	15.9	15.9	56.564	0	8.8489	4491400	6	NaN	NaN	NaN	17.58	NaN	NaN	17.81	18.48
178	H9JTV2	nardilysin-like	NaN	NaN	0	NaN	NaN	0.00	0.00	NaN	NaN	25.00	1.00	4	4	3	14.9	14.9	10.8	58.899	0	12.409	14469000	8	NaN	NaN	NaN	NaN	NaN	NaN	17.99	NaN
179	H9JTY2	vanin-like protein 2 isoform X1	-2.14083337783813	-4.41	0.65676684403474	24.21	24.41	100.00	4.00	26.35	27.37	100.00	4.00	27	27	27	36	36	36	109.29	0	323.31	2711300000	208	22.33	25.49	23.34	25.69	22.48	26.71	28.03	28.18
180	H9JTZ4	glucose dehydrogenase [FAD, quinone]	1.43680206934611	2.71	1.03894757401136	20.42	20.31	100.00	4.00	18.99	18.60	75.00	3.00	43	43	34	44.9	44.9	36.2	139.1	0	323.31	668660000	208	19.56	19.94	20.69	21.51	NaN	18.28	20.08	18.60
181	H9JU99	Lipocln_cytosolic_FA-bd_dom domain-containing protein OS	-1.35509967803955	-2.56	0.81599860978481	18.46	18.46	50.00	2.00	19.82	19.82	50.00	2.00	5	5	5	45.4	45.4	45.4	10.961	0	11.809	28688000	10	NaN	19.04	NaN	17.88	NaN	NaN	19.97	19.66
182	H9JUB6	Glucosylceramidase OS	0.450394153594971	1.37	0.133571815603756	21.89	21.72	100.00	4.00	21.44	22.18	100.00	4.00	16	16	16	24.5	24.5	24.5	93.645	0	104.13	982850000	125	21.97	21.48	21.11	23.02	18.07	22.97	23.35	21.39
183	H9JUB7	Glucosylceramidase OS	-1.95549043019613	-3.88	1.33315771232562	19.78	19.86	100.00	4.00	21.73	21.95	75.00	3.00	8	8	8	30.7	30.7	30.7	48.511	0	84.24	104840000	59	19.37	20.35	18.38	21.01	NaN	21.04	21.95	22.21
184	H9JUB8	Lipocln_cytosolic_FA-bd_dom domain-containing protein OS	-3.17075061798096	-9.01	1.22206067703864	18.14	18.14	50.00	2.00	21.31	21.56	75.00	3.00	4	4	4	12.9	12.9	12.9	29.198	0	39.188	56267000	24	NaN	17.11	NaN	19.16	NaN	20.19	22.17	21.56
185	H9JUP8;Q1HPY8	Uncharacterized protein OS	1.47032356262207	2.77	1.35691402813677	27.42	27.55	100.00	4.00	25.95	26.10	100.00	4.00	13	13	13	76.5	76.5	76.5	20.256	0	301.41	5142300000	221	27.39	26.34	27.72	28.22	24.86	26.72	26.49	25.72
186	H9JUQ4	eIF1	0.836882591247559	1.79	0	NaN	NaN	25.00	1.00	18.73	18.73	50.00	2.00	3	3	3	23.2	23.2	23.2	20.304	0	7.3194	3956200	7	NaN	NaN	19.56	NaN	NaN	NaN	19.50	17.95
187	H9JUX8	Vitellogenin domain-containing protein OS	-0.267163276672363	-1.20	0.162585910941001	22.70	22.86	100.00	4.00	22.97	22.75	100.00	4.00	29	29	29	61.9	61.9	61.9	63.051	0	323.31	1279400000	215	22.70	23.41	21.65	23.03	22.17	22.12	23.34	24.23
188	H9JUX9	VWFD domain-containing protein OS	-0.544037342071533	-1.46	0.353527382396948	24.10	24.26	100.00	4.00	24.64	24.58	100.00	4.00	147	147	147	58.9	58.9	58.9	290.28	0	323.31	4247300000	911	24.02	25.06	22.81	24.50	24.12	23.68	25.03	25.73
189	H9JV86;H9JYG1;B9A9Z3;H9JY79	Histone H2A OS;Histone H2A OS;Histone H2A OS	0.960257053375244	1.95	1.05816010832019	22.99	23.10	100.00	4.00	22.03	21.72	100.00	4.00	3	3	3	19.8	19.8	19.8	13.616	0	6.3392	139650000	34	22.78	22.25	23.43	23.49	21.55	23.11	21.82	21.62
190	H9JVK2	C3 and PZP-like alpha-2-macroglobulin domain-containing protein 8 isoform X2	-1.83496348063151	-3.57	0	NaN	NaN	25.00	1.00	20.67	21.24	75.00	3.00	21	21	21	33.2	33.2	33.2	120.03	0	97.113	99101000	72	NaN	18.84	NaN	NaN	NaN	19.35	21.24	21.44
191	H9JW98	Carboxylic ester hydrolase OS	0.3542799949646	1.28	0.0824250303328534	23.66	23.78	100.00	4.00	23.30	24.23	100.00	4.00	23	23	23	44.4	44.4	44.4	63.296	0	323.31	2852000000	310	23.01	23.77	23.79	24.07	18.90	25.86	24.82	23.64
192	H9JWD5	SERPIN domain-containing protein OS	-2.13377793629964	-4.39	1.33931967350975	20.28	20.28	50.00	2.00	22.41	22.19	75.00	3.00	14	14	14	44.7	44.7	44.7	38.273	0	83.142	69562000	47	NaN	20.37	NaN	20.19	NaN	21.68	23.37	22.19
193	H9JWW0;H9JWW6;H9JWW7	Uncharacterized protein OS	-0.447034358978271	-1.36	0.108409565331625	21.05	20.94	100.00	4.00	21.50	22.41	100.00	4.00	13	13	4	51.2	51.2	14.1	31.155	0	91.51	206440000	74	21.18	20.70	20.23	22.11	17.23	22.35	22.47	23.95
194	H9JX50	Glyco_hydro_20 domain-containing protein OS	-2.07327238718669	-4.21	0.585783604602205	18.80	18.80	50.00	2.00	20.87	20.76	75.00	3.00	8	8	4	28.3	28.3	12.3	44.137	0	23.359	34647000	12	NaN	18.49	NaN	19.10	NaN	18.95	22.91	20.76
195	H9JX51	Beta-hexosaminidase OS	NaN	NaN	0	NaN	NaN	0.00	0.00	20.61	21.16	75.00	3.00	7	3	3	20	7.1	7.1	61.688	0	12.559	22449000	6	NaN	NaN	NaN	NaN	NaN	18.64	21.16	22.03
196	H9JX80;H9JX82	Uncharacterized protein OS	0.261373519897461	1.20	0.0733030422839765	24.55	24.37	100.00	4.00	24.29	25.29	100.00	4.00	15	15	9	35	35	22.6	58.704	0	119.27	967590000	138	24.33	24.41	24.04	25.44	20.65	24.76	25.93	25.82
197	H9JX83	glucosidase precursor	-0.826684633890789	-1.77	0.686336238518496	21.09	21.09	50.00	2.00	21.92	21.53	75.00	3.00	9	4	3	23	11.6	8.5	57.316	0	13.013	52300000	17	21.09	NaN	NaN	21.09	NaN	21.53	21.50	22.71
198	H9JXA6	microvitellogenin-like	NaN	NaN	0	NaN	NaN	0.00	0.00	NaN	NaN	25.00	1.00	7	5	5	26.8	19.3	19.3	34.733	0	13.453	40572000	12	NaN	NaN	NaN	NaN	20.85	NaN	NaN	NaN
199	H9JXB3	beta-glucosidase precursor	-2.34404230117798	-5.08	0.590194756916256	19.70	19.44	100.00	4.00	22.04	23.49	100.00	4.00	18	18	18	44.2	44.2	44.2	57.807	0	173.58	274180000	97	19.32	19.56	17.94	21.96	17.05	23.19	23.78	24.14
200	H9JXC7	Uncharacterized protein OS	-0.932931900024414	-1.91	0.37589593220414	21.53	21.57	100.00	4.00	22.46	21.99	100.00	4.00	16	16	16	38.3	38.3	38.3	48.903	0	132.29	330710000	69	21.44	21.70	20.72	22.24	20.57	21.34	22.64	25.28
201	H9JXF8	Yellow-f OS	-1.06989940007528	-2.10	0.613881945466284	20.23	20.08	100.00	4.00	21.30	22.03	75.00	3.00	12	12	12	36.6	36.6	36.6	49.77	0	61.309	121310000	36	19.70	20.46	19.70	21.08	NaN	19.62	22.03	22.26
202	H9JXK3	Uncharacterized protein OS	-1.18976084391276	-2.28	0.820631903726208	18.05	17.80	75.00	3.00	19.24	19.76	75.00	3.00	9	9	9	24.4	24.4	24.4	47.785	0	72.603	42640000	37	17.80	17.71	NaN	18.65	NaN	18.04	19.92	19.76
203	H9JXK4	E3 ubiquitin-protein ligase UBR3-like	-0.0658137003580741	-1.05	0.0358858732541905	19.69	19.36	75.00	3.00	19.76	19.36	75.00	3.00	9	9	9	26.7	26.7	26.7	53.589	0	212.51	92756000	38	19.19	20.53	NaN	19.36	NaN	19.36	19.25	20.67
204	H9JYA2	N-acetylneuraminate lyase	-4.38893445332845	-20.95	0	NaN	NaN	25.00	1.00	20.37	18.27	75.00	3.00	12	12	12	39.2	39.2	39.2	33.318	0	130.91	148880000	22	NaN	NaN	15.98	NaN	NaN	27.03	18.27	15.81
205	H9JYG4;H9JJE7	Histone H3 OS;Histone H3 OS	NaN	NaN	NaN	NaN	NaN	0.00	0.00	NaN	NaN	0.00	0.00	1	1	1	24.1	24.1	24.1	15.014	0	25.506	16431000	7	NaN	NaN	NaN	NaN	NaN	NaN	NaN	NaN
206	H9JYK0;H9JWW1	15-hydroxyprostaglandin dehydrogenase [D(+)]-like	NaN	NaN	NaN	NaN	NaN	0.00	0.00	NaN	NaN	0.00	0.00	12	3	3	51.2	14.1	14.1	31.231	0	11.076	5141500	6	NaN	NaN	NaN	NaN	NaN	NaN	NaN	NaN
207	O01953;H9JY10;H9JY13;H9JY12;H9JY11	Serine protease OS;Peptidase S1 domain-containing protein OS;Peptidase S1 domain-containing protein OS	-1.92844200134277	-3.81	0	NaN	NaN	25.00	1.00	NaN	NaN	25.00	1.00	3	3	3	15.5	15.5	15.5	29.721	0	40.349	6660500	4	NaN	NaN	NaN	17.47	NaN	NaN	19.40	NaN
208	P04148	Fibrohexamerin OS	NaN	NaN	NaN	NaN	NaN	0.00	0.00	NaN	NaN	0.00	0.00	9	1	1	47.7	5	5	25.166	0	12.045	138450000	16	NaN	NaN	NaN	NaN	NaN	NaN	NaN	NaN
209	P05790;H9J6G9	Fibroin heavy chain OS;Fibroin heavy chain OS	-2.00467300415039	-4.01	0.89163085640286	27.96	28.61	100.00	4.00	29.96	30.31	100.00	4.00	10	10	10	2.5	2.5	2.5	391.59	0	323.31	2845200000[illegible]	610	25.57	28.80	28.43	29.04	27.75	29.95	30.68	31.48
210	P07856	Sericin 1 OS	1.03095674514771	2.04	0.465097345914194	32.96	32.65	100.00	4.00	31.93	31.95	100.00	4.00	85	85	85	74	74	74	119.48	0	323.31	4822000000[illegible]	4088	35.47	31.06	33.38	31.93	32.56	31.26	32.05	31.85
211	P09334	Low molecular 30 kDa lipoprotein PBMHP-6 OS	-1.68995571136475	-3.23	1.67652094378521	17.99	17.99	50.00	2.00	19.68	19.68	50.00	2.00	2	2	2	9.4	9.4	9.4	29.734	0	6.2684	8507900	8	NaN	17.74	NaN	18.23	NaN	NaN	19.72	19.63
212	P09335	Low molecular 30 kDa lipoprotein PBMHP-12 OS	-1.2687832514445	-2.41	0.252486745042216	20.88	20.88	50.00	2.00	22.15	22.79	75.00	3.00	19	4	2	56.4	13.6	6.4	30.035	0	15.069	85574000	21	NaN	21.22	NaN	20.54	NaN	19.31	24.34	22.79
213	P09338;H9J4L7	Low molecular 30 kDa lipoprotein PBMHPC-23 OS	-2.79006735483805	-6.92	1.38490341973998	18.92	19.02	75.00	3.00	21.71	21.71	50.00	2.00	13	2	1	45.5	6.1	6.1	30.343	0	34.031	23591000	10	NaN	19.96	17.79	19.02	NaN	NaN	21.68	21.74
214	P10832	Chymotrypsin inhibitor SCI-II OS	NaN	NaN	0	NaN	NaN	0.00	0.00	NaN	NaN	25.00	1.00	3	3	2	56.5	56.5	17.7	7.067	0	7.328	35875000	10	NaN	NaN	NaN	NaN	NaN	NaN	20.49	NaN
215	P21828	Fibroin light chain OS	-1.6116795539856	-3.06	0.852102428500995	28.85	29.25	100.00	4.00	30.46	30.76	100.00	4.00	10	10	10	43.1	43.1	43.1	27.669	0	183.68	4596400000[illegible]	275	26.90	29.58	28.92	29.99	28.62	30.63	30.89	31.70
216	P48816	Lysozyme OS	-2.73579597473145	-6.66	0.340146123047982	17.62	17.62	50.00	2.00	20.36	20.36	50.00	2.00	6	6	6	38.7	38.7	38.7	15.668	0	83.612	27318000	8	NaN	17.99	NaN	17.26	NaN	NaN	23.33	17.39
217	P81902	Kazal-type serine proteinase inhibitor 1 (Spi1)	NaN	NaN	0	NaN	NaN	0.00	0.00	24.19	23.62	100.00	4.00	3	3	3	81.8	81.8	81.8	6.0267	0	9.957	1932500000	24	NaN	NaN	NaN	NaN	25.01	27.42	22.24	22.09
218	P84183;P04829;H9J7T4;H9J7T5	Actin, cytoplasmic A4 OS;Actin, cytoplasmic A3 OS;Uncharacterized protein OS;Uncharacterized protein OS	0.0868687629699707	1.06	0.150198497026344	25.52	25.47	100.00	4.00	25.43	25.47	100.00	4.00	19	19	5	65.2	65.2	24.2	41.821	0	237.1	1286900000	203	25.46	25.06	26.09	25.48	25.25	25.42	25.52	25.54
219	Q00801	Low molecular mass 30 kDa lipoprotein 21G1 OS	-2.67807769775391	-6.40	0	NaN	NaN	25.00	1.00	19.77	19.77	50.00	2.00	7	1	0	32.3	6.5	0	30.183	0	6.5018	6754000	10	NaN	NaN	NaN	17.09	NaN	NaN	19.63	19.91
220	Q008X1	Putative defense protein OS	NaN	NaN	0	NaN	NaN	0.00	0.00	18.30	16.46	75.00	3.00	2	2	2	15.2	15.2	15.2	18.482	0	11.469	15183000	4	NaN	NaN	NaN	NaN	NaN	15.94	22.49	16.46
221	Q1EPM0	Glyceraldehyde-3-phosphate dehydrogenase OS	-0.59333324432373	-1.51	0	NaN	NaN	25.00	1.00	20.77	20.77	50.00	2.00	4	4	4	16.3	16.3	16.3	35.428	0	10.572	32671000	16	NaN	20.17	NaN	NaN	NaN	NaN	20.43	21.10
222	Q1HPT1;H9JSP4;H9J314	ATP synthase subunit beta OS;ATP synthase subunit beta OS	-0.727214813232422	-1.66	0.272725290636007	19.85	19.45	100.00	4.00	20.57	20.15	100.00	4.00	17	17	17	49.4	49.4	49.4	54.987	0	55.428	43230000	30	19.44	19.26	21.24	19.46	19.16	21.14	23.15	18.84
223	Q1HPW2	Eukaryotic translation initiation factor 4A OS	0.82832145690918	1.78	0.617006027673439	20.18	20.18	50.00	2.00	19.36	19.36	50.00	2.00	2	2	2	6.2	6.2	6.2	47.54	0	4.3434	14843000	13	19.92	NaN	20.45	NaN	NaN	19.79	18.93	NaN
224	Q1HPY3	Secreted protein acidic and rich in cysteine OS	-0.150335311889648	-1.11	0	NaN	NaN	25.00	1.00	19.34	19.21	75.00	3.00	5	5	5	18.3	18.3	18.3	37.384	0	9.7306	6184800	7	NaN	NaN	NaN	19.19	NaN	19.21	18.57	20.24
225	Q27452	Phenoloxidase subunit 2 OS	NaN	NaN	0	NaN	NaN	0.00	0.00	19.73	19.73	50.00	2.00	7	7	7	11.4	11.4	11.4	80.118	0	94.652	5369300	8	NaN	NaN	NaN	NaN	NaN	NaN	20.22	19.24
226	Q2F5M1	GTP-binding nuclear protein OS	NaN	NaN	0	19.01	19.01	50.00	2.00	NaN	NaN	0.00	0.00	4	4	4	20.2	20.2	20.2	24.337	0	6.2176	7479100	12	18.99	NaN	19.02	NaN	NaN	NaN	NaN	NaN
227	Q2F5Q6	Kazal-type proteinase inhibitor OS	3.02129745483398	8.12	0	NaN	NaN	25.00	1.00	17.67	17.10	100.00	4.00	2	2	2	14.6	14.6	14.6	10.582	0	6.4983	8625100	10	NaN	NaN	20.70	NaN	16.24	16.45	20.26	17.74
228	Q2F5T3	ATP synthase subunit alpha OS	-0.94208812713623	-1.92	0	NaN	NaN	25.00	1.00	20.75	20.75	50.00	2.00	8	8	8	16.8	16.8	16.8	59.657	0	15.238	18728000	10	19.80	NaN	NaN	NaN	NaN	19.33	22.16	NaN
229	Q2F605	Ecdysteroid-regulated 16 kDa protein OS	-1.00486501057943	-2.01	0.401238178967606	22.13	21.75	75.00	3.00	23.13	22.60	75.00	3.00	5	5	5	36.6	36.6	36.6	15.824	0	57.375	288000000	51	NaN	20.96	21.75	23.67	NaN	22.28	24.50	22.60
230	Q2F622	Carboxylesterase OS	-1.86924076080322	-3.65	0.444678138617573	19.99	19.99	50.00	2.00	21.86	22.71	100.00	4.00	15	15	15	40.6	40.6	40.6	61.415	0	323.31	145080000	75	NaN	19.49	NaN	20.49	18.41	22.21	23.61	23.20
231	Q2F637;H9IZF9	14-3-3 protein zeta OS;14_3_3 domain-containing protein OS	-1.5394458770752	-2.91	1.02457496583025	18.16	18.28	75.00	3.00	19.70	19.92	75.00	3.00	3	3	2	12.6	12.6	8.5	28.096	0	11.998	14185000	10	NaN	18.28	18.59	17.61	NaN	18.49	20.69	19.92
232	Q3LB95	Chemosensory protein 7;Chemosensory protein11 OS	-0.294469833374023	-1.23	0	18.21	18.21	50.00	2.00	NaN	NaN	25.00	1.00	2	2	2	27	27	27	13.519	0	4.7515	2311800	2	NaN	NaN	18.48	17.94	NaN	NaN	NaN	18.51
233	Q402D9	Juvenile hormone binding protein OS	0.679739475250244	1.60	0.281446421628137	20.76	20.91	100.00	4.00	20.08	20.09	75.00	3.00	11	11	11	63	63	63	27.294	0	40.144	92272000	64	21.52	20.29	19.56	21.67	18.45	NaN	20.09	21.71
234	Q5R1P5	Heat shock protein hsp21.4 OS	-0.557862281799316	-1.47	0.262527785966692	17.36	17.36	50.00	2.00	17.91	17.91	50.00	2.00	2	2	2	17.1	17.1	17.1	21.404	0	34.895	4802800	4	NaN	NaN	17.65	17.06	NaN	NaN	17.20	18.63
235	Q5UAU1	60S acidic ribosomal protein P0 OS	NaN	NaN	0	NaN	NaN	0.00	0.00	17.51	17.51	50.00	2.00	2	2	2	6.6	6.6	6.6	34.189	0	6.0835	1048900	3	NaN	NaN	NaN	NaN	NaN	NaN	17.65	17.37
236	Q6Q2D6	Serpin-4A OS	-0.714735507965088	-1.64	0.213276150705238	20.10	20.34	75.00	3.00	20.81	21.38	100.00	4.00	10	10	10	31	31	31	46.3	0	124.56	125010000	54	NaN	20.58	19.38	20.34	17.97	20.25	22.52	22.50
237	Q6T3A7	Thiol peroxiredoxin OS	0.15923023223877	1.12	0.120025298115833	21.60	21.61	100.00	4.00	21.44	21.33	100.00	4.00	4	4	4	20.5	20.5	20.5	21.916	0	10.906	104370000	28	21.41	21.81	22.10	21.09	22.60	21.58	20.51	21.09
238	Q6T9Z7	Fibroinase OS	NaN	NaN	0	NaN	NaN	0.00	0.00	19.67	19.67	50.00	2.00	3	3	3	11.4	11.4	11.4	38.138	0	146.49	5822600	6	NaN	NaN	NaN	NaN	NaN	NaN	20.47	18.88
239	Q7Z1E6;H9IT95	Calreticulin OS;Calreticulin OS	-1.05085627237956	-2.07	0.825093754609826	18.91	18.95	75.00	3.00	19.96	20.31	100.00	4.00	3	3	3	13.3	13.3	13.3	45.801	0	124.09	19603000	17	18.95	NaN	18.35	19.43	18.59	20.62	20.07	20.56
240	Q8MMK7	Chemosensory protein 4 OS	-0.513985633850098	-1.43	0.43555287758053	19.14	19.14	50.00	2.00	19.65	19.49	75.00	3.00	5	5	5	44.9	44.9	44.9	14.549	0	15.64	9056700	11	NaN	NaN	19.19	19.08	NaN	19.10	19.49	20.36
241	Q8N0P2;H9J523	Heat shock cognate protein OS	0.696887493133545	1.62	1.20263489888618	20.43	20.41	100.00	4.00	19.73	19.64	100.00	4.00	7	4	1	13.1	7.1	2.8	71.175	0	10.627	33327000	35	19.98	20.11	20.72	20.91	19.34	20.30	19.57	19.71
242	Q8T113	27 kDa glycoprotein OS	-2.40390205383301	-5.29	0	NaN	NaN	25.00	1.00	20.77	20.77	50.00	2.00	6	6	6	36.1	36.1	36.1	24.902	0	36.151	11582000	10	NaN	18.37	NaN	NaN	NaN	NaN	20.72	20.82
243	Q8T8B2	Tubulin beta chain OS	0.291454792022705	1.22	0.383208179910728	21.82	21.85	100.00	4.00	21.53	21.58	100.00	4.00	11	11	0	41.2	41.2	0	50.214	0	214.89	126440000	116	21.69	21.35	22.23	22.02	20.85	21.76	22.11	21.39
244	Q9BLC5	Heat shock protein 83 OS	0.820107460021973	1.77	1.33997643752739	21.58	21.59	100.00	4.00	20.76	20.56	100.00	4.00	5	5	4	9.1	9.1	8.1	82.421	0	30.228	67740000	33	21.55	21.00	22.13	21.63	20.58	21.44	20.46	20.54
245	Q9BPS3	Elongation factor 1 gamma OS	NaN	NaN	0	NaN	NaN	0.00	0.00	18.53	18.53	50.00	2.00	2	2	2	6.1	6.1	6.1	48.387	0	3.4987	2168900	4	NaN	NaN	NaN	NaN	NaN	NaN	19.13	17.94
246	Q9GSB6;Q5R1P7	Heat shock protein hsp20.4 OS;Heat shock protein hsp 19.9 OS	NaN	NaN	0	NaN	NaN	0.00	0.00	NaN	NaN	25.00	1.00	2	2	2	13.3	13.3	13.3	20.427	0	9.7066	4810600	5	NaN	NaN	NaN	NaN	NaN	NaN	19.95	NaN
