# Supplementary material for: The Role of Filippi’s Glands in the Silk Moths Cocoon Construction
Source: Int J Mol Sci. 2021 Dec 16;22(24):13523. doi: 10.3390/ijms222413523 (PMC8708004; doi:10.3390/ijms222413523)
Supplement: Supplementary file 1 [file ijms-22-13523-s001.zip › Table S2.pdf]

Table S2. A list of candidate proteins underrepresented in both the FG -enriched transcriptome [13] and the silk of FG-depleted larvae. The red/blue scale shows up/down regulation in the previously published transcriptome [13].

	Uniprot ID	SilkDB 3.0 ID	Fasta headers	Wang log2FC (FG/ctr)	Student's T-test Difference FG-_FG+	Fold change	"-Log Student's T-test p-value FG-_FG+"	Intensity FG- (1)	Intensity FG- (2)	Intensity FG- (3)	Intensity FG- (4)	Intensity FG+ (1)	Intensity FG+ (2)	Intensity FG+ (3)	Intensity FG+ (4)
1	**H9IWH6**	**BGIBMGA001608**	**alpha-1,6-mannosyl-glycoprotein 2-beta-N-acetylglucosaminyltransferase isoform X1**	2.4349	NaN	NaN	0.0000	**NaN**	**NaN**	**NaN**	**NaN**	NaN	NaN	18.71	20.27
2	**H9IZK4**	**BGIBMGA002689**	**seroin 1 precursor**	4.1651	0.773374557	1.71	0.5183	**29.09**	**28.77**	**29.96**	**31.19**	28.06	29.42	29.92	28.51
3	**H9J2B8**	**BGIBMGA003655**	**Death domain-containing protein OS**	1.8199	-0.929911613	-1.91	0.1184	**NaN**	**18.73**	**NaN**	**19.26**	NaN	16.56	19.25	23.96
4	**H9J452;H9J453;H9J454**	**BGIBMGA004291**	**Uncharacterized protein OS;Uncharacterized protein OS;Uncharacterized protein OS**	-1.7988	-1.649058342	**-3.14**	0	**NaN**	**19.07**	**NaN**	**NaN**	NaN	NaN	23.03	18.42
5	**H9J5L6**	**BGIBMGA004806**	**Uncharacterized protein OS**	-1.7724	-4.910666943	**-30.08**	0	**NaN**	**15.51**	**NaN**	**NaN**	NaN	NaN	25.30	15.53
6	**H9J709**	**BGIBMGA005301**	**Uncharacterized protein OS**	3.6578	-0.861912727	-1.82	0.2016	**19.86**	**19.65**	**21.95**	**22.71**	17.37	23.30	23.40	23.55
7	**H9J7K8**	**BGIBMGA005500**	**Beta-glucuronidase OS**	2.6387	-0.217092037	-1.16	0.0899	**17.64**	**18.88**	**18.49**	**20.45**	NaN	19.07	20.17	18.01
8	**H9J8H0**	**BGIBMGA005812**	**Arginine kinase**	1.4863	-2.390730858	**-5.24**	0.0000	**NaN**	**18.86**	**NaN**	**NaN**	NaN	NaN	20.35	22.16
9	**H9J9P6**	**BGIBMGA006239**	**Uncharacterized protein OS**	1.7783	NaN	NaN	0.0000	**NaN**	**NaN**	**NaN**	**NaN**	NaN	19.40	20.40	19.04
10	**H9JBW7**	**BGIBMGA007012**	**Extracellular serine/threonine protein kinase**	2.0489	NaN	NaN	0.0000	**NaN**	**NaN**	**NaN**	**NaN**	NaN	21.39	22.23	21.89
11	**H9JC27**	**BGIBMGA007072**	**yellow-b**	2.471	-0.695773443	-1.62	0.2499	**NaN**	**20.43**	**NaN**	**21.46**	NaN	20.37	21.51	23.04
12	**H9JET3**	**BGIBMGA008030**	**Allantoate amidinohydrolase**	4.3665	NaN	NaN	0.0000	**NaN**	**NaN**	**NaN**	**NaN**	NaN	NaN	19.15	19.49
13	**H9JI83**	**BGIBMGA009232**	**Aldose 1-epimerase OS**	1.6745	NaN	NaN	0.0000	**NaN**	**NaN**	**NaN**	**NaN**	NaN	NaN	16.97	19.41
14	**H9JIY7**	**BGIBMGA009488**	**venom acid phosphatase Acph-1-like**	2.2405	-0.486234983	-1.40	0.1042	**NaN**	**20.86**	**17.54**	**19.79**	16.34	19.93	21.14	22.11
15	**H9JJU8**	**BGIBMGA009799**	**Aldo_ket_red domain-containing protein OS**	1.3529	-0.520703634	-1.43	0.0838	**NaN**	**23.65**	**18.06**	**19.73**	NaN	18.52	20.95	23.53
16	**H9JL76**	**BGIBMGA010277**	**venom acid phosphatase Acph-1-like**	**4.1436**	NaN	NaN	0.0000	**NaN**	**NaN**	**NaN**	**NaN**	NaN	18.41	17.89	21.86
17	**H9JLC2**	**BGIBMGA010323**	**Uncharacterized protein OS**	1.6572	-1.594560623	**-3.02**	0.0000	**NaN**	**18.16**	**NaN**	**NaN**	NaN	19.42	19.78	20.07
18	**H9JN76**	**BGIBMGA010979**	**Uncharacterized protein OS**	-1.7055	-1.804402987	**-3.49**	0.8643	**18.47**	**18.30**	**19.10**	**19.84**	NaN	20.71	22.67	18.82
19	**H9JPD2**	**BGIBMGA011386**	**Uncharacterized protein OS**	1.1303	NaN	NaN	0.0000	**NaN**	**NaN**	**NaN**	**NaN**	NaN	19.35	18.48	19.96
20	**H9JQ96**	**BGIBMGA011702**	**EN protein binding/engrailed nuclear homeoprotein-regulated protein**	1.62	NaN	NaN	0.0000	**NaN**	**NaN**	**NaN**	**NaN**	NaN	19.67	19.16	20.83
21	**H9JXF8**	**BGIBMGA014224**	**Yellow-f OS**	1.1647	-1.0698994	**-2.10**	0.6139	**19.70**	**20.46**	**19.70**	**21.08**	NaN	19.62	22.03	22.26
