# Supplementary material for: The Role of Filippi’s Glands in the Silk Moths Cocoon Construction
Source: Int J Mol Sci. 2021 Dec 16;22(24):13523. doi: 10.3390/ijms222413523 (PMC8708004; doi:10.3390/ijms222413523)
Supplement: Supplementary file 1 [file ijms-22-13523-s001.zip › Table S3.pdf]

Table S3. Phospho output file (STY) from MaxQuant was processed using Perseus 1.5.2.4 software. The significance of the differences was evaluated in Perseus using the false discovery rates (q-values). The N-terminus of FibH is phosphorylated at serine 51 in control cocoons but not in cocoons of ablated larvae. Another phosphoserine (S90-ph) of FibH (Fig. 7B) showed a similar trend.

	Samples data binary logarithm								Quantification and statictics				
	Intensity FG- (1)	Intensity FG- (2)	Intensity FG- (3)	Intensity FG- (4)	Intensity FG+ (1)	Intensity FG+ (2)	Intensity FG+ (3)	Intensity FG+ (4)	Student's T-test Significant a_c	Localization probability	PEP	Score	Delta score
1	NaN	18.6538	17.6932	18.1664	NaN	22.0398	23.3313	23.8275	+	1	1.71E-21	196.37	153.65
2	17.6985	21.643	17.8986	19.6185	NaN	NaN	NaN	22.7505		0.999572	0.0326194	47.64	13.363
3	NaN	NaN	NaN	NaN	NaN	NaN	NaN	22.2256		0.997366	4.79E-13	162.41	96.14
4	NaN	NaN	NaN	NaN	NaN	18.4773	20.3205	21.2671		1	0.00377339	112.12	85.095
5	20.4598	17.9279	15.2623	NaN	NaN	NaN	NaN	21.8112		1	0.0386499	44.692	6.9646
6	NaN	NaN	NaN	NaN	NaN	17.2364	17.8046	21.5604		0.847895	0.000987512	69.435	50.06
7	17.9806	20.64	19.156	19.3515	NaN	NaN	18.6514	NaN		0.873744	0.0140889	81.191	51.115
8	NaN	NaN	15.9046	NaN	NaN	NaN	NaN	NaN		1	0.0336193	53.968	15.005
9	NaN	NaN	18.3017	NaN	NaN	17.3359	18.2064	20.5484		1	0.0348727	41.54	13.678
10	NaN	NaN	NaN	NaN	NaN	NaN	NaN	17.632		0.998154	0.0143858	94.61	56.817
11	18.1778	NaN	NaN	NaN	16.4844	NaN	16.6774	15.7287		1	0.0176527	132.56	17.801
12	NaN	NaN	NaN	NaN	18.9797	17.0901	NaN	21.3146		0.97565	9.16E-05	133.16	94.793
13	NaN	NaN	NaN	NaN	NaN	NaN	19.2233	17.1434		0.995014	3.02E-14	158.65	83.238
14	NaN	NaN	16.6271	NaN	NaN	NaN	NaN	17.6262		1	0.0313198	65.232	11.457
15	18.1781	NaN	NaN	NaN	NaN	NaN	NaN	NaN		1	0.0305949	79.089	37.721
16	23.1377	20.5954	15.6777	19.9065	26.1073	NaN	NaN	NaN		1	0.00409802	120.65	35.339
17	17.1329	NaN	16.009	16.03	NaN	NaN	15.6484	NaN		1	0.0292939	68.485	51.017
18	NaN	NaN	NaN	19.2903	NaN	18.4973	20.1267	NaN		0.88845	0.037191	60.799	9.7395
19	NaN	NaN	18.0111	NaN	18.8635	NaN	17.1056	NaN		1	0.0341035	45.257	10.572
20	NaN	15.4556	NaN	NaN	NaN	NaN	15.6934	16.0928		1	0.037652	44.98	12.23
21	17.6662	18.0967	16.1488	NaN	NaN	NaN	15.8863	NaN		0.988229	0.0303729	49.423	34.749
22	NaN	NaN	NaN	NaN	NaN	18.6542	NaN	20.9787		0.775368	0.0126032	93.243	48.931
23	NaN	NaN	NaN	NaN	24.0071	NaN	NaN	21.3959		1	0.0147202	112.11	48.413
24	NaN	NaN	16.9944	NaN	16.9335	NaN	16.4609	19.7367		0.999901	0.0340124	74.133	19.262
25	NaN	18.3222	NaN	NaN	NaN	18.1811	18.6874	24.7404		0.832681	0.0354279	55.531	23.21
26	NaN	NaN	NaN	17.1788	NaN	18.2967	19.1148	18.5702		1	0.0343357	54.722	10.953
27	NaN	NaN	NaN	NaN	26.8409	NaN	15.776	22.4682		0.992593	0.0330213	66.299	15.595
28	NaN	NaN	NaN	17.4451	NaN	NaN	NaN	18.397		0.792748	0.0306798	63.727	23.034
29	NaN	NaN	NaN	NaN	NaN	18.6358	19.0123	19.9493		1	0.00902382	126.52	62.257
30	NaN	NaN	NaN	NaN	19.9413	NaN	NaN	NaN		1	0.0175354	55.546	12.83
31	NaN	NaN	NaN	NaN	19.9413	NaN	NaN	NaN		1	0.0175354	55.546	12.83
32	19.2867	22.2752	20.9756	20.5578	25.5031	NaN	NaN	NaN		1	0.0220756	97.565	23.724
33	19.2867	22.2752	20.9756	20.5578	25.5031	NaN	NaN	NaN		1	0.0220756	97.565	23.724
34	17.6216	18.8049	17.5289	19.2162	18.2479	NaN	NaN	NaN		0.995139	0.0343947	40.237	19.298
35	17.3279	NaN	NaN	15.2175	NaN	NaN	NaN	NaN		1	0.0142823	74.962	36.301
36	17.3279	NaN	NaN	15.2175	NaN	NaN	NaN	NaN		0.896204	0.0142823	74.962	36.301
37	20.5828	NaN	18.6067	19.1967	NaN	NaN	NaN	19.68		0.978857	0.0360995	40.515	7.5336
38	NaN	NaN	NaN	16.7028	NaN	NaN	17.8422	NaN		0.99997	0.0302089	48.405	16.313
39	NaN	NaN	16.7081	NaN	NaN	NaN	17.8676	NaN		0.772879	0.0373195	42.708	14.676
40	NaN	NaN	18.5801	NaN	17.1859	15.9698	18.6069	NaN		0.981122	0.0309677	75.294	22.293
41	NaN	NaN	NaN	NaN	22.9669	20.5575	NaN	22.0006		0.936486	0.0327734	64.52	13.354
42	NaN	NaN	NaN	NaN	24.6042	NaN	22.3784	24.3616		0.756586	0.0258518	66.606	6.9877
43	16.1568	NaN	16.8608	NaN	NaN	NaN	NaN	NaN		0.997627	0.0217703	63.076	18.462
44	NaN	NaN	NaN	NaN	NaN	21.4371	23.6502	24.6123		0.974943	0.0203689	90.149	11.079
45	22.2453	20.8034	18.145	NaN	NaN	NaN	NaN	NaN		0.780334	0.0064088	53.867	30.958
46	NaN	NaN	NaN	NaN	17.5768	NaN	16.5609	20.6096		1	0.0362308	59.57	17.474
47	NaN	19.2975	NaN	NaN	NaN	21.0469	21.8278	22.886		1	0.035354	46.592	21.084
48	NaN	NaN	NaN	NaN	23.4079	20.7776	20.8706	NaN		0.999958	0.0207691	56.036	33.391
49	NaN	NaN	NaN	NaN	23.4079	20.7776	20.8706	NaN		0.999951	0.0207691	56.036	33.391
50	NaN	NaN	NaN	NaN	18.9528	20.6712	16.6208	NaN		0.999969	0.0324723	71.879	8.4705
51	NaN	NaN	NaN	NaN	18.9528	20.6712	16.6208	NaN		0.999867	0.0324723	71.879	8.4705
52	NaN	NaN	NaN	NaN	NaN	14.8379	16.6229	17.7626		1	0.0188278	80.688	9.2377
53	17.6216	18.8049	17.5289	19.2162	18.2479	NaN	NaN	NaN		0.946847	0.0343947	40.237	19.298
54	NaN	NaN	NaN	NaN	NaN	15.4903	NaN	17.0188		0.809671	0.0188041	61.765	11.731
55	NaN	NaN	NaN	NaN	NaN	15.4903	NaN	17.0188		0.809671	0.0188041	61.765	11.731
56	20.5828	NaN	18.6067	19.1967	NaN	NaN	NaN	19.68		0.978202	0.0360995	40.515	7.5336
57	NaN	NaN	NaN	27.3596	24.0325	22.7162	22.3961	22.5392		1	0.0268203	76.98	19.934
58	NaN	NaN	NaN	27.3596	24.0325	22.7162	22.3961	22.5392		1	0.0268203	76.98	19.934
59	22.8974	NaN	NaN	NaN	NaN	19.529	NaN	21.584		0.947131	0.0387153	48.255	10.036
60	NaN	19.7121	NaN	NaN	20.1014	NaN	NaN	NaN		1	0.0183186	84.743	12.866
61	NaN	NaN	NaN	NaN	24.6042	NaN	22.3784	24.3616		0.801047	0.0258518	66.606	6.9877
62	NaN	NaN	NaN	NaN	17.16	15.3646	18.0909	16.8966		0.752327	0.0200957	55.815	16.9
63	NaN	NaN	NaN	NaN	NaN	21.4371	23.6502	24.6123		0.974943	0.0203689	90.149	11.079
64	NaN	NaN	NaN	NaN	NaN	14.8379	16.6229	17.7626		1	0.0188278	80.688	9.2377
65	NaN	NaN	NaN	16.9795	NaN	NaN	16.5531	NaN		0.998722	0.0271183	71.976	14.566
66	NaN	19.7121	NaN	NaN	20.1014	NaN	NaN	NaN		1	0.0183186	84.743	12.866
67	NaN	NaN	NaN	NaN	17.16	15.3646	18.0909	16.8966		0.866847	0.0200957	55.815	16.9
68	22.4859	22.3499	21.0935	NaN	21.0168	NaN	NaN	NaN		0.944483	0.0341582	46.353	21.521
69	NaN	NaN	NaN	NaN	17.5768	NaN	16.5609	20.6096		1	0.0362308	59.57	17.474
70	NaN	19.2975	NaN	NaN	NaN	21.0469	21.8278	22.886		1	0.035354	46.592	21.084
71	20.6424	20.2754	NaN	19.594	NaN	19.9219	NaN	NaN		0.792751	0.0365658	49.34	26.688
72	20.6424	20.2754	NaN	19.594	NaN	19.9219	NaN	NaN		0.792751	0.0365658	49.34	26.688
73	20.6424	20.2754	NaN	19.594	NaN	19.9219	NaN	NaN		0.931037	0.0365658	49.34	26.688
74	20.6424	20.2754	NaN	19.594	NaN	19.9219	NaN	NaN		0.930829	0.0365658	49.34	26.688
75	NaN	NaN	NaN	20.0944	NaN	NaN	NaN	NaN		0.796255	0.0372064	41.366	21.323
76	16.7678	NaN	NaN	16.3682	NaN	NaN	17.3535	NaN		0.99331	0.0256842	55.841	22.637
77	NaN	NaN	19.5525	NaN	NaN	NaN	17.9073	18.9283		0.999936	0.0323137	51.939	13.14
78	NaN	NaN	19.5525	NaN	NaN	NaN	17.9073	18.9283		0.999936	0.0323137	51.939	13.14
79	23.4641	24.1115	23.3426	NaN	NaN	NaN	NaN	23.2251		0.991572	0.0347707	59.983	20.336
80	23.4641	24.1115	23.3426	NaN	NaN	NaN	NaN	23.2251		0.996838	0.0347707	59.983	20.336
81	17.8994	NaN	NaN	NaN	NaN	18.4223	NaN	NaN		0.876681	0.0257592	51.211	18.916
82	22.2957	NaN	18.8074	18.5792	NaN	NaN	NaN	20.8823		0.863782	0.0263281	48.354	15.5
83	19.6328	NaN	NaN	NaN	18.8374	NaN	18.9934	15.9189		1	0.0371185	45.873	23.751
84	NaN	18.2952	17.1833	16.4748	NaN	NaN	NaN	NaN		1	0.0380963	44.254	13.218
85	16.5005	NaN	NaN	NaN	NaN	NaN	NaN	NaN		0.999867	0.0240655	55.806	10.471
86	28.3737	NaN	17.9578	25.8594	NaN	24.4441	NaN	NaN		0.812369	0.00226256	77.357	33.546
87	NaN	NaN	NaN	NaN	24.9295	NaN	22.7952	24.9264		0.963978	0.00603154	66.702	27.946
88	NaN	NaN	NaN	18.7595	NaN	NaN	NaN	NaN		1	0.0283319	87.083	17.205
89	NaN	NaN	NaN	18.7595	NaN	NaN	NaN	NaN		1	0.0283319	87.083	17.205
90	18.9716	20.8037	20.6914	20.4392	NaN	NaN	NaN	NaN		0.828973	0.0374932	59.792	34.173
91	18.9716	20.8037	20.6914	20.4392	NaN	NaN	NaN	NaN		0.791803	0.0374932	59.792	34.173
92	NaN	NaN	NaN	NaN	NaN	NaN	NaN	22.4819		0.997375	0.0225831	57.578	25.028
93	NaN	NaN	NaN	NaN	NaN	NaN	NaN	22.4819		0.997574	0.0225831	57.578	25.028
94	20.3324	18.2397	NaN	17.8032	NaN	NaN	NaN	18.3703		0.999177	0.0248999	55.337	14.669
95	20.3324	18.2397	NaN	17.8032	NaN	NaN	NaN	18.3703		0.997698	0.0248999	55.337	14.669
96	NaN	NaN	NaN	NaN	24.9406	20.1743	19.0572	19.7723		0.966801	0.0297351	45.357	16.33
97	NaN	NaN	NaN	NaN	24.9406	20.1743	19.0572	19.7723		0.971858	0.0297351	45.357	16.33
98	NaN	NaN	NaN	NaN	24.9406	20.1743	19.0572	19.7723		0.910053	0.0297351	45.357	16.33
99	NaN	NaN	NaN	20.0944	NaN	NaN	NaN	NaN		0.774498	0.0372064	41.366	21.323
100	NaN	NaN	NaN	NaN	NaN	18.4414	20.4748	22.749		0.990451	0.0355472	52.853	23.313
101	16.7678	NaN	NaN	16.3682	NaN	NaN	17.3535	NaN		0.854582	0.0256842	55.841	22.637
102	16.8172	NaN	NaN	NaN	16.6611	NaN	NaN	16.6814		0.99785	0.0195491	42.708	24.499
103	16.8172	NaN	NaN	NaN	16.6611	NaN	NaN	16.6814		0.999916	0.0195491	42.708	24.499
104	17.8994	NaN	NaN	NaN	NaN	18.4223	NaN	NaN		0.999948	0.0257592	51.211	18.916
105	17.8994	NaN	NaN	NaN	NaN	18.4223	NaN	NaN		0.980918	0.0257592	51.211	18.916
106	22.2957	NaN	18.8074	18.5792	NaN	NaN	NaN	20.8823		0.883364	0.0263281	48.354	15.5
107	20.7399	NaN	17.4183	NaN	NaN	NaN	17.5696	NaN		0.99994	0.0184057	42.618	24.54
108	20.7399	NaN	17.4183	NaN	NaN	NaN	17.5696	NaN		0.999941	0.0184057	42.618	24.54
109	19.6328	NaN	NaN	NaN	18.8374	NaN	18.9934	15.9189		1	0.0371185	45.873	23.751
110	NaN	18.2952	17.1833	16.4748	NaN	NaN	NaN	NaN		1	0.0380963	44.254	13.218
111	NaN	NaN	23.9445	NaN	22.4129	NaN	NaN	NaN		0.969473	0.0358386	40.428	14.708
112	17.0675	NaN	NaN	15.9828	NaN	NaN	NaN	16.0384		0.950354	0.0367518	53.403	16.391
113	17.0675	NaN	NaN	15.9828	NaN	NaN	NaN	16.0384		0.763731	0.0367518	53.403	16.391
114	16.5005	NaN	NaN	NaN	NaN	NaN	NaN	NaN		0.999981	0.0240655	55.806	10.471
115	NaN	NaN	17.039	NaN	NaN	16.7324	19.1349	18.7859		0.826547	0.0238301	50.505	19.845
116	NaN	NaN	18.1186	NaN	21.0697	18.4453	NaN	19.3811		1	0.00816905	80.165	26.16
117	NaN	NaN	18.1186	NaN	21.0697	18.4453	NaN	19.3811		1	0.00816905	80.165	26.16
118	23.8724	20.4661	22.8989	20.5989	NaN	20.062	NaN	NaN		0.998127	0.0373914	49.106	14.831
119	23.8724	20.4661	22.8989	20.5989	NaN	20.062	NaN	NaN		0.999917	0.0373914	49.106	14.831
120	NaN	NaN	NaN	18.7595	NaN	NaN	NaN	NaN		1	0.0283319	87.083	17.205
121	18.9716	20.8037	20.6914	20.4392	NaN	NaN	NaN	NaN		0.927784	0.0374932	59.792	34.173
122	17.6862	NaN	NaN	NaN	NaN	NaN	NaN	NaN		0.963459	0.0384217	40.767	6.3234
123	17.6862	NaN	NaN	NaN	NaN	NaN	NaN	NaN		0.960556	0.0384217	40.767	6.3234
124	20.3324	18.2397	NaN	17.8032	NaN	NaN	NaN	18.3703		0.99713	0.0248999	55.337	14.669
125	NaN	NaN	NaN	20.0944	NaN	NaN	NaN	NaN		0.778963	0.0372064	41.366	21.323
126	20.7399	NaN	17.4183	NaN	NaN	NaN	17.5696	NaN		0.800618	0.0184057	42.618	24.54
127	20.7399	NaN	17.4183	NaN	NaN	NaN	17.5696	NaN		0.800618	0.0184057	42.618	24.54

	Quantification and statictics (continued)								Protein identifiers	
	Score for localization	Mass error [ppm]	Intensity	Position	"-Log Student's T-test p-value Ab/Ctr"	Student's T-test q-value Ab/Ctr	Student's T-test Difference Ab/Ctr	Student's T-test Test statistic Ab/Ctr	Protein Identifiers	Protein (Uniprot Id)
1	196.37	1.5116	37327000	51	2.90874	0.068	-4.89506	-6.98633	51	P05790
2	47.64	-0.23616	67820000	131	0	0.943445	-3.53584	0	131	H9IWL7
3	162.41	0.5959	7327700	47	0	0.941026	NaN	0	47	H9IZK4
4	112.12	-0.65278	4374500	700	0	0.938619	NaN	0	700	H9J1H2
5	44.692	-0.87997	6247200	5	0	0.936235	-3.92787	0	5	H9J3A1
6	69.435	1.569	10304000	339	0	0.933852	NaN	0	339	H9J6C7
7	81.191	-1.7034	5251100	6	0	0.931482	0.630581	0	6	H9JD84
8	53.968	-1.3511	2652600	8	0	0.929124	NaN	0	8;8	H9JK09
9	41.54	2.2537	2972400	7	0	0.926778	-0.395244	0	7	H9JX42
10	94.61	-0.62894	9358900	226	0	0.924443	NaN	0	226	H9JY24
11	132.56	2.2652	1296500	150	0	0.922121	1.88098	0	150	H9JYE6
12	93.649	0.239	12681000	90	0	0.899578	NaN	0	90	P05790
13	158.65	0.45019	82966000	41	0	0.91986	NaN	0	41	P07856
14	65.232	-1.7941	48911000	24	0	0.91756	-0.999073	0	24	H9ITS5
15	79.089	-1.0412	2396600	409	0	0.915272	NaN	0	409	H9J107
16	120.65	3.0538	96640000	7	0	0.912995	-6.27792	0	7	H9J557
17	68.485	-1.4327	1230600	5	0	0.91073	0.742169	0	5	H9J8H0
18	60.799	-0.013739	4151000	4	0	0.908475	-0.0217161	0	4	H9JPB9
19	45.257	-1.0659	2805500	7	0	0.906232	0.0265312	0	7	H9JW59
20	44.98	1.4373	471370	11	0	0.90401	-0.43751	0	11	H9JXV6
21	49.423	2.4616	1176800	5	0	0.901789	1.41763	0	5	Q6PUF7
22	93.243	-4.2201	12965000	420	0	0.945876	NaN	0	420	H9ITP2
23	106.68	-2.4846	34236000	209	0	0.94832	NaN	0	209	H9J4N6
24	74.133	0.66671	1671000	251	0	0.999924	-0.716022	0	251	H9JB08
25	55.531	-3.2313	29935000	238	0	0.997207	-2.21403	0	238	H9JKP7
26	54.722	-3.2459	1427300	240	0	0.994504	-1.48169	0	240	H9JRB4
27	66.299	-1.3661	155510000	305	0	0.991838	NaN	0	305	H9JRI1
28	63.727	-2.3851	3636800	2	0	0.989175	-0.951952	0	2	H9JRS0
29	116.73	2.7315	8033800	788	0	0.986516	NaN	0	788	H9JRX3
30	55.546	1.9091	1622300	848	0	0.983893	NaN	0	848	E9JEG8
31	55.546	1.9091	1622300	851	0	0.981262	NaN	0	851	E9JEG8
32	97.565	-1.521	67363000	14	0	0.978645	-4.72926	0	14	H9IW69
33	97.565	-1.521	67363000	16	0	0.976043	-4.72926	0	16	H9IW69
34	40.237	-1.5694	3348500	107	0	0.973454	0.0449429	0	107	H9IWR1
35	74.962	-1.5097	5405000	652	0	0.970878	NaN	0	652	H9IX11
36	59.57	-1.4743	5405000	655	0	0.968317	NaN	0	655	H9IX11
37	40.515	-0.79655	10135000	10	0	0.965768	-0.217958	0	10	H9IYB6
38	48.405	-2.712	1111800	879	0	0.963234	-1.13938	0	879	H9J478
39	42.708	-0.55771	1894600	3	0	0.960712	-1.1595	0	3	H9JAT8
40	68.893	1.8778	5601300	1780	0	0.958204	1.32588	0	1780	H9JB68
41	57.794	-3.0534	117830000	741	0	0.955708	NaN	0	741	H9JBC1
42	65.423	4.0284	327600000	628	0	0.953226	NaN	0	628	H9JGB3
43	63.076	-3.7098	992880	293	0	0.950756	NaN	0	293	H9JI20
44	90.149	0.036696	41651000	60	0	0.897399	NaN	0	60	H9JLH4
45	53.867	-2.8593	7091400	26	0	0.851675	NaN	0	26	H9JPE1
46	59.57	0.28364	4714800	12	0	0.849704	NaN	0	12	H9JQL7
47	46.592	2.5788	23107000	41	0	0.847741	-2.62273	0	41	H9JR53
48	56.036	2.84	14844000	119	0	0.845788	NaN	0	119	H9JUJ9
49	56.036	2.84	14844000	122	0	0.843844	NaN	0	122	H9JUJ9
50	71.879	0.65455	12421000	101	0	0.841908	NaN	0	101	H9JYB7
51	71.879	0.65455	12421000	104	0	0.839982	NaN	0	104	H9JYB7
52	80.688	2.1009	352580	49	0	0.838091	NaN	0	49	E9JEH5
53	40.237	-1.5694	3348500	111	0	0.836182	0.0449429	0	111	H9IWR1
54	61.765	-3.0988	2552500	4	0	0.83239	NaN	0	4	H9IXH6
55	61.765	-3.0988	2552500	6	0	0.817604	NaN	0	6	H9IXH6
56	40.515	-0.79655	10135000	9	0	0.830552	-0.217958	0	9	H9IYB6
57	76.98	1.618	1051500000	197	0	0.828677	4.43861	0	197	H9J5R1
58	76.98	1.618	1051500000	199	0	0.826811	4.43861	0	199	H9J5R1
59	48.255	-3.1861	25694000	279	0	0.824953	2.34091	0	279	H9JDI2
60	84.743	-1.6119	2682700	249	0	0.823103	-0.389269	0	249	H9JE43
61	65.423	4.0284	327600000	627	0	0.821262	NaN	0	627	H9JGB3
62	55.815	-1.2928	8774800	14	0	0.819429	NaN	0	14	H9JJF1
63	90.149	0.036696	41651000	62	0	0.853656	NaN	0	62	H9JLH4
64	80.688	2.1009	352580	44	0	0.834282	NaN	0	44	E9JEH5
65	71.976	-2.8963	1320900	423	0	0.855646	0.426334	0	423	H9IVY6
66	84.743	-1.6119	2682700	242	0	0.882356	-0.389269	0	242	H9JE43
67	48.423	-0.82988	8774800	9	0	0.895229	NaN	0	9	H9JJF1
68	46.353	1.3074	53924000	268	0	0.893051	0.959637	0	268	H9JPN6
69	59.57	0.28364	4714800	6	0	0.890883	NaN	0	6	H9JQL7
70	46.592	2.5788	23107000	40	0	0.888755	-2.62273	0	40	H9JR53
71	49.34	0.13394	14596000	42	0	0.886609	0.24865	0	42	H9IVP6
72	49.34	0.13394	14596000	43	0	0.884472	0.24865	0	43	H9IVP6
73	49.34	0.13394	14596000	47	0	0.859653	0.24865	0	47	H9IVP6
74	49.34	0.13394	14596000	48	0	0.880249	0.24865	0	48	H9IVP6
75	41.366	3.3193	10010000	83	0	0.878144	NaN	0	83	H9IWZ2
76	55.841	0.7805	2428300	5	0	0.876048	-0.785416	0	5	H9IYA2
77	51.939	2.6753	3781100	313	0	0.873962	1.13476	0	313	H9IYQ9
78	51.939	2.6753	3781100	314	0	0.871886	1.13476	0	314	H9IYQ9
79	59.983	-2.4421	483060000	562	0	0.86982	0.414298	0	562	H9J0Z8
80	59.983	-2.4421	483060000	565	0	0.867764	0.414298	0	565	H9J0Z8
81	51.211	3.1765	2751900	60	0	0.865736	-0.522928	0	60	H9J5D1
82	48.354	2.806	52722000	2	0	0.863699	-0.988135	0	2	H9J6F7
83	45.873	-1.7093	4345000	561	0	0.861671	1.71627	0	561	H9JDR8
84	44.254	2.6	840830	140	0	1	NaN	0	140	H9JFF2
85	55.806	2.2283	1346100	7	0	0.857645	NaN	0	7	H9JLY6
86	77.357	1.7919	1098800000	225	0	1	-0.380488	0	225	H9JPC9
87	60.669	1.4705	71120000	228	0	1	NaN	0	228	H9JPC9
88	87.083	-1.7269	16190000	291	0	1	NaN	0	291	H9JT35
89	87.083	-1.7269	16190000	295	0	1	NaN	0	295	H9JT35
90	59.792	1.9915	11806000	179	0	1	NaN	0	179	H9JVF2
91	59.792	1.9915	11806000	190	0	1	NaN	0	190	H9JVF2
92	57.578	1.7956	24839000	445	0	1	NaN	0	445	H9JWY0
93	57.578	1.7956	24839000	448	0	1	NaN	0	448	H9JWY0
94	55.337	1.2767	8314800	5	0	1	0.421427	0	5	H9JXB0
95	55.337	1.2767	8314800	8	0	1	0.421427	0	8	H9JXB0
96	45.357	-1.652	47608000	232	0	1	NaN	0	232	H9ITI0
97	45.357	-1.652	47608000	242	0	1	NaN	0	242	H9ITI0
98	45.357	-1.652	47608000	245	0	1	NaN	0	245	H9ITI0
99	41.366	3.3193	10010000	86	0	1	NaN	0	86	H9IWZ2
100	52.853	1.5739	11177000	57	0	1	NaN	0	57	H9IXI7
101	55.841	0.7805	2428300	11	0	1	-0.785416	0	11	H9IYA2
102	42.708	-1.9821	2229100	395	0	1	0.145916	0	395	H9IZI4
103	42.708	-1.9821	2229100	408	0	1	0.145916	0	408	H9IZI4
104	51.211	3.1765	2751900	48	0	1	-0.522928	0	48	H9J5D1
105	51.211	3.1765	2751900	51	0	1	-0.522928	0	51	H9J5D1
106	48.354	2.806	52722000	3	0	1	-0.988135	0	3	H9J6F7
107	42.618	-3.5778	5549100	331	0	1	1.50955	0	331	H9J6Y7
108	42.618	-3.5778	5549100	332	0	1	1.50955	0	332	H9J6Y7
109	45.873	-1.7093	4345000	565	0	1	1.71627	0	565	H9JDR8
110	44.254	2.6	840830	149	0	1	NaN	0	149	H9JFF2
111	40.428	0.059703	58646000	129	0	1	1.53155	0	129	H9JFI5
112	53.403	-3.3714	1051700	9215	0	1	0.48673	0	9215	H9JLR9
113	53.403	-3.3714	1051700	9218	0	1	0.48673	0	9218	H9JLR9
114	55.806	2.2283	1346100	8	0	1	NaN	0	8	H9JLY6
115	50.505	-2.0694	1271200	1122	0	1	-1.17874	0	1122	H9JNI1
116	80.165	-3.9978	5324700	620	0	1	-1.51345	0	620	H9JSK5
117	80.165	-3.9978	5324700	621	0	1	-1.51345	0	621	H9JSK5
118	49.106	2.7913	46147000	60	0	1	1.89709	0	60	H9JSY6
119	49.106	2.7913	46147000	67	0	1	1.89709	0	67	H9JSY6
120	87.083	-1.7269	16190000	294	0	1	NaN	0	294	H9JT35
121	59.792	1.9915	11806000	184	0	1	NaN	0	184	H9JVF2
122	40.767	3.9447	827500	10	0	1	NaN	0	10	H9JVN0
123	40.767	3.9447	827500	12	0	1	NaN	0	12	H9JVN0
124	55.337	1.2767	8314800	9	0	1	0.421427	0	9	H9JXB0
125	41.366	3.3193	10010000	97	0	1	NaN	0	97	H9IWZ2
126	42.618	-3.5778	5549100	348	0	1	1.50955	0	348	H9J6Y7
127	42.618	-3.5778	5549100	350	0	1	1.50955	0	350	H9J6Y7

	Protein identifiers (continued)							
	Sequence window	Phospho (STY) Probabilities	id	Fasta headers	Unique identifier	Number of Phospho (STY)	Amino acid	Protein group IDs
1	TVQSSNTTDEIIRDASGAVIEEQITTK	DAS(1)GAVIEEQITTK	783	Fibroin heavy chain OS	UID784	1	S	1172
2	RGNDDLLKLNFVPTNSIELEMKKILI	LNFVPTNS(1)IELEMKK	110	Uncharacterized protein OS	UID111	1	S	219
3	SSYSSSSTVNGKTVSSGGVSELTND	TVS(0.003)S(0.997)GGVSELTNDGK	173	Uncharacterized protein OS	UID174	1	S	301
4	CTVLTIAHRLNTVIDSNKILVLDAGR	LNTVIDS(1)NKILVLDAGR	213	Uncharacterized protein OS	UID214	1	S	344
5	MAGLSRRRFATVVTA	MAGLS(1)RRR	239	Uncharacterized protein OS	UID240		S	390
6	PGDDFEDNDDDTSESYEDGSEEV	AS(0.001)PGDDFEDNDDDT(0.095)S	283	Uncharacterized protein OS	UID284	1	S	474
7	MNKIYSNIKGGAINRS	NKIY(0.126)S(0.874)NIK	402	PID domain-containing protein OS	UID403		S	661
8	MPIKALPSGRAMETPMI	MPIKALPS(1)GR	449	Uncharacterized protein OS;Uncharacterized prote	UID450		S	737
9	MALDNLSERDRAILRSI	MALDNLS(1)ERDR	751	TPR REGION domain-containing protein OS	UID752	1	S	1125
10	RYSPPCIPRSRTMDVSVGVPAHPAK	SRT(0.002)MDVS(0.998)VGVPAHPAK	765	Pribosyltran_N domain-containing protein OS	UID766	1	S	1153
11	CGLPRVGWQIDPFGHSRELASLLSA	VGWQIDPFGHS(1)R	776	Glyco_hydro_38N domain-containing protein OS	UID777		S	1162
12	GKNEKMIKTFVITTDSDGNESIVEEI	TFVITT(0.002)DS(0.976)DGNES(0.02	784	Fibroin heavy chain OS	UID785	1;2	S	1172
13	NRDTVEVKNRKYNAASSESSYLNKD	YNAAS(0.995)S(0.005)ESSYLNK	790	Sericin 1 OS	UID791	1	S	1174
14	QMEIAAIKRDRLDVMTKKQMNVF	DRLDVMT(1)KK	888	Uncharacterized protein OS	UID889	1	T	155
15	DVQKLQQQLQDIKEQTMCPICLDRI	EQT(1)MCPICLDR	982	Uncharacterized protein OS	UID983	1	T	328
16	MQTNLKTKEMFIVRALI	QTNLKT(1)K	1055	SEC7 domain-containing protein OS	UID1056		T	446
17	MHDATWAKSKHYVA	MHDAT(1)WAK	1106	Uncharacterized protein OS	UID1107	1	T	539
18	MCITLYANKETQGV	MCIT(0.888)LY(0.112)ANK	1325	Protein kinase domain-containing protein OS	UID1326	1	T	926
19	MEEALRTKDKELATLLEI	MEEALRT(1)KDK	1417	Uncharacterized protein OS	UID1418		T	1103
20	MKAAPFLAMMTLKQLAADE	KAAPFLAMMT(1)LK	1436	Uncharacterized protein OS	UID1437	1	T	1150
21	MSEGTATIRTRKFMT	MS(0.004)EGT(0.988)AT(0.007)IR	1459	40S ribosomal protein S24 OS	UID1460		T	1232
22	EGKMTRFDCRVTGRPYPEVTWFIN	VT(0.225)GRPY(0.775)PEVTWFINDR	1502	Uncharacterized protein OS	UID1503		Y	152
23	GKKIYFKIRHRLENRYMKLGNDADR	HRLENRY(1)MK	1589	Uncharacterized protein OS	UID1590	1	Y	428
24	SLKKLETNHMKHIKQYGNDEATNR	HIKQY(1)GNDEATNRQR	1626	Gln-synt_C domain-containing protein OS	UID1627		Y	606
25	SNQKYRDRVKELSMIYHDRPVSPGA	ELS(0.167)MIY(0.833)HDR	1688	UDP-glucuronosyltransferase OS	UID1689		Y	835
26	AAYAQSKLAQLMMARYINKIMEEN	LAQLMMARY(1)INK	1733	Uncharacterized protein OS	UID1734		Y	977
27	LVLLKTHLLKKSLSHYPNIKYTVESLN	KSLS(0.007)HY(0.993)PNIK	1735	Uncharacterized protein OS	UID1736		Y	980
28	MYNLETYLCTKIN	Y(0.793)NLET(0.179)Y(0.028)LCTKIM	1736	Uncharacterized protein OS	UID1737	1	Y	986
29	DISELQILKIEDIMDYANKENYGSQN	IEDIMDY(1)ANK	1737	Uncharacterized protein OS	UID1738	1	Y	990
30	AVFKVGYYKMHPEIPSELSLRAKNF	MHPEIPS(1)ELS(1)LRAKNFILR	37	ASK1 OS	UID1828	2	S	99
31	KVGYYKMHPEIPSELSLRAKNFILRC	MHPEIPS(1)ELS(1)LRAKNFILR	38	ASK1 OS	UID1829	2	S	99
32	MVARRNLHVGVVHSASKPNIEKI	RNLHVGVVHS(1)AS(1)K	104	Ribosomal_L2_C domain-containing protein OS	UID1895	2	S	207
33	MVARRNLHVGVVHSASKPNIEKPK	RNLHVGVVHS(1)AS(1)K	105	Ribosomal_L2_C domain-containing protein OS	UID1896	2	S	207
34	QCISAYVPREQRAHPSPVRTPSSPAI	EQRAHPS(0.995)PVRT(0.947)PS(0.04	112	F-box domain-containing protein OS	UID1903	2	S	222
35	VDRKSWKNLAYLRNRSFVSPFDYAF	NRS(1)FVS(0.576)PFDY(0.424)AR	117	ANK_REP_REGION domain-containing protein OS	UID1908		S	231
36	KSWKNLAYLRNRSFVSPFDYARSAA	NRS(1)FVS(0.896)PFDY(0.104)AR	118	ANK_REP_REGION domain-containing protein OS	UID1909		S	231
37	MGEVMRERTSIQMQPMA	MGEVMRERT(0.978)S(0.979)IQMQP	141	G_PROTEIN_RECEP_F1_2 domain-containing prote	UID1932	2	S	265
38	ILERTQRKVDKIIENSNMFINPIMTF	IIENS(1)NMFINPIMT(0.543)FY(0.457	252	Uncharacterized protein OS	UID2043	2	S	406
39	MSSNIMLPESPDF	S(0.227)S(0.773)NIMLPES(0.266)PD	362	WD_REPEATS_REGION domain-containing protein	UID2153		S	597
40	DTTEHLKSLTDMETDSDIISKDDTQI	S(0.517)LT(0.397)DMET(0.105)DS(0.	372	Uncharacterized protein OS	UID2163		S	608
41	WDLAKFNKVSTKIGSSMKSVGEVM	IGS(0.936)S(0.936)MKS(0.127)VGEV	376	Uncharacterized protein OS	UID2167	2	S	615
42	AQPTDAERGEATQATSASTTTTTTA	GEAT(0.171)QAT(0.801)S(0.757)AS(	445	Cyclic nucleotide-binding domain-containing protei	UID2236	2	S	734
43	TVTAKKVDVSLQTNVSRRIGLTLSDI	KVDVS(0.501)LQT(0.501)NVS(0.998)	470	Uncharacterized protein OS	UID2261		S	779
44	RKPGRSAKVVLSKTASATMLPRDIV	VVLS(0.016)KT(0.034)AS(0.975)AT(0	534	Uncharacterized protein OS	UID2325	2	S	858
45	SGSDYTPVAEFYAEKSVFVTGGTGF	AS(0.428)CVGANY(0.409)S(0.113)S(	595	Fatty acyl-CoA reductase OS	UID2386		S	930
46	MARALYDNIAESPDELAFRRG	ARALY(1)DNIAES(1)PDELAFR	620	SH3 domain-containing protein OS	UID2411	2	S	958
47	ETNVFHTHIIPKVQYSILHNQILKGTI	VQY(1)S(1)ILHNQILK	628	Uncharacterized protein OS	UID2419	2	S	974
48	GNLYAASAEPTYVKDSHVSNVNIEN	DS(1)HVS(1)NVNIEMPNRMSGTNFR	709	Uncharacterized protein OS	UID2500		S	1075
49	YAASAEPTYVKDSHVSNVNIEMPNI	DS(1)HVS(1)NVNIEMPNRMSGTNFR	710	Uncharacterized protein OS	UID2501		S	1075
50	GWWTKESAFMSLIAISLISRSMCDL	ESAFMSLIAIS(1)LIS(1)R	774	ABC transmembrane type-1 domain-containing pro	UID2565	2	S	1161
51	TKESAFMSLIAISLISRSMCDLWLIQ	ESAFMSLIAIS(1)LIS(1)R	775	ABC transmembrane type-1 domain-containing pro	UID2566	2	S	1161
52	YKQSINSKRRYEQIETIAQLLKVLEIRI	Y(1)EQIET(1)IAQLLK	858	Fadd OS	UID2649		T	100
53	AYVPREQRAHPSPVRTPSSPARRPA	EQRAHPS(0.995)PVRT(0.947)PS(0.04	916	F-box domain-containing protein OS	UID2707	2	T	222
54	MKITVTRMDTAMK	MKIT(0.81)VT(0.81)RMDT(0.381)AM	927	Uncharacterized protein OS	UID2718		T	244
55	MKITVTRMDTAMKAI	MKIT(0.81)VT(0.81)RMDT(0.381)AM	928	Uncharacterized protein OS	UID2719		T	244
56	MGEVMRERTSIQMQPMA	MGEVMRERT(0.978)S(0.979)IQMQP	939	G_PROTEIN_RECEP_F1_2 domain-containing prote	UID2730	2	T	265
57	VASPEPRGEDAIDGVTKTKSSSDDD	NGDVASPEPRGEDAIDGVT(1)KT(1)K	1062	Uncharacterized protein OS	UID2853	2	T	464
58	SPEPRGEDAIDGVTKTKSSSDDDRW	NGDVASPEPRGEDAIDGVT(1)KT(1)K	1063	Uncharacterized protein OS	UID2854	2	T	464
59	AARLMAQICKTFNTSTNNLQTRVTF	LMAQICKTFNT(0.01)S(0.487)T(0.947	1179	TAF6_C domain-containing protein OS	UID2970		T	669
60	NRVERVKRYLMRCGLTKDSLKNIPR	Y(1)LMRCGLT(1)K	1197	Uncharacterized protein OS	UID2988	2	T	688
61	AAQPTDAERGEATQATSASTTTTTT	GEAT(0.171)QAT(0.801)S(0.757)AS(	1225	Cyclic nucleotide-binding domain-containing protei	UID3016	2	T	734
62	MHDVRKEEYGWESTVRLMVGR	MHDVRKEEY(0.631)GWES(0.617)T(0	1254	Uncharacterized protein OS	UID3045	2	T	806
63	PGRSAKVVLSKTASATMLPRDIVAE	VVLS(0.016)KT(0.034)AS(0.975)AT(0	1289	Uncharacterized protein OS	UID3080	2	T	858
64	SVKEIYKQSINSKRRYEQIETIAQLLK	Y(1)EQIET(1)IAQLLK	1484	Fadd OS	UID3275		Y	100
65	AVDILCQCRQTLMYTYVFAYYLRKN	QT(0.091)LMY(0.629)T(0.28)Y(0.999	1521	RBR-type E3 ubiquitin transferase OS	UID3312		Y	200
66	GPAMRRPNRVERVKRYLMRCGLTK	Y(1)LMRCGLT(1)K	1651	Uncharacterized protein OS	UID3442	2	Y	688
67	MHDVRKEEYGWESTVRLI	MHDVRKEEY(0.867)GWES(0.567)T(0	1678	Uncharacterized protein OS	UID3469	2	Y	806
68	VVGKQMIDDALNGKMYSVYTTSG	MY(0.944)S(0.139)VY(0.388)T(0.479	1717	Uncharacterized protein OS	UID3508		Y	937
69	MARALYDNIAESPDEL	ARALY(1)DNIAES(1)PDELAFR	1724	SH3 domain-containing protein OS	UID3515	2	Y	958
70	VETNVFHTHIIPKVQYSILHNQILKG	VQY(1)S(1)ILHNQILK	1731	Uncharacterized protein OS	UID3522	2	Y	974
71	GDDDLSSVFSRSTRASSVACSSVVS	S(0.249)T(0.249)RAS(0.793)S(0.793)	91	Uncharacterized protein OS	UID3672		S	192
72	DDDLSSVFSRSTRASSVACSSVVSN	S(0.249)T(0.249)RAS(0.793)S(0.793)	92	Uncharacterized protein OS	UID3673		S	192
73	SSVFSRSTRASSVACSSVVSNIAWG	S(0.249)T(0.249)RAS(0.793)S(0.793)	93	Uncharacterized protein OS	UID3674		S	192
74	SVFSRSTRASSVACSSVVSNIAWGE	S(0.249)T(0.249)RAS(0.793)S(0.793)	94	Uncharacterized protein OS	UID3675		S	192
75	KKSHVKTESVYEITDSIETPYNVANT	KS(0.268)HVKT(0.664)ES(0.137)VY(0	116	C2H2-type domain-containing protein OS	UID3697		S	229
76	MTESSPDEPSTKRKD	MT(0.432)ES(0.572)S(0.993)PDEPS(	140	Uncharacterized protein OS	UID3721	3	S	264
77	YFKQVTRRVAEIKAVSSPEERGVLIQ	AVS(1)S(1)PEERGVLIQILES(0.006)T(0	142	Uncharacterized protein OS	UID3723	3	S	269
78	FKQVTRRVAEIKAVSSPEERGVLIQI	AVS(1)S(1)PEERGVLIQILES(0.006)T(0	143	Uncharacterized protein OS	UID3724	3	S	269
79	SDLSLPQSRYSVDLNSRLSRELNTPN	PSS(0.001)DLS(0.049)LPQS(0.43)RY(	201	Uncharacterized protein OS	UID3782		S	327
80	SLPQSRYSVDLNSRLSRELNTPNSRY	PSS(0.001)DLS(0.049)LPQS(0.43)RY(	202	Uncharacterized protein OS	UID3783		S	327
81	HKFTGNTEVLSNPVDSEVVLKTCLE	FT(1)GNT(0.981)EVLS(0.142)NPVDS(	275	Uncharacterized protein OS	UID3856	3	S	452
82	MSTAKQVNGNT	S(0.864)T(0.883)AKQVNGNT(0.74)T(	285	Uncharacterized protein OS	UID3866	3	S	477
83	EGPYMVKMLAPDPPLSINPTEIYHK	MLAPDPPLS(1)INPT(1)EIY(1)HK	421	Elongator complex protein 3 OS	UID4002		S	678
84	PRRTSLAPELCRQLLSYMLVHPALTH	QLLS(1)Y(1)MLVHPALT(1)HHDQR	439	SAM domain-containing protein OS	UID4020		S	716
85	MSSLLRSTKFVRYFTGA	S(0.425)S(0.575)LLRS(1)T(1)K	540	Uncharacterized protein OS	UID4121	3	S	866
86	RVCARRQILIDLLSESTRSYGLAAEM	QILIDLLS(0.999)ES(0.812)T(0.998)RS	592	Casc1_N domain-containing protein OS	UID4173	3	S	927
87	ARRQILIDLLSESTRSYGLAAEMPNR	QILIDLLS(0.996)ES(0.028)T(0.964)RS	593	Casc1_N domain-containing protein OS	UID4174	3	S	927
88	PLTGTEALRIALGLASAETSAERPRR	IALGLAS(1)AET(1)S(1)AERPR	664	Spaetzle domain-containing protein OS	UID4245	3	S	1023
89	TEALRIALGLASAETSAERPRRQAVN	IALGLAS(1)AET(1)S(1)AERPR	665	Spaetzle domain-containing protein OS	UID4246	3	S	1023
90	SEAPSNKQTVFSRLGSNESVTNATK	LGS(0.829)NES(0.243)VT(0.928)NAT	731	Uncharacterized protein OS	UID4312		S	1093
91	SRLGSNESVTNATKPSAKPIYARLGA	LGS(0.829)NES(0.243)VT(0.928)NAT	732	Uncharacterized protein OS	UID4313		S	1093
92	FKMERHGVTQVLSRLSYISALGMM	LS(0.997)Y(0.997)IS(0.998)ALGMMT	747	DNA-directed RNA polymerase subunit beta OS	UID4328	3	S	1122
93	ERHGVTQVLSRLSYISALGMMTRVI	LS(0.997)Y(0.997)IS(0.998)ALGMMT	748	DNA-directed RNA polymerase subunit beta OS	UID4329	3	S	1122
94	MAGESAVSTASKPQ	MAGES(0.999)AVS(0.998)T(0.997)AS	758	COX6C domain-containing protein OS	UID4339	3	S	1136
95	MAGESAVSTASKPQMR	MAGES(0.999)AVS(0.998)T(0.997)AS	759	COX6C domain-containing protein OS	UID4340	3	S	1136
96	HPFFKKAKDRKYLTQTLVAIGPSME	KY(0.022)LT(0.013)QT(0.967)LVAIGP	879	Protein kinase domain-containing protein OS	UID4460	3	T	147
97	KYLTQTLVAIGPSMETRVTKASKKH	KY(0.022)LT(0.013)QT(0.967)LVAIGP	880	Protein kinase domain-containing protein OS	UID4461	3	T	147
98	TQTLVAIGPSMETRVTKASKKHSAT	KY(0.022)LT(0.013)QT(0.967)LVAIGP	881	Protein kinase domain-containing protein OS	UID4462	3	T	147
99	HVKTESVYEITDSIETPYNVANTVNA	KS(0.268)HVKT(0.664)ES(0.137)VY(0	922	C2H2-type domain-containing protein OS	UID4503		T	229
100	KNLASHVKVSLGSIGTPLPITSERFIG	NLAS(0.002)HVKVS(0.542)LGS(0.465	929	Uncharacterized protein OS	UID4510	3	T	245
101	MTESSPDEPSTKRKDFICGV	MT(0.432)ES(0.572)S(0.993)PDEPS(	938	Uncharacterized protein OS	UID4519	3	T	264
102	NINSVRNSHYRANRGTVVIVHGWN	NS(0.523)HY(0.479)RANRGT(0.998)V	961	Uncharacterized protein OS	UID4542	3	T	298
103	RGTVVIVHGWNNNGNTQMNPLIF	NS(0.523)HY(0.479)RANRGT(0.998)V	962	Uncharacterized protein OS	UID4543	3	T	298
104	DVDKHIPLLGIAHKFTGNTEVLSNP	FT(1)GNT(0.981)EVLS(0.142)NPVDS(	1058	Uncharacterized protein OS	UID4639	3	T	452
105	KHIPLLGIAHKFTGNTEVLSNPVDSE	FT(1)GNT(0.981)EVLS(0.142)NPVDS(	1059	Uncharacterized protein OS	UID4640	3	T	452
106	MSTAKQVNGNTT	S(0.864)T(0.883)AKQVNGNT(0.74)T(	1069	Uncharacterized protein OS	UID4650	3	T	477
107	EGWLEPLLDRIARNKTTVVCPVIDVI	NKT(1)T(1)VVCPVIDVIDDNT(0.399)LI	1083	Polypeptide N-acetylgalactosaminyltransferase OS	UID4664	4	T	488
108	GWLEPLLDRIARNKTTVVCPVIDVID	NKT(1)T(1)VVCPVIDVIDDNT(0.399)LI	1084	Polypeptide N-acetylgalactosaminyltransferase OS	UID4665	4	T	488
109	MVKMLAPDPPLSINPTEIYHKHVGI	MLAPDPPLS(1)INPT(1)EIY(1)HK	1190	Elongator complex protein 3 OS	UID4771		T	678
110	LCRQLLSYMLVHPALTHHDQRTLTC	QLLS(1)Y(1)MLVHPALT(1)HHDQR	1212	SAM domain-containing protein OS	UID4793		T	716
111	TQERYSICQENHGRITVEQHNTYITC	IT(0.969)VEQHNT(0.292)Y(0.272)IT(	1213	Uncharacterized protein OS	UID4794		T	719
112	SPEKTTVKEIVTQTDTDVTKIAENLN	TTVKEIVT(0.596)QT(0.69)DT(0.95)D	1291	Uncharacterized protein OS	UID4872		T	861
113	KTTVKEIVTQTDTDVTKIAENLNEEE	TTVKEIVT(0.596)QT(0.69)DT(0.95)D	1292	Uncharacterized protein OS	UID4873		T	861
114	MSSLLRSTKFVRYFTGAA	S(0.425)S(0.575)LLRS(1)T(1)K	1295	Uncharacterized protein OS	UID4876	3	T	866
115	CLHYRDFNASAYVADTIIEAVESSKR	DFNAS(0.668)AY(0.505)VADT(0.827	1314	TIR domain-containing protein OS	UID4895	3	T	904
116	TNEIEDLSKIQEYLETTNKQYEVQVG	IQEY(1)LET(1)T(1)NK	1369	Uncharacterized protein OS	UID4950	3	T	1010
117	NEIEDLSKIQEYLETTNKQYEVQVGT	IQEY(1)LET(1)T(1)NK	1370	Uncharacterized protein OS	UID4951	3	T	1010
118	LVIKYGKECEEYQVVTEDGYILTLFRI	ECEEY(0.002)QVVT(0.998)EDGY(1)IL	1374	Lipase OS	UID4955	3	T	1019
119	ECEEYQVVTEDGYILTLFRIPGDVNR	ECEEY(0.002)QVVT(0.998)EDGY(1)IL	1375	Lipase OS	UID4956	3	T	1019
120	GTEALRIALGLASAETSAERPRRQAV	IALGLAS(1)AET(1)S(1)AERPR	1377	Spaetzle domain-containing protein OS	UID4958	3	T	1023
121	NKQTVFSRLGSNESVTNATKPSAKF	LGS(0.829)NES(0.243)VT(0.928)NAT	1409	Uncharacterized protein OS	UID4990		T	1093
122	MSFEYTTKITVTRMNGNGD	MS(0.699)FEY(0.15)T(0.172)T(0.056	1413	Uncharacterized protein OS	UID4994		T	1097
123	MSFEYTTKITVTRMNGNGDV	MS(0.699)FEY(0.15)T(0.172)T(0.056	1414	Uncharacterized protein OS	UID4995		T	1097
124	MAGESAVSTASKPQMRG	MAGES(0.999)AVS(0.998)T(0.997)AS	1432	COX6C domain-containing protein OS	UID5013	3	T	1136
125	DSIETPYNVANTVNAYAEKVECTAK	KS(0.268)HVKT(0.664)ES(0.137)VY(0	1529	C2H2-type domain-containing protein OS	UID5110		Y	229
126	VVCPVIDVIDDNTLEYHYRDSSSVN	NKT(1)T(1)VVCPVIDVIDDNT(0.399)LI	1602	Polypeptide N-acetylgalactosaminyltransferase OS	UID5183	4	Y	488
127	CPVIDVIDDNTLEYHYRDSSSVNVG	NKT(1)T(1)VVCPVIDVIDDNT(0.399)LI	1603	Polypeptide N-acetylgalactosaminyltransferase OS	UID5184	4	Y	488

128	19.6328	NaN	NaN	NaN	18.8374	NaN	18.9934	15.9189	1	0.0371185	45.873	23.751	45.873	-1.7093	4345000	568	0	1	1.71627	0	568	H9JDR8	MLAPDPPLSINPTEIYHKHVGMLPN	MLAPDPPLS(1)INPT(1)EIY(1)HK	1647	Elongator complex protein 3 OS	UID5228		Y	678
129	NaN	18.2952	17.1833	16.4748	NaN	NaN	NaN	NaN	1	0.0380963	44.254	13.218	44.254	2.6	840830	141	0	1	NaN	0	141	H9JFF2	RRTSLAPELCRQLLSYMLVHPALTHI	QLLS(1)Y(1)MLVHPALT(1)HHDQR	1656	SAM domain-containing protein OS	UID5237		Y	716
130	NaN	NaN	18.1186	NaN	21.0697	18.4453	NaN	19.3811	1	0.00816905	80.165	26.16	80.165	-3.9978	5324700	617	0	1	-1.51345	0	617	H9JSK5	LSTTNEIEDLSKIQEYLETTNKQYEVC	IQEY(1)LET(1)T(1)NK	1744	Uncharacterized protein OS	UID5325	3	Y	1010
131	23.8724	20.4661	22.8989	20.5989	NaN	20.062	NaN	NaN	0.999808	0.0373914	49.106	14.831	49.106	2.7913	46147000	64	0	1	1.89709	0	64	H9JSY6	YGKECEEYQVVTEDGYILTLFRIPGD	ECEEY(0.002)QVVT(0.998)EDGY(1)IL	1748	Lipase OS	UID5329	3	Y	1019
132	NaN	NaN	NaN	NaN	NaN	NaN	NaN	22.4819	0.997454	0.0225831	57.578	25.028	57.578	1.7956	24839000	446	0	1	NaN	0	446	H9JWY0	KMERHGVTQVLSRLSYISALGMMT	LS(0.997)Y(0.997)IS(0.998)ALGMMT	1772	DNA-directed RNA polymerase subunit beta OS	UID5353	3	Y	1122
133	24.5224	21.5541	21.6549	NaN	23.4884	NaN	21.8151	22.481	1	0.0228279	77.644	39.445	75.102	-3.506	501150000	96	0.0053377	1	-0.0177186	-0.0149167	96	H9JIJ0	LKSILTICLKRFEERTIVIKEYHTVQAN	T(1)IVIKEY(1)HT(1)VQANK	1246	BTB domain-containing protein OS	UID4827	3	T	789
134	24.5224	21.5541	21.6549	NaN	23.4884	NaN	21.8151	22.481	1	0.0228279	77.644	39.445	75.102	-3.506	501150000	104	0.0053377	1	-0.0177186	-0.0149167	104	H9JIJ0	LKRFEERTIVIKEYHTVQANKWQYL	T(1)IVIKEY(1)HT(1)VQANK	1247	BTB domain-containing protein OS	UID4828	3	T	789
135	24.5224	21.5541	21.6549	NaN	23.4884	NaN	21.8151	22.481	1	0.0228279	77.644	39.445	75.102	-3.506	501150000	102	0.0053377	1	-0.0177186	-0.0149167	102	H9JIJ0	ICLKRFEERTIVIKEYHTVQANKWQY	T(1)IVIKEY(1)HT(1)VQANK	1671	BTB domain-containing protein OS	UID5252	3	Y	789
136	19.2597	NaN	19.1835	18.5117	20.8101	18.7121	17.3367	NaN	1	0.0245832	50.149	14.438	50.149	2.9823	6002700	251	0.0101568	1	0.0319818	0.0281167	251	H9JRZ6	SSKIDTLVNKIIPMMSVSEKMDEVV	IDT(1)LVNKIIPMMS(1)VS(1)EK	641	Fibrinogen C-terminal domain-containing protein C	UID4222	3	S	992
137	19.2597	NaN	19.1835	18.5117	20.8101	18.7121	17.3367	NaN	1	0.0245832	50.149	14.438	50.149	2.9823	6002700	253	0.0101568	1	0.0319818	0.0281167	253	H9JRZ6	KIDTLVNKIIPMMSVSEKMDEVWD	IDT(1)LVNKIIPMMS(1)VS(1)EK	642	Fibrinogen C-terminal domain-containing protein C	UID4223	3	S	992
138	19.2597	NaN	19.1835	18.5117	20.8101	18.7121	17.3367	NaN	1	0.0245832	50.149	14.438	50.149	2.9823	6002700	241	0.0101568	1	0.0319818	0.0281167	241	H9JRZ6	ASSTPSPVTKSSKIDTLVNKIIPMMS	IDT(1)LVNKIIPMMS(1)VS(1)EK	1357	Fibrinogen C-terminal domain-containing protein C	UID4938	3	T	992
139	27.5035	24.865	18.2941	26.1422	24.6009	23.9526	23.184	24.8139	0.999994	0.0357842	52.432	8.403	52.432	-1.5119	1127500000	201	0.01027	1	0.0633354	0.0291316	201	D3VW60	SFVQHLLNHFDIEDETTELIEKPELFA	SFVQHLLNHFDIEDET(1)T(1)ELIEK	853	Origin recognition complex subunit 5 OS	UID2644	2	T	92
140	27.5035	24.865	18.2941	26.1422	24.6009	23.9526	23.184	24.8139	0.999994	0.0357842	52.432	8.403	52.432	-1.5119	1127500000	202	0.01027	1	0.0633354	0.0291316	202	D3VW60	FVQHLLNHFDIEDETTELIEKPELFAI	SFVQHLLNHFDIEDET(1)T(1)ELIEK	854	Origin recognition complex subunit 5 OS	UID2645	2	T	92
141	NaN	19.8393	NaN	19.5385	22.0301	NaN	17.1785	NaN	0.98787	0.0292224	41.86	18.806	41.86	-2.9228	14632000	136	0.0108147	1	0.0845594	0.033417	136	H9J4D1	SILVKRKFINFAKVQYTMMYRNIAC	FINFAKVQY(0.988)T(0.064)MMY(0.9	1585	Uncharacterized protein OS	UID3376	2	Y	411
142	NaN	19.8393	NaN	19.5385	22.0301	NaN	17.1785	NaN	0.948383	0.0292224	41.86	18.806	41.86	-2.9228	14632000	140	0.0108147	1	0.0845594	0.033417	140	H9J4D1	KRKFINFAKVQYTMMYRNIACARQ	FINFAKVQY(0.988)T(0.064)MMY(0.9	1586	Uncharacterized protein OS	UID3377	2	Y	411
143	19.1745	NaN	NaN	20.1372	21.1027	17.7382	19.8678	NaN	0.955499	0.000371388	111.09	65.39	111.09	1.2033	105960000	187	0.0213953	1	0.0862916	0.0608403	187	H9JQU0	DDDFSDLDEEIIRLFSSDEEIDDDKS\	LFS(0.955)S(0.045)DEEIDDDKSVIPLD	623	Uncharacterized protein OS	UID624	1	S	964
144	19.9806	18.3348	NaN	18.209	20.444	17.425	NaN	NaN	0.968734	0.0268554	64.454	33.343	64.454	1.8864	25304000	48	0.022641	1	-0.0930367	-0.064388	48	H9JBL8	WNDDGKVFRCEAGVITISRPECYAP	CEAGVIT(0.969)IS(0.031)RPECYAPCD	1153	Uncharacterized protein OS	UID1154		T	623
145	20.7292	21.0321	21.7458	19.6326	19.9286	NaN	21.612	20.9587	0.964894	0.0309976	66.682	21.929	66.682	2.8192	30023000	9	0.024664	1	-0.0481861	-0.0632412	9	H9ITG2	MSWLFGYSSKPPQPPPSD	MSWLFGYS(0.035)S(0.965)K	63	AAA domain-containing protein OS	UID64	1	S	144
146	16.478	17.1965	16.2808	NaN	18.7876	15.6168	NaN	15.7875	0.879925	0.0328632	43.405	9.6439	43.405	-1.6755	1108200	109	0.0247493	1	-0.0788682	-0.0676045	109	H9JEH6	DNDEFVHCKECGTKYTEKHVEAFIKI	Y(0.12)T(0.88)EKHVEAFIK	1199	Uncharacterized protein OS	UID1200		T	694
147	28.3737	NaN	17.9578	25.8594	24.9295	24.4441	22.7952	24.9264	0.998711	0.00226256	77.357	33.546	77.357	1.7919	1170000000	223	0.0264999	1	-0.210207	-0.0752673	223	H9JPC9	VVRVCARRQILIDLLSESTRSYGLAAI	QILIDLLS(0.999)ES(0.812)T(0.998)RS	591	Casc1 N domain-containing protein OS	UID4172	3	S	927
148	28.3737	NaN	17.9578	25.8594	24.9295	24.4441	22.7952	24.9264	0.997672	0.00226256	77.357	33.546	77.357	1.7919	1170000000	226	0.0264999	1	-0.210207	-0.0752673	226	H9JPC9	VCARRQILIDLLSESTRSYGLAAEMP	QILIDLLS(0.999)ES(0.812)T(0.998)RS	1326	Casc1 N domain-containing protein OS	UID4907	3	T	927
149	NaN	17.2731	NaN	16.6455	NaN	14.962	18.0261	18.2592	0.999961	0.0284978	83.869	23.35	83.869	-2.1665	2292200	314	0.0291595	1	-0.123116	-0.0825579	314	H9J2J2	VNKSETDAEFDKQPKTETIKLETQNI	T(1)ET(1)IKLETQNK	1008	DUF2040 domain-containing protein OS	UID2799	2	T	370
150	NaN	17.2731	NaN	16.6455	NaN	14.962	18.0261	18.2592	0.999923	0.0284978	83.869	23.35	83.869	-2.1665	2292200	316	0.0291595	1	-0.123116	-0.0825579	316	H9J2J2	KSETDAEFDKQPKTETIKLETQNKNI	T(1)ET(1)IKLETQNK	1009	DUF2040 domain-containing protein OS	UID2800	2	T	370
151	16.4763	18.026	17.2976	17.0916	NaN	16.9716	17.5677	NaN	0.846605	0.0246967	48.568	23.514	48.568	-0.048663	1496500	4	0.030732	1	-0.0467973	-0.0763589	4	H9J1Q4	MASTFIGNTTAIQTI	MAS(0.141)T(0.847)FIGNT(0.987)T(	991	Uncharacterized protein OS	UID4572	3	T	348
152	16.4763	18.026	17.2976	17.0916	NaN	16.9716	17.5677	NaN	0.986983	0.0246967	48.568	23.514	48.568	-0.048663	1496500	9	0.030732	1	-0.0467973	-0.0763589	9	H9J1Q4	MASTFIGNTTAIQTIFKRV:	MAS(0.141)T(0.847)FIGNT(0.987)T(	992	Uncharacterized protein OS	UID4573	3	T	348
153	16.4763	18.026	17.2976	17.0916	NaN	16.9716	17.5677	NaN	0.969869	0.0246967	48.568	23.514	48.568	-0.048663	1496500	10	0.030732	1	-0.0467973	-0.0763589	10	H9J1Q4	MASTFIGNTTAIQTIFKRVSI	MAS(0.141)T(0.847)FIGNT(0.987)T(	993	Uncharacterized protein OS	UID4574	3	T	348
154	26.4818	NaN	23.85	23.0156	NaN	22.8509	25.166	24.887	0.898029	0.0287247	42.647	20.252	42.647	1.9849	1367900000	2	0.0394276	1	0.147844	0.107595	2	H9JUP8	MSTVARSFEASQ	S(0.898)T(0.091)VARS(0.013)FEAS(0	711	Uncharacterized protein OS	UID2502	2	S	1076
155	26.4818	NaN	23.85	23.0156	NaN	22.8509	25.166	24.887	0.997191	0.0287247	42.647	20.252	42.647	1.9849	1367900000	11	0.0394276	1	0.147844	0.107595	11	H9JUP8	MSTVARSFEASQVVPDVIPK.	S(0.898)T(0.091)VARS(0.013)FEAS(0	712	Uncharacterized protein OS	UID2503	2	S	1076
156	18.6082	17.3651	NaN	21.6029	22.9891	16.9555	NaN	18.5686	0.997675	0.00824614	60.814	26.56	60.814	2.7795	12938000	4	0.0487114	1	-0.312354	-0.135882	4	H9J5W2	MQQSPFENIFNILG	QQS(0.998)PFENIFNILGGIT(0.002)E(	278	Uncharacterized protein OS	UID279	1	S	467
157	21.8304	21.1033	20.5694	20.707	21.5185	20.213	21.4632	21.2608	1	0.0380559	52.498	10.883	52.498	-1.4551	110300000	1348	0.0517443	1	-0.0613403	-0.118813	1348	H9IWD5	EEMEHQSQLEADRHDTLPEEPEFED	HDT(1)LPEEPEFEDIMEEPPEVLK	912	Uncharacterized protein OS	UID913	1	T	211
158	NaN	20.3353	NaN	19.5924	NaN	20.8045	19.0511	19.6604	0.99539	0.0187612	66.76	41.287	66.76	4.2106	7889900	490	0.0590501	1	0.125172	0.152843	490	E9JTY1	GYIDSDRDSDYGVTKSARKGTKGRI	LPDT(0.997)GY(0.999)IDS(0.004)DRI	39	KDM4 OS	UID3620		S	102
159	NaN	20.3353	NaN	19.5924	NaN	20.8045	19.0511	19.6604	0.996715	0.0187612	66.76	41.287	66.76	4.2106	7889900	474	0.0590501	1	0.125172	0.152843	474	E9JTY1	LKGDEMDPSEARLPDTGYIDSDRD:	LPDT(0.997)GY(0.999)IDS(0.004)DRI	861	KDM4 OS	UID4442		T	102
160	NaN	20.3353	NaN	19.5924	NaN	20.8045	19.0511	19.6604	0.996726	0.0187612	66.76	41.287	66.76	4.2106	7889900	488	0.0590501	1	0.125172	0.152843	488	E9JTY1	DTGYIDSDRDSDYGVTKSARKGTKG	LPDT(0.997)GY(0.999)IDS(0.004)DRI	862	KDM4 OS	UID4443		T	102
161	NaN	20.3353	NaN	19.5924	NaN	20.8045	19.0511	19.6604	0.998958	0.0187612	66.76	41.287	66.76	4.2106	7889900	476	0.0590501	1	0.125172	0.152843	476	E9JTY1	GDEMDPSEARLPDTGYIDSDRDSD'	LPDT(0.997)GY(0.999)IDS(0.004)DRI	1485	KDM4 OS	UID5066		Y	102
162	NaN	20.3353	NaN	19.5924	NaN	20.8045	19.0511	19.6604	0.996239	0.0187612	66.76	41.287	66.76	4.2106	7889900	485	0.0590501	1	0.125172	0.152843	485	E9JTY1	RLPDTGYIDSDRDSDYGVTKSARKG	LPDT(0.997)GY(0.999)IDS(0.004)DRI	1486	KDM4 OS	UID5067		Y	102
163	18.8863	NaN	19.8981	19.1638	NaN	18.9971	NaN	19.4839	0.903571	0.0317656	41.871	18.071	41.871	2.6828	3905100	66	0.0596457	1	0.0755482	0.14259	66	H9JNM0	SRLRDMLERRLATDLSDYTFWLQD/	RLAT(0.895)DLS(0.904)DY(0.895)T(0	574	Uncharacterized protein OS	UID4155	3	S	905
164	18.8863	NaN	19.8981	19.1638	NaN	18.9971	NaN	19.4839	0.894526	0.0317656	41.871	18.071	41.871	2.6828	3905100	63	0.0596457	1	0.0755482	0.14259	63	H9JNM0	TRLSRLRDMLERRLATDLSDYTFWL	RLAT(0.895)DLS(0.904)DY(0.895)T(0	1315	Uncharacterized protein OS	UID4896	3	T	905
165	18.8863	NaN	19.8981	19.1638	NaN	18.9971	NaN	19.4839	0.894526	0.0317656	41.871	18.071	41.871	2.6828	3905100	68	0.0596457	1	0.0755482	0.14259	68	H9JNM0	LRDMLERRLATDLSDYTFWLQDAKI	RLAT(0.895)DLS(0.904)DY(0.895)T(0	1709	Uncharacterized protein OS	UID5290	3	Y	905
166	17.8959	19.2348	NaN	17.3	18.7587	NaN	NaN	17.1892	0.936063	0.0321573	53.924	15.311	53.924	3.8585	2288600	8	0.0611874	1	0.169631	0.162776	8	H9JUJ5	MSAAEFDSIEQSNDILTR	S(0.064)AAEFDS(0.936)IEQSNDILTR	708	Uncharacterized protein OS	UID709		S	1074
167	18.9581	19.2792	17.8526	17.3142	20.2982	18.2199	16.6065	17.6509	0.999194	0.03389	63.181	20.396	63.181	2.7069	4854900	1820	0.0616702	1	0.157179	0.156622	1820	H9JEX4	EDIVEVSDRSESAIVTKVATNLKKREI	SES(0.001)AIVT(0.999)KVAT(0.999)N	1201	Uncharacterized protein OS	UID2992	2	T	707
168	18.9581	19.2792	17.8526	17.3142	20.2982	18.2199	16.6065	17.6509	0.999022	0.03389	63.181	20.396	63.181	2.7069	4854900	1824	0.0616702	1	0.157179	0.156622	1824	H9JEX4	EVSDRSESAIVTKVATNLKKREEKEI\	SES(0.001)AIVT(0.999)KVAT(0.999)N	1202	Uncharacterized protein OS	UID2993	2	T	707
169	25.9603	23.5157	24.0151	22.6229	26.5505	23.7227	19.4904	25.1196	0.999974	0.00550939	87.719	30.494	87.719	-3.5963	588740000	81	0.065183	1	0.307701	0.172958	81	H9JS59	HSLFKSTTASPLRKASSTESGDFVIV	KAS(1)S(1)T(1)ESGDFVIVTPATEIK	650	Uncharacterized protein OS	UID4231	3	S	999
170	25.9603	23.5157	24.0151	22.6229	26.5505	23.7227	19.4904	25.1196	0.999977	0.00550939	87.719	30.494	87.719	-3.5963	588740000	82	0.065183	1	0.307701	0.172958	82	H9JS59	SLFKSTTASPLRKASSTESGDFVIVT	KAS(1)S(1)T(1)ESGDFVIVTPATEIK	651	Uncharacterized protein OS	UID4232	3	S	999
171	25.9603	23.5157	24.0151	22.6229	26.5505	23.7227	19.4904	25.1196	0.999998	0.00550939	87.719	30.494	87.719	-3.5963	588740000	83	0.065183	1	0.307701	0.172958	83	H9JS59	LFKSTTASPLRKASSTESGDFVIVTP/	KAS(1)S(1)T(1)ESGDFVIVTPATEIK	1361	Uncharacterized protein OS	UID4942	3	T	999
172	19.7831	21.8763	NaN	20.0258	22.541	NaN	19.5297	20.3057	1	0.0108204	63.918	10.522	63.918	-0.056635	20105000	2	0.0721715	1	-0.230412	-0.189046	2	H9IWX0	MSSLIIVLFVFAL/	S(1)S(1)LIIVLFVFALAVY(1)K	114	Uncharacterized protein OS	UID3695	3	S	226
173	19.7831	21.8763	NaN	20.0258	22.541	NaN	19.5297	20.3057	1	0.0108204	63.918	10.522	63.918	-0.056635	20105000	3	0.0721715	1	-0.230412	-0.189046	3	H9IWX0	MSSLIIVLFVFALA'	S(1)S(1)LIIVLFVFALAVY(1)K	115	Uncharacterized protein OS	UID3696	3	S	226
174	19.7831	21.8763	NaN	20.0258	22.541	NaN	19.5297	20.3057	1	0.0108204	63.918	10.522	63.918	-0.056635	20105000	16	0.0721715	1	-0.230412	-0.189046	16	H9IWX0	MSSLIIVLFVFALAVYKLLRRKTVRW	S(1)S(1)LIIVLFVFALAVY(1)K	1527	Uncharacterized protein OS	UID5108	3	Y	226
175	23.2109	23.3744	NaN	23.704	24.94	22.5191	22.1043	24.8982	0.88115	0.018551	51.876	17.383	51.876	-1.7228	776770000	17	0.0729161	1	-0.185671	-0.184851	17	H9IZ02	IYPIETVLELQDSELSVFETTIRFVGGI	MIY(0.778)PIET(0.197)VLELQDS(0.04	157	alpha-1,2-Mannosidase OS	UID3738	3	S	279
176	23.2109	23.3744	NaN	23.704	24.94	22.5191	22.1043	24.8982	0.778239	0.018551	51.876	17.383	51.876	-1.7228	776770000	3	0.0729161	1	-0.185671	-0.184851	3	H9IZ02	MIYPIETVLELQDS	MIY(0.778)PIET(0.197)VLELQDS(0.04	1551	alpha-1,2-Mannosidase OS	UID5132	3	Y	279
177	NaN	17.1746	NaN	18.2095	17.0866	NaN	17.14	19.5882	0.914919	0.032977	47.09	18.079	47.09	-0.13683	3593100	2	0.0747527	1	-0.246237	-0.199726	2	Q68HB4	MSWQDYVDKQI	MS(0.915)WQDY(0.005)VDKQLMAS(	824	Profilin OS	UID825	1	S	1230
178	NaN	19.3789	16.5082	17.0272	21.7536	16.264	16.6454	17.4195	1	0.0299747	71.555	40.327	71.555	-3.5288	6015600	4	0.0819302	1	-0.382511	-0.215902	4	A4F5B9	MARYNIVVAVLVLG	MARY(1)NIVVAVLVLGVVGAR	1467	Pheromone binding protein 3 OS	UID1468	1	Y	0
179	21.0189	17.4531	NaN	19.9036	21.5792	18.9488	17.9683	20.6623	1	0.0115144	89.663	10.317	89.663	-0.092481	103990000	112	0.0914618	1	-0.331114	-0.235326	112	H9JQT8	GIRARCFLSVMKFPMSKQKQWLDI	ARCFLSVMKFPMS(1)K	622	Uncharacterized protein OS	UID623	1	S	963
180	25.721	25.2989	24.641	24.9742	26.2042	24.3398	24.9585	24.6526	0.954074	0.00941167	52.402	29.253	52.402	1.562	439110000	121	0.0933185	1	0.119989	0.210855	121	H9JTX7	KMDEEDKICYLLTGMSEKYNTVITA	MDEEDKICY(0.001)LLT(0.025)GMS(	687	CCHC-type domain-containing protein OS	UID4268	3	S	1052
181	NaN	20.9577	20.474	19.7701	24.3739	NaN	18.8235	19.4137	0.976199	0.0302381	117.86	66.132	117.86	3.771	36608000	946	0.093492	1	-0.469745	-0.248083	946;961	H9JQ10	VLEPMLGTDKVKPLISEYREYNTDT	VKPLIS(0.976)EY(0.016)REY(0.008)N	614	DNA topoisomerase 2 OS;DNA topoisomerase 2 OS	UID615	1	S	949
182	18.9104	NaN	17.7756	NaN	NaN	18.4273	17.8798	19.3141	0.996275	0.0142146	89.805	39.321	82.505	-0.63926	4184200	128	0.10119	1	-0.19737	-0.251263	128	H9ITK7	VRYRSLDGQEKLTGNTNTYLEKKNH	LT(0.002)GNT(0.996)NT(0.002)YLEKI	882	Uncharacterized protein OS	UID883		T	148
183	17.2781	17.1185	16.6368	NaN	16.876	17.041	17.225	17.1122	0.999926	0.00823451	81.709	21.552	81.709	3.2726	3163300	205	0.104552	1	-0.0524122	-0.185208	205	H9J3Q0	QIIINKMLELANLTLSISIKVLSATITLI	MLELANLTLS(1)IS(1)IK	243	J domain-containing protein OS	UID2034	2	S	397
184	17.2781	17.1185	16.6368	NaN	16.876	17.041	17.225	17.1122	0.999949	0.00823451	81.709	21.552	81.709	3.2726	3163300	207	0.104552	1	-0.0524122	-0.185208	207	H9J3Q0	IINKMLELANLTLSISIKVLSATITLDD	MLELANLTLS(1)IS(1)IK	244	J domain-containing protein OS	UID2035	2	S	397
185	NaN	20.7406	19.2581	21.3596	19.2564	20.4131	21.0574	22.1205	0.999999	0.0383681	60.972	8.0352	60.972	2.5822	20170000	9	0.107709	0.990747	-0.259104	-0.264284	9	H9J659	MLSAKEMVYRELGNLLEE	LSAKEMVY(1)R	1599	Uncharacterized protein OS	UID1600	1	Y	471
186	18.5218	20.2519	18.8785	18.2364	20.2456	18.9357	17.4146	18.4081	1	0.0356833	42.095	17.302	42.095	3.4776	8020800	377	0.110694	0.99297	0.221128	0.263406	377	H9JIR5	RIAEQRMEIMFQELVSSERRNVQLV	MEIMFQELVS(1)S(1)ERR	479	Uncharacterized protein OS	UID2270	2	S	792
187	18.5218	20.2519	18.8785	18.2364	20.2456	18.9357	17.4146	18.4081	1	0.0356833	42.095	17.302	42.095	3.4776	8020800	378	0.110694	0.996745	0.221128	0.263406	378	H9JIR5	IAEQRMEIMFQELVSSERRNVQLV(	MEIMFQELVS(1)S(1)ERR	480	Uncharacterized protein OS	UID2271	2	S	792
188	21.5319	20.7987	18.081	21.8068	21.1467	18.5202	20.8729	20.4111	0.783268	0.0160574	53.718	9.3764	53.718	-0.408	203030000	625	0.113335	0.999	0.316878	0.278634	625	H9JFM2	KDVIILYKDKNNSTLSNGVVLKHGK'	NNS(0.433)T(0.783)LS(0.783)NGVVL	442	Uncharacterized protein OS	UID4023	3	S	724
189	21.5319	20.7987	18.081	21.8068	21.1467	18.5202	20.8729	20.4111	0.783266	0.0160574	53.718	9.3764	53.718	-0.408	203030000	623	0.113335	1	0.316878	0.278634	623	H9JFM2	NIKDVIILYKDKNNSTLSNGVVLKHG	NNS(0.433)T(0.783)LS(0.783)NGVVL	1221	Uncharacterized protein OS	UID4802	3	T	724
190	NaN	18.3116	17.3942	NaN	NaN	18.1447	17.0898	NaN	0.994618	0.0171738	45.047	14.071	45.047	0.75989	5863500	379	0.11458	0.997374	0.235659	0.294945	379	H9J688	RISWYHNDRPVMQNMSSGIIVSTR	PVMQNMS(0.995)S(0.995)GIIVS(0.4	279	Uncharacterized protein OS	UID3860	3	S	473
191	NaN	18.3116	17.3942	NaN	NaN	18.1447	17.0898	NaN	0.994644	0.0171738	45.047	14.071	45.047	0.75989	5863500	380	0.11458	1	0.235659	0.294945	380	H9J688	ISWYHNDRPVMQNMSSGIIVSTRS	PVMQNMS(0.995)S(0.995)GIIVS(0.4	280	Uncharacterized protein OS	UID3861	3	S	473
192	21.2499	21.6568	22.1017	21.2447	21.5178	21.3838	20.7254	22.1726	1	0.00604038	114.5	51.421	101.65	-0.45815	53058000	1551	0.117225	1	0.113365	0.246495	1551	H9JV29	QPDASGIFISRLIRHTEKLLEEKEEKL(	HT(1)EKLLEEKEEK	1407	Uncharacterized protein OS	UID1408	1	T	1086
193	NaN	17.4952	16.6829	16.8267	NaN	15.8957	17.1086	18.8764	0.999986	0.00680124	57.338	18.342	57.338	-3.9205	2575100	8	0.11797	1	-0.291931	-0.29168	8	H9J6J1	MFNRQQISQIPDASGKC	FNRQQIS(1)QIPDASGKCLATQK	286	Uncharacterized protein OS	UID287	1	S	479
194	18.8487	19.6127	18.9381	18.3365	20.4271	NaN	17.9894	NaN	0.999084	0.0384494	42.947	12.235	42.947	-1.3336	32385000	1068	0.118365	1	-0.274228	-0.290598	1068	H9J557	VSLDELSHPNNPRMFSLQKIVEISYY	MFS(0.999)LQKIVEISYYNMGR	272	SEC7 domain-containing protein OS	UID273		S	446
195	NaN	18.9255	21.3011	21.0885	NaN	19.9693	20.7563	19.7504	1	0.034473	40.589	7.3617	40.589	0.36704	31060000	57	0.125148	1	0.279692	0.304653	57	H9JR30	PAPHSLSSTPKHGARTHPLDFTLTGI	T(1)HPLDFT(1)LT(1)GKQIT(1)RR	1347	Uncharacterized protein OS	UID4928	4	T	969
196	NaN	18.9255	21.3011	21.0885	NaN	19.9693	20.7563	19.7504	1	0.034473	40.589	7.3617	40.589	0.36704	31060000	63	0.125148	1	0.279692	0.304653	63	H9JR30	SSTPKHGARTHPLDFTLTGKQITRRS	T(1)HPLDFT(1)LT(1)GKQIT(1)RR	1348	Uncharacterized protein OS	UID4929	4	T	969
197	NaN	18.9255	21.3011	21.0885	NaN	19.9693	20.7563	19.7504	1	0.034473	40.589	7.3617	40.589	0.36704	31060000	65	0.125148	0.999733	0.279692	0.304653	65	H9JR30	TPKHGARTHPLDFTLTGKQITRRSDI	T(1)HPLDFT(1)LT(1)GKQIT(1)RR	1349	Uncharacterized protein OS	UID4930	4	T	969
198	NaN	18.9255	21.3011	21.0885	NaN	19.9693	20.7563	19.7504	1	0.034473	40.589	7.3617	40.589	0.36704	31060000	70	0.125148	0.995828	0.279692	0.304653	70	H9JR30	ARTHPLDFTLTGKQITRRSDRANSLF	T(1)HPLDFT(1)LT(1)GKQIT(1)RR	1350	Uncharacterized protein OS	UID4931	4	T	969
199	17.1002	NaN	16.1956	18.8207	18.586	16.6891	NaN	17.8342	0.998018	0.00212248	98.156	46.545	98.156	0.22364	2367000	2	0.128202	1	-0.330929	-0.31606	2	H9J6T6	MSSDSDVEELLLI	S(0.998)S(0.998)DS(0.002)DVEELLLL	289	Uncharacterized protein OS	UID2080	2	S	485
200	17.1002	NaN	16.1956	18.8207	18.586	16.6891	NaN	17.8342	0.998018	0.00212248	98.156	46.545	98.156	0.22364	2367000	3	0.128202	1	-0.330929	-0.31606	3	H9J6T6	MSSDSDVEELLLLY	S(0.998)S(0.998)DS(0.002)DVEELLLL	290	Uncharacterized protein OS	UID2081	2	S	485
201	20.0457	17.1466	18.4797	19.2422	NaN	16.5294	19.7651	21.3512	0.79149	0.0376277	55.437	11.624	55.437	-1.0412	22021000	69	0.130116	1	-0.486672	-0.325886	69	H9IT27	ELDLKAGYKVTVIDTSDPDWWKGK	AGY(0.895)KVT(0.105)VIDT(0.208)S(	54	SH3 domain-containing protein OS	UID1845	2	S	134
202	20.0457	17.1466	18.4797	19.2422	NaN	16.5294	19.7651	21.3512	0.895019	0.0376277	55.437	11.624	55.437	-1.0412	22021000	61	0.130116	1	-0.486672	-0.325886	61	H9IT27	NFKSRHADELDLKAGYKVTVIDTSD	AGY(0.895)KVT(0.105)VIDT(0.208)S(	1495	SH3 domain-containing protein OS	UID3286	2	Y	134
203	22.4701	20.5844	22.4899	21.8487	NaN	20.9068	23.3209	22.1422	0.971518	0.00454833	59.154	25.466	59.154	2.1218	35136000	80	0.130189	1	-0.275051	-0.310058	80	H9JAF1	KDYMNQILDDIGSIYTLQDIETRPSI/	DYMNQILDDIGS(0.001)IY(0.1)T(0.97	1130	WD REPEATS REGION domain-containing protein	UID2921	2	T	592
204	22.4701	20.5844	22.4899	21.8487	NaN	20.9068	23.3209	22.1422	0.927079	0.00454833	59.154	25.466	59.154	2.1218	35136000	86	0.130189	1	-0.275051	-0.310058	86	H9JAF1	ILDDIGSIYTLQDIETRPSIAAFRSCKY	DYMNQILDDIGS(0.001)IY(0.1)T(0.97	1131	WD REPEATS REGION domain-containing protein	UID2922	2	T	592
205	NaN	17.4005	NaN	17.497	NaN	16.1462	17.741	20.2801	1	0.0230021	83.948	12.334	83.783	-3.9316	3660100	95	0.141324	1	-0.60702	-0.366916	95	H9JRZ6	GFTENTTNKRNEKKISDAQLLDRKLI	IS(1)DAQLLDRK	643	Fibrinogen C-terminal domain-containing protein C	UID644	1	S	992
206	24.1228	21.2445	21.7668	20.8608	23.279	21.8198	22.5256	21.6327	0.99998	0.0327284	62.658	30.567	62.658	-3.217	241850000	49	0.146047	1	-0.31552	-0.34211	49	H9JSD9	IERRREELRAMVGERYRDLIHAADTI	AMVGERY(1)RDLIHAADTIEDMR	1742	Uncharacterized protein OS	UID1743	1	Y	1003
207	18.8205	18.4057	18.6466	17.5079	NaN	16.8854	18.1884	19.1838	0.815431	0.0345894	53.278	18.408	53.278	-0.6675	5989800	21	0.149607	1	0.259289	0.343373	21	H9JLF1	GHLIFDASAEGVHSTSNSRQKRTM/	LRATGHLIFDASAEGVHS(0.553)T(0.81	530	M20 dimer domain-containing protein OS	UID4111	3	S	855
208	18.8205	18.4057	18.6466	17.5079	NaN	16.8854	18.1884	19.1838	0.81543	0.0345894	53.278	18.408	53.278	-0.6675	5989800	23	0.149607	1	0.259289	0.343373	23	H9JLF1	LIFDASAEGVHSTSNSRQKRTMAST	LRATGHLIFDASAEGVHS(0.553)T(0.81	531	M20 dimer domain-containing protein OS	UID4112	3	S	855
209	18.8205	18.4057	18.6466	17.5079	NaN	16.8854	18.1884	19.1838	0.815435	0.0345894	53.278	18.408	53.278	-0.6675	5989800	20	0.149607	1	0.259289	0.343373	20	H9JLF1	TGHLIFDASAEGVHSTSNSRQKRTM	LRATGHLIFDASAEGVHS(0.553)T(0.81	1288	M20 dimer domain-containing protein OS	UID4869	3	T	855
210	18.7242	21.2285	20.243	20.8024	23.8055	20.1293	18.7698	20.1915	0.963163	0.018872	52.03	19.865	52.03	-2.0684	30434000	394	0.149788	0.998393	-0.474493	-0.362466	394	H9JCD9	RQEIREVLSRNDGKMTFEILQELIYM	NDGKMT(0.963)FEILQELIY(0.037)MI	1164	Uncharacterized protein OS	UID1165	1	T	642
211	15.8723	16.4754	16.8215	17.3132	NaN	16.7255	16.9022	NaN	0.992726	0.0323141	54.524	16.844	54.524	-3.1374	1128800	289	0.15831	0.996488	-0.193232	-0.346549	289	H9JUX8	VKEVAKSVTNNVGANSASSFAQLV	S(0.004)VT(0.004)NNVGANS(0.993)/	715	Vitellogenin domain-containing protein OS	UID2506		S	1081
212	15.8723	16.4754	16.8215	17.3132	NaN	16.7255	16.9022	NaN	0.992776	0.0323141	54.524	16.844	54.524	-3.1374	972430	291	0.15831	1	-0.193232	-0.346549	291	H9JUX8	EVAKSVTNNVGANSASSFAQLVRIL	S(0.004)VT(0.004)NNVGANS(0.993)/	716	Vitellogenin domain-containing protein OS	UID2507		S	1081
213	21.8356	NaN	NaN	21.8676	23.6455	18.8946	NaN	20.6681	0.942081	0.0377954	41.962	11.428	41.962	0.3863	172630000	108	0.16016	0.989562	0.782207	0.413987	108	H9JUB5	ILSVGADPVEMTAEGSNPIMSSIQR	ILS(0.055)VGADPVEMT(0.107)AEGS(	697	Uncharacterized protein OS	UID4278	3	S	1064
214	21.8356	NaN	NaN	21.8676	23.6455	18.8946	NaN	20.6681	0.948046	0.0377954	41.962	11.428	41.962	0.3863	172630000	113	0.16016	0.985333	0.782207	0.413987	113	H9JUB5	ADPVEMTAEGSNPIMSSIQRNPFD	ILS(0.055)VGADPVEMT(0.107)AEGS(	698	Uncharacterized protein OS	UID4279	3	S	1064
215	21.8356	NaN	NaN	21.8676	23.6455	18.8946	NaN	20.6681	0.948016	0.0377954	41.962	11.428	41.962	0.3863	172630000	114	0.16016	0.98114	0.782207	0.413987	114	H9JUB5	DPVEMTAEGSNPIMSSIQRNPFDYI	ILS(0.055)VGADPVEMT(0.107)AEGS(	699	Uncharacterized protein OS	UID4280	3	S	1064
216	19.1738	18.2494	17.3339	17.6068	20.6779	17.6322	17.2899	NaN	0.799303	0.0324483	41.542	13.148	41.542	-1.3395	4991500	820	0.165372	0.977722	-0.442367	-0.394067	820	H9JSN2	GNLTPKSDINISQTSSLAQIRRTCPT	S(0.001)DINIS(0.019)QT(0.837)S(0.3	662	Uncharacterized protein OS	UID2453	2	S	1014
217	19.1738	18.2494	17.3339	17.6068	20.6779	17.6322	17.2899	NaN	0.836707	0.0324483	41.542	13.148	41.542	-1.3395	4991500	818	0.165372	0.981864	-0.442367	-0.394067	818	H9JSN2	VIGNLTPKSDINISQTSSLAQIRRTCF	S(0.001)DINIS(0.019)QT(0.837)S(0.3	1372	Uncharacterized protein OS	UID3163	2	T	1014
218	20.3198	17.0697	18.9956	17.6428	20.551	17.205	19.3206	NaN	1	0.0359406	46.926	16.39	46.926	2.2884	16730000	709	0.167309	0.998557	-0.518585	-0.403107	709	H9JPZ8	IHETDSLRDAVRRLWSVSDSIIFGEK	RLWS(1)VS(1)DS(1)IIFGEKMLR	611	PIPK domain-containing protein OS	UID4192		S	947
219	20.3198	17.0697	18.9956	17.6428	20.551	17.205	19.3206	NaN	1	0.0359406	46.926	16.39	46.926	2.2884	16730000	711	0.167309	0.994234	-0.518585	-0.403107	711	H9JPZ8	ETDSLRDAVRRLWSVSDSIIFGEKM	RLWS(1)VS(1)DS(1)IIFGEKMLR	612	PIPK domain-containing protein OS	UID4193		S	947
220	20.3198	17.0697	18.9956	17.6428	20.551	17.205	19.3206	NaN	1	0.0359406	46.926	16.39	46.926	2.2884	16730000	713	0.167309	0.989948	-0.518585	-0.403107	713	H9JPZ8	DSLRDAVRRLWSVSDSIIFGEKMLR	RLWS(1)VS(1)DS(1)IIFGEKMLR	613	PIPK domain-containing protein OS	UID4194		S	947
221	18.3075	19.8614	17.7243	NaN	NaN	17.4672	NaN	18.8852	0.883817	0.0365204	110.53	11.521	110.53	0.67227	3605300	126	0.171586	1	0.454913	0.421858	126	H9JBB5	EKDIYREKEMFRDKETYREKELYRDK	DKET(0.884)Y(0.116)REK	1149	Protein kinase domain-containing protein OS	UID1150		T	613
222	18.0117	23.1384	19.9215	22.438	16.8956	19.4293	22.228	21.8563	1	0.0377736	49.216	15.627	49.216	-3.0908	55350000	147	0.176589	0.990547	0.775115	0.428729	147	H9JFI6	NAKCLTKDGLIRYLMSDENAPVFLD	Y(1)LMS(1)DENAPVFLDR	441	Phosphoinositide phospholipase C OS	UID2232	2	S	720
223	18.0117	23.1384	19.9215	22.438	16.8956	19.4293	22.228	21.8563	1	0.0377736	49.216	15.627	49.216	-3.0908	55350000	144	0.176589	0.986125	0.775115	0.428729	144	H9JFI6	EAKNAKCLTKDGLIRYLMSDENAPV	Y(1)LMS(1)DENAPVFLDR	1657	Phosphoinositide phospholipase C OS	UID3448	2	Y	720
224	26.4548	22.6946	24.4722	23.2501	25.6849	23.7285	24.7778	NaN	0.934816	0.0366739	45.342	21.022	45.342	-2.0874	950740000	259	0.181164	0.977427	-0.512502	-0.429532	259	Q4W6L7	SGVMKLDKGFLWKMFSTPLYKKLV	MFS(0.935)T(0.369)PLY(0.696)KKLV	823	Cyp302a1 OS	UID4404		S	1221
225	20.2076	20.1195	20.5648	19.9694	20.8045	20.1383	21.054	19.5483	1	0.0264249	55.112	20.357	55.112	2.2576	20437000	4	0.184561	0.990521	-0.170969	-0.369713	4	H9JHC2	MAVSQRLLPLRCVI	MAVS(1)QRLLPLR	458	Uncharacterized protein OS	UID459	1	S	758
226	24.8399	25.566	23.2988	25.5563	24.4454	23.7219	24.9449	28.3465	0.914758	0.0183322	93.909	36.495	56.768	1.8627	1227900000	151	0.186233	0.987784	-0.54942	-0.437586	151	H9J1A0	IHASASKISSDSVEGSLSLPRSAPRPI	ISS(0.004)DS(0.006)VEGS(0.915)LS(	209	Fibronectin type-III domain-containing protein OS	UID210	1	S	336
227	20.6043	19.8346	NaN	20.7647	NaN	NaN	19.9886	21.5119	0.998228	0.0199745	87.149	24.983	87.149	-1.0883	12911000	23	0.192341	0.984391	-0.34906	-0.44868	23	H9J8V2	VPVIKLFKLYLMSKYSKKLVSKCIYIQ	LFKLY(0.976)LMS(0.024)KY(0.002)S(	342	TFIIS N-terminal domain-containing protein OS	UID2133	2	S	555
228	20.6043	19.8346	NaN	20.7647	NaN	NaN	19.9886	21.5119	0.976411	0.0199745	87.149	24.983	87.149	-1.0883	12911000	17	0.192341	0.980035	-0.34906	-0.44868	17	H9J8V2	TMAFLTVPVIKLFKLYLMSKYSKKLV	LFKLY(0.976)LMS(0.024)KY(0.002)S(	1616	TFIIS N-terminal domain-containing protein OS	UID3407	2	Y	555
229	18.3416	21.1687	18.4177	18.4844	21.2356	18.3958	NaN	NaN	0.987565	0.0262278	82.305	27.529	82.305	3.9046	9191900	468	0.203604	0.957633	-0.712554	-0.491152	468	H9JFM3	PLHTSLSFYTGSKTLTWITIRRPVKT(	T(0.013)LT(0.988)WIT(1)IR	1222	ZP domain-containing protein OS	UID3013	2	T	725
230	18.3416	21.1687	18.4177	18.4844	21.2356	18.3958	NaN	NaN	0.999934	0.0262278	82.305	27.529	82.305	3.9046	9191900	471	0.203604	0.95326	-0.712554	-0.491152	471	H9JFM3	TSLSFYTGSKTLTWITIRRPVKTGRIP	T(0.013)LT(0.988)WIT(1)IR	1223	ZP domain-containing protein OS	UID3014	2	T	725
231	NaN	17.9159	17.2636	21.8215	21.2989	17.4302	20.9343	19.7523	1	6.12E-61	253.34	200.41	198.98	0.9207	24347000	65	0.213716	0.956638	-0.853598	-0.509292	65	H9JR42	SGADDNQGRSCLVGGSAESNDED:	SCLVGGS(1)AESNDEDSDDR	627	Uncharacterized protein OS	UID628	1	S	973
232	NaN	19.0946	18.7558	18.756	20.0785	18.4876	17.0677	18.2323	0.985941	0.0341916	49.049	6.7111	49.049	0.94288	5937300	158	0.214809	0.953527	0.402273	0.479205	158	H9IWN7	LMVLGLVKGRTGGVSTTINPLGIDL	T(0.013)GGVS(0.016)T(0.986)T(0.98	914	Uncharacterized protein OS	UID2705	2	T	220
233	NaN	19.0946	18.7558	18.756	20.0785	18.4876	17.0677	18.2323	0.985212	0.0341916	49.049	6.7111	49.049	0.94288	5937300	159	0.214809	0.949213	0.402273	0.479205	159	H9IWN7	MVLGLVKGRTGGVSTTINPLGIDLD	T(0.013)GGVS(0.016)T(0.986)T(0.98	915	Uncharacterized protein OS	UID2706	2	T	220
234	22.3147	22.5267	23.383	22.5863	22.746	22.3897	22.3113	22.7826	0.999984	0.0324609	68.168	12.081	68.168	3.6671	114840000	7	0.220961	1	0.145278	0.399734	7	Q2F5J9	MLTKNITNLFIRNCTLKI	MLTKNIT(1)NLFIR	1456	Mitochondrial thioredoxin 2 OS	UID1457	1	T	1206
235	NaN	19.6882	NaN	16.8873	22.7045	16.6715	21.3998	17.7011	0.932402	0.0185118	67.519	23.728	67.519	-0.18659	11464000	453	0.222988	0.935464	-1.3315	-0.547701	453	H9JCA0	LDGCAYVWDVEKTIISLPLQTGVMI	T(0.068)IIS(0.932)LPLQT(1)GVMLR	389	WD REPEATS REGION domain-containing protein	UID2180	2	S	640
236	22.181	22.8619	23.124	23.5492	21.1467	24.477	23.9307	23.9871	1	0.0034296	108.77	49.349	108.77	-1.0892	189930000	843	0.226921	0.947926	-0.456347	-0.502161	843	H9JHC7	TKGMKVDSTLVPVTDSKSEGNNFD	MTKGMKVDSTLVPVT(1)DS(1)K	459	Serine/threonine-protein kinase TOR OS	UID2250	2	S	759
237	22.181	22.8619	23.124	23.5492	21.1467	24.477	23.9307	23.9871	1	0.0034296	108.77	49.349	101.89	-0.48401	189930000	841	0.226921	0.943558	-0.456347	-0.502161	841	H9JHC7	KMTKGMKVDSTLVPVTDSKSEGNI	MTKGMKVDSTLVPVT(1)DS(1)K	1236	Serine/threonine-protein kinase TOR OS	UID3027	2	T	759
238	16.7748	NaN	18.1753	NaN	NaN	NaN	16.7189	17.2775	1	0.0377838	66.692	37.605	66.692	-2.4038	1799600	1059	0.22789	0.953234	0.476863	0.558455	1059	H9JSM0	IKPDNFLLMKMYMKVSLWDKIFTT	MY(1)MKVS(1)LWDK	661	Uncharacterized protein OS	UID2452		S	1012
239	16.7748	NaN	18.1753	NaN	NaN	NaN	16.7189	17.2775	1	0.0377838	66.692	37.605	66.692	-2.4038	1799600	1055	0.22789	0.949172	0.476863	0.558455	1055	H9JSM0	IHADIKPDNFLLMKMYMKVSLWDI	MY(1)MKVS(1)LWDK	1745	Uncharacterized protein OS	UID3536		Y	1012
240	21.4604	NaN	NaN	20.1118	21.6973	NaN	NaN	20.8798	0.972257	0.00789799	76.533	42.558	76.533	1.7886	68465000	1190	0.229719	0.95266	-0.502414	-0.56545	1190	H9IXB7	NKPRNRQKKGTNVQDSGYLSSDST	KGT(0.074)NVQDS(0.972)GY(0.951)I	121	Uncharacterized protein OS	UID1912	2	S	241
241	21.4604	NaN	NaN	20.1118	21.6973	NaN	NaN	20.8798	0.951174	0.00789799	76.533	42.558	76.533	1.7886	68465000	1192	0.229719	0.957118	-0.502414	-0.56545	1192	H9IXB7	PRNRQKKGTNVQDSGYLSSDSTNS	KGT(0.074)NVQDS(0.972)GY(0.951)I	1536	Uncharacterized protein OS	UID3327	2	Y	241
242	20.6693	NaN	21.485	19.9962	NaN	19.5255	NaN	24.0968	0.945654	0.0355698	42.568	17.355	42.568	0.96496	30484000	11	0.233172	0.940863	-1.09433	-0.578415	11	H9J8G2	MRCYDHYEEYTWQWKRRV(	MRCYDHYEEY(0.054)T(0.946)WQWK	1105	Uncharacterized protein OS	UID1106		T	538
243	24.4014	20.6863	NaN	20.816	21.6327	20.4798	20.6651	22.38	0.832927	0.0258891	50.055	20.523	50.055	-0.52987	271280000	1956	0.23707	0.928212	0.678522	0.54486	1956	H9JPT0	GGDLLSYLRAKREFLSTPDSLTLLDLI	REFLS(0.833)T(0.833)PDS(0.335)LT(	609	Tyrosine-protein kinase receptor OS	UID4190	3	S	943
244	24.4014	20.6863	NaN	20.816	21.6327	20.4798	20.6651	22.38	0.832927	0.0258891	50.055	20.523	50.055	-0.52987	271280000	1957	0.23707	0.932696	0.678522	0.54486	1957	H9JPT0	GDLLSYLRAKREFLSTPDSLTLLDLLN	REFLS(0.833)T(0.833)PDS(0.335)LT(	1331	Tyrosine-protein kinase receptor OS	UID4912	3	T	943
245	24.4014	20.6863	NaN	20.816	21.6327	20.4798	20.6651	22.38	0.997417	0.0258891	50.055	20.523	50.055	-0.52987	271280000	1962	0.23707	0.937223	0.678522	0.54486	1962	H9JPT0	YLRAKREFLSTPDSLTLLDLLNMCVI	REFLS(0.833)T(0.833)PDS(0.335)LT(	1332	Tyrosine-protein kinase receptor OS	UID4913	3	T	943
246	NaN	NaN	17.2993	16.8301	19.3056	16.9295	NaN	16.9275	0.881341	0.0339504	44.662	15.706	44.662	1.5649	1258000	408	0.242529	0.936527	-0.656172	-0.576716	408	H9IWP5	TFSHCFKRAASIAMFSNSNLQRVKF	RAAS(0.116)IAMFS(0.881)NS(0.002)	111	F-box domain-containing protein OS	UID112		S	221
247	NaN	19.6392	18.7606	NaN	22.6567	18.566	NaN	NaN	0.962557	0.000434192	106.67	41.938	79.337	-0.772	10806000	271	0.244577	0.919564	-1.41148	-0.643922	271	H9IS30	KLVYLEAVIKETLRMYSVVPAIARKT	MY(0.963)S(0.875)VVPAIARKT(0.16:	1491	Uncharacterized protein OS	UID3282	2	Y	112
248	22.9511	NaN	19.1654	NaN	18.5743	NaN	NaN	20.6058	0.951287	0.036877	55.907	10.824	55.907	-0.38185	58373000	1157	0.248044	0.912716	1.46819	0.653045	1157	H9JKH8	DRHEIIPRQFERRGKSINLDPSVINSI	S(0.951)INLDPS(0.049)VINSLMSTTET	517	Chitin-binding type-2 domain-containing protein O	UID518	1	S	830
249	19.868	20.5819	20.459	20.5117	19.5381	22.1662	20.295	20.8392	0.947189	0.0261413	58.433	32.303	58.433	0.4126	29056000	426	0.250187	0.941943	-0.354469	-0.522946	426	H9J2K4	LDCVNSKRGSVILKNYGDQTQMLN	NY(0.947)GDQT(0.053)QMLMLK	1573	Uncharacterized protein OS	UID1574		Y	371
250	NaN	20.7445	20.9391	NaN	NaN	NaN	22.564	20.5423	0.999246	0.0360352	47.227	17.924	47.227	0.75592	59588000	87	0.254804	0.921557	-0.711349	-0.637676	87	H9JSZ6	LHGFLPVNIKDVMKKSAKQVMMT	KS(0.999)AKQVMMTEVS(0.001)PK	663	Uncharacterized protein OS	UID664		S	1021
251	19.172	21.0889	18.9123	20.1449	17.7954	NaN	NaN	24.972	1	0.0384055	42.317	9.1697	42.317	-1.1104	58097000	157	0.267897	0.920884	-1.55418	-0.64217	157	H9JT87	GSIRLYGKCVLIDLKSIVVFAYLFLFSI	S(1)IVVFAY(1)LFLFS(1)KER	666	Ig-like domain-containing protein OS	UID4247		S	1027
252	19.172	21.0889	18.9123	20.1449	17.7954	NaN	NaN	24.972	1	0.0384055	42.317	9.1697	42.317	-1.1104	58097000	168	0.267897	0.925757	-1.55418	-0.64217	168	H9JT87	IDLKSIVVFAYLFLFSKERAVSIHSNQ	S(1)IVVFAY(1)LFLFS(1)KER	667	Ig-like domain-containing protein OS	UID4248		S	1027
253	19.172	21.0889	18.9123	20.1449	17.7954	NaN	NaN	24.972	1	0.0384055	42.317	9.1697	42.317	-1.1104	58097000	163	0.267897	0.930681	-1.55418	-0.64217	163	H9JT87	GKCVLIDLKSIVVFAYLFLFSKERAVS	S(1)IVVFAY(1)LFLFS(1)KER	1750	Ig-like domain-containing protein OS	UID5331		Y	1027
254	18.5003	NaN	18.5201	17.917	22.0119	18.1228	17.6494	NaN	1	0.0222501	81.784	6.5712	81.784	-3.5101	8430700	345	0.272446	0.911896	-0.948887	-0.634235	345	H9JM41	LYMDKYRDPKKINEEYLLRKLKNEDI	INEEY(1)LLRK	1696	Uncharacterized protein OS	UID1697	1	Y	870
255	23.3207	21.2523	20.8713	22.907	21.4971	NaN	25.3472	21.9207	0.994647	0.0165445	88.338	24.578	88.338	-2.5626	98997000	20	0.273537	0.908577	-0.83381	-0.619986	20;20;245;466	H9JTG3	TGIFATINGRRLESQSAPAPFDGSGI	RLES(0.049)QS(0.995)APAPFDGS(0.9	267	Uncharacterized protein OS;Uncharacterized prote	UID2058	2	S	432
256	23.3207	21.2523	20.8713	22.907	21.4971	NaN	25.3472	21.9207	0.977531	0.0165445	88.338	24.578	78.692	-2.6906	98997000	28	0.273537	0.903918	-0.83381	-0.619986	28;28;253;474	H9JTG3	GRRLESQSAPAPFDGSGHRYTCNVI	RLES(0.045)QS(0.978)APAPFDGS(0.9	268	Uncharacterized protein OS;Uncharacterized prote	UID2059	2	S	432
257	20.62	NaN	NaN	19.592	21.0522	NaN	20.2005	NaN	0.764621	0.0362333	42.118	12.2	42.118	-4.2167	15694000	104	0.286295	0.893186	-0.520327	-0.67798	104	H9JC68	GMPRLEKMYAVFVPNSKYGSSSDV	LEKMY(0.986)AVFVPNS(0.765)KY(0.(	383	Uncharacterized protein OS	UID2174	2	S	638
258	20.62	NaN	NaN	19.592	21.0522	NaN	20.2005	NaN	0.986062	0.0362333	42.118	12.2	42.118	-4.2167	15694000	97	0.286295	0.88896	-0.520327	-0.67798	97	H9JC68	ALEVCAFGMPRLEKMYAVFVPNSK	LEKMY(0.986)AVFVPNS(0.765)KY(0.(	1634	Uncharacterized protein OS	UID3425	2	Y	638
259	16.5074	18.862	15.5998	16.6809	19.9237	NaN	NaN	16.1212	1	0.0195909	65.252	6.2714	65.252	1.8804	2502300	5	0.287403	0.92741	-1.10996	-0.668848	5	H9J3X9	MEVDSGLIKRDKYSII	EVDS(1)GLIKR	246	Dolichol-phosphate mannosyltransferase subunit 1	UID247	1	S	399
260	19.5405	20.7153	19.6872	19.0982	23.5412	NaN	18.1027	20.5271	0.956755	0.00143354	79.92	31.072	78.661	-2.1212	21303000	190	0.287896	0.915935	-0.963363	-0.652274	190	H9JK79	EAKRRRSITIGTQTMSIVINTIDKETR	RRS(0.039)IT(0.039)IGT(0.038)QT(0.	509	Uncharacterized protein OS	UID4090	3	S	824

261	15.7333	18.9982	16.1614	16.5866	19.3072	16.4798	NaN	NaN	0.999975	0.0310818	66.538	17.114	66.538	2.6275	2245700	371	0.297413	0.96446	-1.02366	-0.684049	371	H9JCG3	KLLSELAEHDDKKIESVLHDAITARLE	KIES(1)VLHDAITAR	390	Uncharacterized protein OS	UID391	1	S	643
262	23.1484	21.5158	21.8072	23.0027	NaN	22.4102	NaN	23.3265	0.974278	0.00805761	93.839	39.353	93.839	-0.50725	119440000	694	0.297924	0.916356	-0.499842	-0.640105	694	H9J4H5	LDGRSPSPGKGSVLVSPGSTSSGFS	GS(0.003)VLVS(0.974)PGS(0.011)T(	254	Uncharacterized protein OS	UID2045	2	S	420
263	23.1484	21.5158	21.8072	23.0027	NaN	22.4102	NaN	23.3265	0.978038	0.00805761	93.839	39.353	80.871	-1.2651	119440000	703	0.297924	0.911583	-0.499842	-0.640105	703	H9J4H5	KGSVLVSPGSTSSGFSSRRSSQDGF	GS(0.025)VLVS(0.965)PGS(0.007)T(	255	Uncharacterized protein OS	UID2046	2	S	420
264	22.3058	24.0889	23.4948	23.1401	16.4663	24.8427	23.2062	23.1219	0.845103	0.0292962	81.784	49.429	81.784	-1.4391	127800000	426	0.298143	0.97519	1.34813	0.676066	426	H9JCU5	KKTLETLNKLSPLTEYRKMKQEATK	LSPLT(0.155)EY(0.845)R	1640	SAM domain-containing protein OS	UID1641	1	Y	652
265	20.9429	NaN	NaN	22.0106	22.5807	20.3926	22.897	22.8815	0.908224	0.0321172	43.658	13.066	43.658	1.483	39559000	489	0.300527	0.94724	-0.711212	-0.669959	489	H9J0V7	SAVITDMAAPLQLTVSTAASVLEAQ	QS(0.124)AVIT(0.879)DMAAPLQLT(	196	PIEZO domain-containing protein OS	UID3777	4	S	325
266	20.9429	NaN	NaN	22.0106	22.5807	20.3926	22.897	22.8815	0.87866	0.0321172	43.658	13.066	43.658	1.483	39559000	478	0.300527	0.931626	-0.711212	-0.669959	478	H9J0V7	QRVQEIRQRRQSAVITDMAAPLQLT	QS(0.124)AVIT(0.879)DMAAPLQLT(	979	PIEZO domain-containing protein OS	UID4560	4	T	325
267	20.9429	NaN	NaN	22.0106	22.5807	20.3926	22.897	22.8815	0.995477	0.0321172	43.658	13.066	43.658	1.483	39559000	487	0.300527	0.936773	-0.711212	-0.669959	487	H9J0V7	RQSAVITDMAAPLQLTVSTAASVLE	QS(0.124)AVIT(0.879)DMAAPLQLT(	980	PIEZO domain-containing protein OS	UID4561	4	T	325
268	20.9429	NaN	NaN	22.0106	22.5807	20.3926	22.897	22.8815	0.908221	0.0321172	43.658	13.066	43.658	1.483	39559000	490	0.300527	0.941978	-0.711212	-0.669959	490	H9J0V7	AVITDMAAPLQLTVSTAASVLEAQR	QS(0.124)AVIT(0.879)DMAAPLQLT(	981	PIEZO domain-containing protein OS	UID4562	4	T	325
269	18.5805	20.156	18.8856	18.3693	22.5636	19.3821	18.2132	18.8671	1	0.0265899	77.318	8.4714	77.318	3.2614	15576000	265	0.305087	0.954114	-0.758668	-0.662332	265	H9IV22	VREKIGCQESVRLLGSLKHSEIRNVL	LLGS(1)LKHS(1)EIR	81	Uncharacterized protein OS	UID1872	2	S	177
270	18.5805	20.156	18.8856	18.3693	22.5636	19.3821	18.2132	18.8671	1	0.0265899	77.318	8.4714	77.318	3.2614	15576000	269	0.305087	0.959566	-0.758668	-0.662332	269	H9IV22	IGCQESVRLLGSLKHSEIRNVLVKGD	LLGS(1)LKHS(1)EIR	82	Uncharacterized protein OS	UID1873	2	S	177
271	19.0293	19.4083	18.2371	18.3526	20.9396	19.1116	17.9772	NaN	0.864661	0.0361003	47.752	11.407	47.752	-2.3974	13120000	120	0.30668	0.923174	-0.585978	-0.655642	120	H9JDM6	LYRPDRDSKEEENERSFVMSGSLTD	S(0.865)FVMS(0.186)GS(0.84)LT(0.0	417	Homeobox domain-containing protein OS	UID2208		S	674
272	19.0293	19.4083	18.2371	18.3526	20.9396	19.1116	17.9772	NaN	0.840405	0.0361003	47.752	11.407	47.752	-2.3974	13120000	126	0.30668	0.918184	-0.585978	-0.655642	126	H9JDM6	DSKEEENERSFVMSGSLTDSIYVRSI	S(0.865)FVMS(0.186)GS(0.84)LT(0.0	418	Homeobox domain-containing protein OS	UID2209		S	674
273	NaN	20.3782	17.9197	20.1274	27.1412	18.818	19.0894	20.0568	0.999999	0.0108865	126.31	41.815	126.31	-0.95757	162650000	460	0.30903	0.968415	-1.80125	-0.713602	460	H9J3M1	SGLVPTHQKLETSRAYLVARSLGAE	LETSRAY(1)LVAR	1579	Uncharacterized protein OS	UID1580	1	Y	394
274	19.7568	20.3192	NaN	18.844	19.7591	18.2669	NaN	NaN	0.797823	0.0144685	70.525	21.866	70.525	-1.9779	9017700	3	0.315937	0.964578	0.627003	0.708012	3	H9JHH4	MRTKGSLYRTDVV	MRT(0.798)KGS(0.203)LY(0.999)R	1237	Uncharacterized protein OS	UID3028	2	T	762
275	19.7568	20.3192	NaN	18.844	19.7591	18.2669	NaN	NaN	0.999249	0.0144685	70.525	21.866	70.525	-1.9779	9017700	8	0.315937	0.970186	0.627003	0.708012	8	H9JHH4	MRTKGSLYRTDVVLSLLG	MRT(0.798)KGS(0.203)LY(0.999)R	1665	Uncharacterized protein OS	UID3456	2	Y	762
276	21.8421	22.0842	20.809	20.4471	21.8321	20.4394	22.7459	22.0596	0.999833	0.0260411	64.798	8.659	64.798	-1.5142	108680000	3	0.321233	0.955706	-0.473641	-0.653273	3	H9JTL9	MLTVEPYLTEPFG	MLT(1)VEPY(0.977)LT(0.001)EPFGS(	1386	Uncharacterized protein OS	UID3177	2	T	1041
277	21.8421	22.0842	20.809	20.4471	21.8321	20.4394	22.7459	22.0596	0.976596	0.0260411	64.798	8.659	64.798	-1.5142	108680000	7	0.321233	0.950337	-0.473641	-0.653273	7	H9JTL9	MLTVEPYLTEPFGSRGS	MLT(1)VEPY(0.977)LT(0.001)EPFGS(	1753	Uncharacterized protein OS	UID3544	2	Y	1041
278	NaN	22.4983	20.7792	23.2542	22.9538	20.497	20.6406	21.8064	1	0.0337205	46.324	16.691	46.324	2.6139	85440000	34	0.321625	0.969725	0.702808	0.692836	34	H9JQP4	EGAPDGRLARKQQRPTRGFKNVEN	PT(1)RGFKNVEMMT(1)AR	1338	Uncharacterized protein OS	UID3129		T	960
279	NaN	22.4983	20.7792	23.2542	22.9538	20.497	20.6406	21.8064	1	0.0337205	46.324	16.691	46.324	2.6139	85440000	44	0.321625	0.975566	0.702808	0.692836	44	H9JQP4	KQQRPTRGFKNVEMMTARGFGKR	PT(1)RGFKNVEMMT(1)AR	1339	Uncharacterized protein OS	UID3130		T	960
280	20.7949	21.8378	20.4588	20.0871	24.0557	22.12	19.7865	20.3867	1	0.0342953	97.203	24.305	97.203	-0.87405	45938000	2392	0.326687	0.965212	-0.792598	-0.700616	2392	H9J2W5	RYLEKARLEPNVELETKNIRTKRDFA	ARLEPNVELET(1)K	1014	Uncharacterized protein OS	UID1015	1	T	381
281	17.3351	16.8825	16.7895	17.33	18.2982	16.7688	16.7644	17.7954	1	0.0270751	73.067	35.113	73.067	-1.5904	2725100	13	0.335841	0.928278	-0.322404	-0.632276	13	H9IU05	MLEGFVCKMVQQSKKKDILKE	LEGFVCKMVQQS(1)KK	71	Uncharacterized protein OS	UID72	1	S	159
282	26.3175	24.5504	25.6867	25.4529	NaN	24.3663	25.6017	25.2564	0.990479	0.00163538	111.5	33.756	111.5	-0.40916	1033600000	307	0.339123	0.984213	0.427073	0.676472	307	H9JN60	PPFTDLAGGPKKSQTSTTGFIISVYK	KS(0.013)QT(0.987)S(0.99)T(0.003)T	563	Uncharacterized protein OS	UID4144	3	S	896
283	26.3175	24.5504	25.6867	25.4529	NaN	24.3663	25.6017	25.2564	0.987363	0.00163538	111.5	33.756	111.5	-0.40916	1068500000	306	0.339123	0.972702	0.427073	0.676472	306	H9JN60	RPPFTDLAGGPKKSQTSTTGFIISVY	KS(0.013)QT(0.987)S(0.99)T(0.003)T	1309	Uncharacterized protein OS	UID4890	3	T	896
284	26.3175	24.5504	25.6867	25.4529	NaN	24.3663	25.6017	25.2564	0.999942	0.00163538	111.5	33.756	111.5	-0.40916	1136800000	316	0.339123	0.978424	0.427073	0.676472	316	H9JN60	PKKSQTSTTGFIISVYKVFEGDDGER	KS(0.013)QT(0.987)S(0.99)T(0.003)T	1706	Uncharacterized protein OS	UID5287	3	Y	896
285	20.1984	NaN	18.7324	17.5946	21.6811	NaN	18.5949	NaN	0.760424	0.0366542	41.485	13.441	41.485	-4.1274	20826000	39	0.344048	0.942304	-1.29616	-0.806824	39	H9J5W6	DVSAAAFRIQSEIIKTPCVKSHMSSII	T(0.76)PCVKS(0.239)HMS(0.004)S(0	1065	Uncharacterized protein OS	UID2856		T	468
286	20.1984	NaN	18.7324	17.5946	21.6811	NaN	18.5949	NaN	0.977373	0.0366542	41.485	13.441	41.485	-4.1274	20826000	55	0.344048	0.948306	-1.29616	-0.806824	55	H9J5W6	PCVKSHMSSIFGMDIYLKNDFLQH	T(0.76)PCVKS(0.239)HMS(0.004)S(0	1598	Uncharacterized protein OS	UID3389		Y	468
287	18.1201	19.687	NaN	19.6925	23.7248	18.9988	18.9272	19.7227	0.776748	0.00143354	79.92	31.072	79.92	-2.4808	42090000	186	0.351751	0.978517	-1.17684	-0.774407	186	H9JK79	VDVIEAKRRRSITIGTQTMSIVINTID	RRS(0.06)IT(0.06)IGT(0.777)QT(0.32	1268	Uncharacterized protein OS	UID4849	3	T	824
288	18.1201	19.687	NaN	19.6925	23.7248	18.9988	18.9272	19.7227	1	0.00143354	79.92	31.072	79.92	-2.4808	43811000	195	0.351751	0.972079	-1.17684	-0.774407	195	H9JK79	RSITIGTQTMSIVINTIDKETRIIETDK	RRS(0.06)IT(0.06)IGT(0.777)QT(0.32	1270	Uncharacterized protein OS	UID4851	3	T	824
289	NaN	18.6781	18.2194	17.4897	22.579	18.8605	18.1406	17.5124	0.998317	0.0171739	83.53	7.8485	83.53	1.264	8841000	817	0.353354	1	-1.14402	-0.775713	817	H9J469	DQKQTTPDLLVNGKTSNAPILTTGT	T(0.998)S(0.998)NAPILT(0.001)T(0.0	250	ANK REP REGION domain-containing protein OS	UID3831	3	S	405
290	NaN	18.6781	18.2194	17.4897	22.579	18.8605	18.1406	17.5124	0.998203	0.0171739	83.53	7.8485	83.53	1.264	8841000	816	0.353354	0.997081	-1.14402	-0.775713	816	H9J469	SDQKQTTPDLLVNGKTSNAPILTTG	T(0.998)S(0.998)NAPILT(0.001)T(0.0	1025	ANK REP REGION domain-containing protein OS	UID4606	3	T	405
291	20.1267	20.1326	19.0228	18.2392	24.4043	20.2053	18.9281	18.6515	0.806349	0.0249607	59.116	15.573	58.04	1.697	28644000	4	0.35786	1	-1.16698	-0.774281	4	Q2F5L5	MPQSPSATSVGSG	MPQS(0.806)PS(0.498)AT(0.68)S(0.(	814	Protein transport protein Sec61 subunit beta OS	UID4395	3	S	1207
292	20.1267	20.1326	19.0228	18.2392	24.4043	20.2053	18.9281	18.6515	0.751831	0.0249607	59.116	15.573	59.116	-0.67397	28644000	8	0.35786	1	-1.16698	-0.774281	8	Q2F5L5	MPQSPSATSVGSGSRSP	MPQS(0.617)PS(0.617)AT(0.752)S(0	1457	Protein transport protein Sec61 subunit beta OS	UID5038	3	T	1207
293	NaN	NaN	20.1097	20.8077	NaN	17.809	19.4441	20.9321	1	0.0166944	89.698	38.352	62.466	2.2988	20305000	6	0.358201	1	1.06362	0.822579	6	H9IZP2	MILQRTIGQDRAIGGC	MILQRT(1)IGQDR	964	Uncharacterized protein OS	UID965	1	T	303
294	NaN	17.4474	NaN	17.8579	17.7791	17.121	19.1036	19.6166	0.998579	0.0267606	54.501	13.981	43.024	0.047392	4234800	368	0.358321	0.922247	-0.752389	-0.771903	368	H9J2R9	HDKHEMQKMQKDLEHYKSEIAELS	MQKDLEHY(0.999)KS(0.999)EIAELS(	1574	CAP-Gly domain-containing protein OS	UID3365	2	Y	376
295	19.4222	22.2021	21.7459	21.0505	23.8984	21.2047	21.5005	20.9076	0.949812	0.0178942	44.788	24.796	44.788	0.36511	54831000	66	0.365226	0.969882	-0.772626	-0.760463	66	H9J7I7	GIRKERFSTFTVVTLSLFVGTVILVCK	ERFS(0.081)T(0.875)FT(0.27)VVT(0.8	312	Uncharacterized protein OS	UID3893	4	S	504
296	19.4222	22.2021	21.7459	21.0505	23.8984	21.2047	21.5005	20.9076	0.874641	0.0178942	44.788	24.796	44.788	0.36511	54831000	59	0.365226	0.951231	-0.772626	-0.760463	59	H9J7I7	AFLVVFPGIRKERFSTFTVVTLSLFV(	ERFS(0.081)T(0.875)FT(0.27)VVT(0.8	1087	Uncharacterized protein OS	UID4668	4	T	504
297	19.4222	22.2021	21.7459	21.0505	23.8984	21.2047	21.5005	20.9076	0.826781	0.0178942	44.788	24.796	44.788	0.36511	54831000	64	0.365226	0.963584	-0.772626	-0.760463	64	H9J7I7	FPGIRKERFSTFTVVTLSLFVGTVILV	ERFS(0.081)T(0.875)FT(0.27)VVT(0.8	1088	Uncharacterized protein OS	UID4669	4	T	504
298	19.4222	22.2021	21.7459	21.0505	23.8984	21.2047	21.5005	20.9076	0.997191	0.0178942	44.788	24.796	44.788	0.36511	54831000	71	0.365226	0.957368	-0.772626	-0.760463	71	H9J7I7	RFSTFTVVTLSLFVGTVILVCKHGSS'	ERFS(0.081)T(0.875)FT(0.27)VVT(0.8	1089	Uncharacterized protein OS	UID4670	4	T	504
299	25.8578	24.4138	23.8088	NaN	26.1214	NaN	NaN	24.9154	0.78574	0.03538	51.566	24.812	51.566	-1.754	857580000	8	0.367667	1	-0.824939	-0.821456	8	H9JL15	MLKISGLTVCCFVLSADS	MLKIS(0.214)GLT(0.786)VCCFVLSAD	1282	SERPIN domain-containing protein OS	UID1283		T	842
300	17.629	21.2866	19.8818	19.3994	22.1512	20.4028	18.3474	21.092	1	0.0284305	100.82	44.187	100.82	-1.2437	19828000	1609	0.374748	1	-0.949156	-0.789995	1609	H9J4V7	EEYEFRVKAINDEGESEPLKTDHSIIA	VKAINDEGES(1)EPLK	269	Uncharacterized protein OS	UID270		S	433
301	24.7234	23.3598	NaN	17.5159	19.452	21.5206	21.2537	17.834	0.901018	0.0210974	63.037	13.049	63.037	3.8616	412670000	132	0.376119	1	1.85132	0.83735	132;139	Q9U6V7	EGDGSKPSGPSSLGPSNPATRRASE	DRMLEGDGS(0.002)KPS(0.083)GPS(	537	J domain-containing protein OS;J domain-containir	UID2328	2	S	859
302	25.3763	NaN	23.8191	22.984	NaN	22.375	23.7801	NaN	0.972343	0.0384748	70.117	36.487	70.117	3.3128	178790000	32	0.380082	1	0.982294	0.857471	32	H9IUS9	LCRSLVSIESQKTPQSGASSDGISPP	T(0.974)PQS(0.972)GAS(0.03)S(0.02	79	Uncharacterized protein OS	UID1870		S	171
303	25.3763	NaN	23.8191	22.984	NaN	22.375	23.7801	NaN	0.973646	0.0384748	70.117	36.487	70.117	3.3128	178790000	29	0.380082	1	0.982294	0.857471	29	H9IUS9	IKCLCRSLVSIESQKTPQSGASSDGIS	T(0.974)PQS(0.972)GAS(0.03)S(0.02	897	Uncharacterized protein OS	UID2688		T	171
304	19.3704	17.8551	18.8494	16.8096	NaN	19.1982	NaN	18.8007	0.808666	0.0194944	54.964	31.628	54.964	0.41587	4914600	2	0.381573	1	-0.77829	-0.812919	2	H9JKT6	MTQIASKYKFTN'	T(0.809)QIAS(0.292)KY(0.894)KFT(0.	1276	CHK domain-containing protein OS	UID4857	3	T	837
305	19.3704	17.8551	18.8494	16.8096	NaN	19.1982	NaN	18.8007	0.914537	0.0194944	54.964	31.628	40.187	-0.048932	4914600	8	0.381573	1	-0.77829	-0.812919	8	H9JKT6	MTQIASKYKFTNWQYSR	T(0.517)QIAS(0.517)KY(0.915)KFT(0.	1689	CHK domain-containing protein OS	UID5270	3	Y	837
306	19.3704	17.8551	18.8494	16.8096	NaN	19.1982	NaN	18.8007	0.866856	0.0194944	54.964	31.628	54.964	0.41587	4914600	15	0.381573	1	-0.77829	-0.812919	15	H9JKT6	MTQIASKYKFTNWQYSRDKFECIA	T(0.809)QIAS(0.292)KY(0.894)KFT(0.	1690	CHK domain-containing protein OS	UID5271	3	Y	837
307	20.1243	19.3251	NaN	18.1078	23.2528	NaN	18.6524	NaN	0.804605	0.0229296	69.188	24.12	69.188	2.6765	12489000	30	0.381599	1	-1.76688	-0.895039	30	H9JVW9	TVSPLHDYFVTWGKMYRSSQYTTN	MY(0.805)RS(0.325)S(0.408)QY(0.48	1767	Uncharacterized protein OS	UID5348		Y	1101
308	20.6775	20.6694	20.6129	21.0094	18.9825	20.7991	20.5188	21.0186	0.999993	0.0288799	68.168	16.704	68.168	-1.6939	24344000	2	0.383821	0.947828	0.412549	0.724607	2	H9J3B3	MTPPMGWLTW	T(1)PPMGWLTWERFR	1023	Alpha-galactosidase OS	UID1024	1	T	391
309	16.1519	NaN	15.4592	17.1228	NaN	16.0076	NaN	18.7537	1	0.0350989	54.094	15.026	54.094	-0.78219	1895500	31	0.384085	1	-1.13603	-0.875182	31	H9JWP7	DPKTGTRGTDRVLKISRLEAPDMVM	IS(1)RLEAPDMVMR	744	CCHC-type domain-containing protein OS	UID745		S	1117
310	22.6189	23.6412	23.2667	23.3799	22.8865	21.2499	23.3488	23.4595	0.80419	0.0322159	42.185	8.7331	42.185	-1.5898	268940000	227	0.38634	0.939346	0.490512	0.748851	227	H9IY87	ESLKFIQNKYIDTIDSDLLSLSELWGI	Y(0.982)IDT(0.279)IDS(0.804)DLLS(0	136	Uncharacterized protein OS	UID3717	3	S	262
311	22.6189	23.6412	23.2667	23.3799	22.8865	21.2499	23.3488	23.4595	0.840738	0.0322159	42.185	8.7331	42.185	-1.5898	268940000	231	0.38634	0.933475	0.490512	0.748851	231	H9IY87	FIQNKYIDTIDSDLLSLSELWGDKAE	Y(0.982)IDT(0.279)IDS(0.804)DLLS(0	137	Uncharacterized protein OS	UID3718	3	S	262
312	22.6189	23.6412	23.2667	23.3799	22.8865	21.2499	23.3488	23.4595	0.982106	0.0322159	42.185	8.7331	42.185	-1.5898	268940000	221	0.38634	0.927677	0.490512	0.748851	221	H9IY87	PFGENRESLKFIQNKYIDTIDSDLLSL	Y(0.982)IDT(0.279)IDS(0.804)DLLS(0	1544	Uncharacterized protein OS	UID5125	3	Y	262
313	17.3734	NaN	15.3236	17.7844	17.8631	16.6552	NaN	18.5683	1	0.000501402	129	50.489	129	-3.545	5167100	807	0.387562	1	-0.868454	-0.831806	807	H9JYA6	TVSPSQPKVTVAKSKSEKELENLKTF	S(1)EKELENLKTR	773	Uncharacterized protein OS	UID774	1	S	1160
314	21.5097	22.3044	20.3136	20.2247	25.072	20.1945	NaN	NaN	0.999931	0.0294124	54.611	6.7426	54.611	-2.5896	68962000	2	0.389823	1	-1.54515	-0.872315	2	H9JTG4	MSRPQQTRVTYS	MS(1)RPQQTR	670	Uncharacterized protein OS	UID671		S	1034
315	17.9813	19.6311	21.0099	18.5918	NaN	18.703	NaN	18.002	0.976061	0.0377778	40.142	20.892	40.142	-3.5495	4475400	379	0.394929	1	0.951038	0.851194	379	H9J9K9	DFSHRELLFGLTTTESYLEFSAQDLE	ELLFGLT(0.994)T(0.977)T(0.977)ES(	351	COesterase domain-containing protein OS	UID3932	5	S	568
316	17.9813	19.6311	21.0099	18.5918	NaN	18.703	NaN	18.002	0.993705	0.0377778	40.142	20.892	40.142	-3.5495	4475400	375	0.394929	1	0.951038	0.851194	375	H9J9K9	YELADFSHRELLFGLTTTESYLEFSAC	ELLFGLT(0.994)T(0.977)T(0.977)ES(	1118	COesterase domain-containing protein OS	UID4699	5	T	568
317	17.9813	19.6311	21.0099	18.5918	NaN	18.703	NaN	18.002	0.97692	0.0377778	40.142	20.892	40.142	-3.5495	4475400	376	0.394929	1	0.951038	0.851194	376	H9J9K9	ELADFSHRELLFGLTTTESYLEFSAQI	ELLFGLT(0.994)T(0.977)T(0.977)ES(	1119	COesterase domain-containing protein OS	UID4700	5	T	568
318	17.9813	19.6311	21.0099	18.5918	NaN	18.703	NaN	18.002	0.97692	0.0377778	40.142	20.892	40.142	-3.5495	4475400	377	0.394929	1	0.951038	0.851194	377	H9J9K9	LADFSHRELLFGLTTTESYLEFSAQD	ELLFGLT(0.994)T(0.977)T(0.977)ES(	1120	COesterase domain-containing protein OS	UID4701	5	T	568
319	17.9813	19.6311	21.0099	18.5918	NaN	18.703	NaN	18.002	0.975109	0.0377778	40.142	20.892	40.142	-3.5495	4475400	380	0.394929	1	0.951038	0.851194	380	H9J9K9	FSHRELLFGLTTTESYLEFSAQDLEF(	ELLFGLT(0.994)T(0.977)T(0.977)ES(	1621	COesterase domain-containing protein OS	UID5202	5	Y	568
320	NaN	19.6579	18.1996	NaN	20.4865	18.4337	19.8525	19.91	0.971479	0.0372335	54.974	28.742	54.974	2.1286	27735000	305	0.395566	1	-0.741879	-0.831262	305	H9IUZ9	VRVEAAAAALRLHWRSDVNSRQTR	S(0.971)DVNS(0.351)RQT(0.724)RY(	80	Uncharacterized protein OS	UID3661		S	174
321	NaN	19.6579	18.1996	NaN	20.4865	18.4337	19.8525	19.91	0.954238	0.0372335	54.974	28.742	54.974	2.1286	27735000	314	0.395566	1	-0.741879	-0.831262	314	H9IUZ9	LRLHWRSDVNSRQTRYVLRYRARVI	S(0.971)DVNS(0.351)RQT(0.724)RY(	1509	Uncharacterized protein OS	UID5090		Y	174
322	17.8811	17.5757	16.6766	18.0092	NaN	17.2431	18.964	17.91	0.996935	0.0382906	44.616	10.437	44.616	0.53895	4540000	153	0.39608	0.991356	-0.503371	-0.77493	153	H9IWI4	SEQWKDISYFEMNLETWRQLWRV	DIS(0.058)Y(0.945)FEMNLET(0.997)'	913	CP-type G domain-containing protein OS	UID2704	2	T	216
323	17.8811	17.5757	16.6766	18.0092	NaN	17.2431	18.964	17.91	0.945047	0.0382906	44.616	10.437	44.616	0.53895	4540000	146	0.39608	0.984747	-0.503371	-0.77493	146	H9IWI4	YIDKLQASEQWKDISYFEMNLETWI	DIS(0.058)Y(0.945)FEMNLET(0.997)'	1525	CP-type G domain-containing protein OS	UID3316	2	Y	216
324	18.3605	18.0967	18.7441	18.3425	18.9198	18.6623	18.419	18.2631	1	0.0178365	83	20.534	83	2.6859	9272400	636	0.404209	0.903224	-0.180079	-0.607816	636	H9J241	TAANGSPEDVRKMLETLTALRYCKP	KMLET(1)LT(1)ALRY(1)CK	1001	Uncharacterized protein OS	UID4582	3	T	360
325	18.3605	18.0967	18.7441	18.3425	18.9198	18.6623	18.419	18.2631	1	0.0178365	83	20.534	83	2.6859	9272400	638	0.404209	0.89864	-0.180079	-0.607816	638	H9J241	ANGSPEDVRKMLETLTALRYCKPNI	KMLET(1)LT(1)ALRY(1)CK	1002	Uncharacterized protein OS	UID4583	3	T	360
326	18.3605	18.0967	18.7441	18.3425	18.9198	18.6623	18.419	18.2631	1	0.0178365	83	20.534	83	2.6859	9272400	642	0.404209	0.894101	-0.180079	-0.607816	642	H9J241	PEDVRKMLETLTALRYCKPNNVLLG	KMLET(1)LT(1)ALRY(1)CK	1572	Uncharacterized protein OS	UID5153	3	Y	360
327	23.3917	20.8239	NaN	21.7384	NaN	20.911	NaN	21.0979	0.9432	0.00200637	89.604	47.699	89.604	1.4763	104240000	537	0.411435	1	0.980201	0.913899	537	H9JM06	AEFDSISSILKNKAKTLNLHVTNRLQV	T(0.943)LNHVT(0.057)NRLQVPSS(0.(	1297	FERM domain-containing protein OS	UID3088	2	T	868
328	19.5459	19.1982	20.0368	20.1158	19.608	19.1662	20.88	21.3984	0.887423	0.0341154	45.07	12.046	45.07	0.58039	20711000	14	0.421669	1	-0.53897	-0.807737	14	H9JPP9	MSALKGNLLVRSLSTSVASAQM'	MS(0.021)ALKGNLLVRS(0.104)LS(0.8	603	Uncharacterized protein OS	UID4184	3	S	939
329	19.5459	19.1982	20.0368	20.1158	19.608	19.1662	20.88	21.3984	0.875505	0.0341154	45.07	12.046	45.07	0.58039	20711000	16	0.421669	1	-0.53897	-0.807737	16	H9JPP9	MSALKGNLLVRSLSTSVASAQMVK	MS(0.021)ALKGNLLVRS(0.104)LS(0.8	604	Uncharacterized protein OS	UID4185	3	S	939
330	19.5459	19.1982	20.0368	20.1158	19.608	19.1662	20.88	21.3984	0.983766	0.0341154	45.07	12.046	45.07	0.58039	20711000	15	0.421669	1	-0.53897	-0.807737	15	H9JPP9	MSALKGNLLVRSLSTSVASAQMV	MS(0.021)ALKGNLLVRS(0.104)LS(0.8	1329	Uncharacterized protein OS	UID4910	3	T	939
331	18.1616	20.257	19.281	19.7291	24.7989	18.2958	19.0359	21.2252	1	0.0220325	63.32	19.021	63.32	1.4058	44893000	7	0.433042	1	-1.48178	-0.911487	7	H9IXV6	MPKNITSEVREIILKVKE	PKNIT(1)S(1)EVREIILK	134	Uncharacterized protein OS	UID1925	2	S	257
332	18.1616	20.257	19.281	19.7291	24.7989	18.2958	19.0359	21.2252	1	0.0220325	63.32	19.021	63.32	1.4058	44893000	6	0.433042	1	-1.48178	-0.911487	6	H9IXV6	MPKNITSEVREIILKVK	PKNIT(1)S(1)EVREIILK	933	Uncharacterized protein OS	UID2724	2	T	257
333	22.5341	22.7679	22.8117	22.8176	24.8436	21.9043	22.9249	23.6958	0.936486	0.0243858	95.755	48.538	57.794	-3.0534	394850000	740	0.435772	1	-0.609357	-0.841465	740	H9JBC1	RWDLAKFNKVSTKIGSSMKSVGEV	IGS(0.936)S(0.936)MKS(0.127)VGEV	375	Uncharacterized protein OS	UID2166	2	S	615
334	22.5341	22.7679	22.8117	22.8176	24.8436	21.9043	22.9249	23.6958	0.993661	0.0243858	95.755	48.538	95.755	-2.2332	277020000	744	0.435772	1	-0.609357	-0.841465	744	H9JBC1	AKFNKVSTKIGSSMKSVGEVMSIGI	IGS(0.757)S(0.249)MKS(0.994)VGEV	377	Uncharacterized protein OS	UID2168	2	S	615
335	NaN	20.2652	20.5308	20.2505	16.8776	NaN	19.5494	20.9337	1	0.0313731	70.488	11.812	68.536	1.8938	13552000	352	0.441784	1	1.22861	0.949561	352	H9JCG6	AKMISSKYLRWINIKTTLRGVTPKRV	T(1)T(1)LRGVT(1)PKR	1165	DUF1713 domain-containing protein OS	UID4746	3	T	644
336	NaN	20.2652	20.5308	20.2505	16.8776	NaN	19.5494	20.9337	1	0.0313731	70.488	11.812	68.536	1.8938	13552000	353	0.441784	1	1.22861	0.949561	353	H9JCG6	KMISSKYLRWINIKTTLRGVTPKRVF	T(1)T(1)LRGVT(1)PKR	1166	DUF1713 domain-containing protein OS	UID4747	3	T	644
337	NaN	20.2652	20.5308	20.2505	16.8776	NaN	19.5494	20.9337	1	0.0313731	70.488	11.812	68.536	1.8938	13552000	358	0.441784	1	1.22861	0.949561	358	H9JCG6	KYLRWINIKTTLRGVTPKRVPNIIAN	T(1)T(1)LRGVT(1)PKR	1167	DUF1713 domain-containing protein OS	UID4748	3	T	644
338	NaN	21.6087	20.0057	17.5828	21.0604	NaN	21.7432	NaN	1	0.0306731	87.568	25.022	87.568	-1.6587	14606000	177	0.44911	1	-1.66944	-1.02295	177	H9JW86	RDIENQMKVNLAAKHSLERDWSNI	VNLAAKHS(1)LER	737	Uncharacterized protein OS	UID738		S	1107
339	NaN	19.2169	17.2621	17.365	21.0932	17.5609	18.9915	NaN	0.999999	0.0387546	67.095	37.619	67.095	-0.094332	4438300	227	0.452417	1	-1.26718	-0.969911	227	H9JWH9	LKSESKCRISIDHHVYKAVNCRERHL	CRISIDHHVY(1)KAVNCR	1771	Vitellogenin domain-containing protein OS	UID1772		Y	1114
340	NaN	18.5783	NaN	17.9097	19.6181	NaN	NaN	18.4776	0.831014	0.0169848	56.468	9.4856	56.468	-3.6612	10640000	348	0.458409	1	-0.803866	-1.05627	348	H9IWG7	YLSGKFSITIAYSSVYIYVSEVFPTNVI	FS(0.073)IT(0.106)IAY(0.053)S(0.42	1523	MFS domain-containing protein OS	UID5104	3	Y	213
341	NaN	18.5783	NaN	17.9097	19.6181	NaN	NaN	18.4776	0.970866	0.0169848	56.468	9.4856	56.468	-3.6612	10640000	350	0.458409	1	-0.803866	-1.05627	350	H9IWG7	SGKFSITIAYSSVYIYVSEVFPTNVRG	FS(0.073)IT(0.106)IAY(0.053)S(0.42	1524	MFS domain-containing protein OS	UID5105	3	Y	213
342	18.2277	20.0825	19.2575	18.3992	25.4276	19.82	19.0334	18.4689	1	0.0363584	51.445	28.085	51.445	-1.0403	52238000	550	0.460357	1	-1.69578	-0.96341	550	H9JDZ3	KQEMAEKLAMMDRNAYLENRISQ	LAMMDRNAY(1)LENR	1650	GRIP domain-containing protein OS	UID1651		Y	685
343	22.3201	20.0395	22.8847	22.5334	23.9631	21.1751	24.213	NaN	0.996721	0.00153666	107.97	50.263	107.97	2.2206	90712000	807	0.466273	1	-1.17264	-0.96377	807	H9JLG0	QDQTKWAEMVIENIASSGKFSSDR	WAEMVIENIAS(0.997)S(0.003)GKFS	532	Alpha-1,4 glucan phosphorylase OS	UID2323	2	S	856
344	22.3201	20.0395	22.8847	22.5334	23.9631	21.1751	24.213	NaN	0.999886	0.00153666	107.97	50.263	107.97	2.2206	90712000	813	0.466273	1	-1.17264	-0.96377	813	H9JLG0	AEMVIENIASSGKFSSDRTITEYARE	WAEMVIENIAS(0.997)S(0.003)GKFS	533	Alpha-1,4 glucan phosphorylase OS	UID2324	2	S	856
345	23.4167	22.1209	20.3702	19.2626	NaN	19.0491	NaN	20.3581	1	0.0372671	59.182	13.73	59.182	3.7352	111070000	395	0.480029	1	1.58902	1.03325	395	H9IXT3	YALRLREQLQYVNEHSFNCFRIRIGK	LREQLQY(1)VNEHS(1)FNCFR	133	Guanylate cyclase domain-containing protein OS	UID1924	2	S	256
346	23.4167	22.1209	20.3702	19.2626	NaN	19.0491	NaN	20.3581	1	0.0372671	59.182	13.73	59.182	3.7352	111070000	390	0.480029	1	1.58902	1.03325	390	H9IXT3	TAMADYALRLREQLQYVNEHSFNC	LREQLQY(1)VNEHS(1)FNCFR	1540	Guanylate cyclase domain-containing protein OS	UID3331	2	Y	256
347	20.2561	19.5702	22.3187	20.4728	21.4653	19.9777	22.4669	22.1087	1	0.0229031	58.098	20.542	58.098	0.67659	39263000	4	0.480039	1	-0.850225	-0.94031	4	H9J335	MAESSEAAHPVPNI	AES(1)S(1)EAAHPVPNFR	236	Uncharacterized protein OS	UID2027	2	S	386
348	20.2561	19.5702	22.3187	20.4728	21.4653	19.9777	22.4669	22.1087	1	0.0229031	58.098	20.542	58.098	0.67659	39263000	5	0.480039	1	-0.850225	-0.94031	5	H9J335	MAESSEAAHPVPNFI	AES(1)S(1)EAAHPVPNFR	237	Uncharacterized protein OS	UID2028	2	S	386
349	17.8165	17.9001	NaN	16.3373	19.6267	NaN	NaN	17.4961	0.80811	0.0318682	54.393	17.865	54.393	3.5155	2039400	188	0.488619	1	-1.21011	-1.07135	188	H9JQR3	ERVSTDQTVEMLLQESSGKSTGNKI	VSTDQTVEMLLQES(0.808)S(0.191)G	621	Uncharacterized protein OS	UID622		S	961
350	NaN	21.3946	21.534	22.3414	17.5299	21.7646	21.3056	21.1879	0.999261	0.0329213	40.352	6.555	40.352	1.5748	39549000	10	0.495237	1	1.3097	1.01851	10	H9JJ36	MSTNNVQVTSKKDISNTLY	MS(0.001)T(0.001)NNVQVT(0.999)S	483	Uncharacterized protein OS	UID2274		S	797
351	NaN	21.3946	21.534	22.3414	17.5299	21.7646	21.3056	21.1879	0.999281	0.0329213	40.352	6.555	40.352	1.5748	39549000	9	0.495237	1	1.3097	1.01851	9	H9JJ36	MSTNNVQVTSKKDISNTL'	MS(0.001)T(0.001)NNVQVT(0.999)S	1251	Uncharacterized protein OS	UID3042		T	797
352	24.3832	22.2541	25.8072	NaN	18.6203	21.6778	24.0945	24.2146	0.897747	0.0208812	54.047	22.009	48.706	-2.9147	126730000	259	0.504859	1	1.99635	1.06258	259	H9J4R2	AAPYIVLKMLSGLTSTINENLNDPN'	MLS(0.027)GLT(0.036)S(0.04)T(0.89	1038	SH3 domain-containing protein OS	UID2829	2	T	429
353	24.3832	22.2541	25.8072	NaN	18.6203	21.6778	24.0945	24.2146	0.999101	0.0208812	54.047	22.009	54.047	-0.79396	126730000	269	0.504859	1	1.99635	1.06258	269	H9J4R2	SGLTSTINENLNDPNTWQDPLRAIA	MLS(0.215)GLT(0.025)S(0.025)T(0.7	1039	SH3 domain-containing protein OS	UID2830	2	T	429
354	20.0646	20.9637	19.2565	21.0634	20.9513	19.4949	21.6198	23.2446	0.876212	0.0338253	75.378	18.272	75.378	3.6586	87180000	355	0.513749	1	-0.990584	-1.00451	355	H9J432	FVTYTTVYCSRLLVYSAMTPILRHIG'	LLVY(0.817)S(0.876)AMT(0.307)PILF	248	Na H Exchanger domain-containing protein OS	UID2039	2	S	402
355	20.0646	20.9637	19.2565	21.0634	20.9513	19.4949	21.6198	23.2446	0.816864	0.0338253	75.378	18.272	75.378	3.6586	87180000	354	0.513749	1	-0.990584	-1.00451	354	H9J432	IFVTYTTVYCSRLLVYSAMTPILRHIG	LLVY(0.817)S(0.876)AMT(0.307)PILF	1581	Na H Exchanger domain-containing protein OS	UID3372	2	Y	402
356	NaN	19.6882	15.8253	16.89	22.9019	16.6715	21.3998	17.9917	0.999999	0.0185118	67.519	23.728	64.65	-0.93033	20470000	458	0.522657	1	-2.27339	-1.09948	458	H9JCA0	YVWDVEKTIISLPLQTGVMLRSFVY	T(0.123)IIS(0.877)LPLQT(1)GVMLR	1163	WD_REPEATS_REGION domain-containing protein	UID2954	2	T	640
357	18.1872	20.421	21.0084	19.2905	23.5693	21.6947	18.5892	20.5723	1	0.0252369	73.386	15.066	73.386	3.2974	31468000	312	0.52353	1	-1.37957	-1.04899	312	H9J294	KGKLNEKQAGQKIALTVTLVMATLI	IALT(1)VT(1)LVMAT(1)LIK	1003	GFO IDH MocA domain-containing protein OS	UID4584	3	T	363
358	18.1872	20.421	21.0084	19.2905	23.5693	21.6947	18.5892	20.5723	1	0.0252369	73.386	15.066	73.386	3.2974	31468000	314	0.52353	1	-1.37957	-1.04899	314	H9J294	KLNEKQAGQKIALTVTLVMATLIKTI	IALT(1)VT(1)LVMAT(1)LIK	1004	GFO IDH MocA domain-containing protein OS	UID4585	3	T	363
359	18.1872	20.421	21.0084	19.2905	23.5693	21.6947	18.5892	20.5723	1	0.0252369	73.386	15.066	73.386	3.2974	31468000	319	0.52353	1	-1.37957	-1.04899	319	H9J294	QAGQKIALTVTLVMATLIKTHLARR'	IALT(1)VT(1)LVMAT(1)LIK	1005	GFO IDH MocA domain-containing protein OS	UID4586	3	T	363
360	20.5176	NaN	19.5984	17.4691	NaN	16.2982	NaN	18.4985	0.993652	0.0313685	59.372	25.684	59.372	3.041	9015300	185	0.527926	1	1.79668	1.17808	185	H9IZC2	MQDLLKGSHSGSIYNSTEIRNLGKV	KMQDLLKGSHSGSIY(0.008)NS(0.994	167	Uncharacterized protein OS	UID1958	2	S	288
361	20.5176	NaN	19.5984	17.4691	NaN	16.2982	NaN	18.4985	0.997993	0.0313685	59.372	25.684	59.372	3.041	9015300	186	0.527926	1	1.79668	1.17808	186	H9IZC2	QDLLKGSHSGSIYNSTEIRNLGKVFY	KMQDLLKGSHSGSIY(0.008)NS(0.994	955	Uncharacterized protein OS	UID2746	2	T	288
362	23.5279	19.7879	19.5598	NaN	NaN	20.2602	18.5359	19.0246	0.804442	0.0279104	46.873	19.07	46.873	2.7868	16981000	467	0.53668	1	1.68497	1.13471	467	H9IW46	MKGGYLKDYDEISLSSQENENFNLI	DY(0.965)DEIS(0.035)LS(0.195)S(0.8	101	Uncharacterized protein OS	UID1892	2	S	205
363	23.5279	19.7879	19.5598	NaN	NaN	20.2602	18.5359	19.0246	0.964981	0.0279104	46.873	19.07	46.873	2.7868	16981000	460	0.53668	1	1.68497	1.13471	460	H9IW46	NKYEQWEMKGGYLKDYDEISLSSQ	DY(0.965)DEIS(0.035)LS(0.195)S(0.8	1522	Uncharacterized protein OS	UID3313	2	Y	205
364	NaN	18.8537	18.9721	NaN	18.4758	NaN	22.3502	22.6014	0.803758	0.0339722	58.723	27.046	58.723	-1.1704	13964000	533	0.542729	1	-2.2296	-1.22208	533	H9IT00	ENLERLHCEQFRLRCYMASLQSGEL	LRCY(0.804)MAS(0.196)LQSGELPNPI	1494	Uncharacterized protein OS	UID1495		Y	130
365	NaN	NaN	22.5322	20.8442	NaN	21.7572	23.6387	24.5433	0.997149	0.0346766	65.809	29.13	65.809	2.2723	65209000	2143	0.554593	1	-1.62483	-1.21979	2143	H9JJW5	VRNTDRGNNPKGYLPSHYERVQMI	NT(0.003)DRGNNPKGY(1)LPS(0.997)	499	MPN domain-containing protein OS	UID4080	3	S	813
366	NaN	NaN	22.5322	20.8442	NaN	21.7572	23.6387	24.5433	0.999882	0.0346766	65.809	29.13	65.809	2.2723	65209000	2140	0.554593	1	-1.62483	-1.21979	2140	H9JJW5	EWGVRNTDRGNNPKGYLPSHYER\	NT(0.003)DRGNNPKGY(1)LPS(0.997)	1680	MPN domain-containing protein OS	UID5261	3	Y	813
367	NaN	NaN	22.5322	20.8442	NaN	21.7572	23.6387	24.5433	0.999876	0.0346766	65.809	29.13	65.809	2.2723	65209000	2145	0.554593	1	-1.62483	-1.21979	2145	H9JJW5	NTDRGNNPKGYLPSHYERVQMLLS	NT(0.003)DRGNNPKGY(1)LPS(0.997)	1681	MPN domain-containing protein OS	UID5262	3	Y	813
368	19.2209	NaN	NaN	17.3738	NaN	16.3295	NaN	17.1862	0.998316	0.0305147	55.414	7.793	50.158	0.79248	6284300	203	0.569242	1	1.5395	1.37696	203	Q2F646	KATFCLPIIGVKKNPSSQMYTSLGVI	KNPS(0.998)S(0.998)QMY(0.996)T(0	821	Hairy cell leukemia protein 1 OS	UID4402		S	1215
369	19.2209	NaN	NaN	17.3738	NaN	16.3295	NaN	17.1862	0.998486	0.0305147	55.414	7.793	50.158	0.79248	6284300	204	0.569242	1	1.5395	1.37696	204	Q2F646	ATFCLPIIGVKKNPSSQMYTSLGVIT	KNPS(0.998)S(0.998)QMY(0.996)T(0	822	Hairy cell leukemia protein 1 OS	UID4403		S	1215
370	19.2209	NaN	NaN	17.3738	NaN	16.3295	NaN	17.1862	0.995916	0.0305147	55.414	7.793	50.158	0.79248	6284300	207	0.569242	1	1.5395	1.37696	207	Q2F646	CLPIIGVKKNPSSQMYTSLGVITKGT	KNPS(0.998)S(0.998)QMY(0.996)T(0	1782	Hairy cell leukemia protein 1 OS	UID5363		Y	1215
371	18.7605	20.0113	19.0404	18.741	22.5125	NaN	NaN	18.9192	0.776778	0.0324942	58.98	15.576	58.98	-3.7469	14241000	230	0.593562	1	-1.57756	-1.22474	230	H9JKM6	SEIYPQSHNQISKMVSQMTAVQYA	MVS(0.777)QMT(0.223)AVQYAPVVK	519	Chitin-binding type-2 domain-containing protein O	UID520		S	832
372	20.7387	19.7309	23.3627	20.0812	24.0951	20.1589	22.013	23.9818	0.915853	0.0281842	43.383	8.2064	43.383	1.5616	70890000	69	0.602453	1	-1.58384	-1.17918	69	H9J190	VLANLTTKLYKLKEKYGIYNEEIGIPA	EKY(0.916)GIY(0.916)NEEIGIPAT(0.1	1566	Uncharacterized protein OS	UID5147	3	Y	334
373	20.7387	19.7309	23.3627	20.0812	24.0951	20.1589	22.013	23.9818	0.915853	0.0281842	43.383	8.2064	43.383	1.5616	70890000	72	0.602453	1	-1.58384	-1.17918	72	H9J190	NLTTKLYKLKEKYGIYNEEIGIPATDA	EKY(0.916)GIY(0.916)NEEIGIPAT(0.1	1567	Uncharacterized protein OS	UID5148	3	Y	334
374	18.9402	NaN	19.5994	17.9376	18.9769	19.6316	19.183	20.266	0.999821	0.0350151	40.186	10.053	40.186	2.7221	6843600	312	0.605636	1	-0.688657	-1.09883	312	H9J314	DATTVLSRAIAELGVYPAVDPLDSTS	AIAELGVY(1)PAVDPLDS(0.491)T(0.4	1578	ATP synthase subunit beta OS	UID3369	2	Y	384
375	NaN	20.9582	NaN	22.8037	NaN	20.4862	20.2464	NaN	0.945396	0.00854077	86.777	18.898	86.777	-0.34597	53672000	189	0.610648	1	1.51463	1.46983	189	H9JTW5	ASLDTSLRTITDEQPTLYYALVDDRR	T(0.055)IT(0.968)DEQPT(0.945)LY(0.	1394	Uncharacterized protein OS	UID3185	2	T	1051
376	NaN	15.6362	19.2547	19.3366	25.5566	19.258	19.1207	19.6316	0.982078	0.0250211	43.233	12.059	43.233	-0.025355	61785000	276	0.621308	1	-2.81593	-1.27538	276	H9JHY8	QQARDAQCYNNKPLVTNTMGLSPI	PLVT(0.982)NT(0.98)MGLS(0.036)PI	1240	Lipase domain-containing protein OS	UID3031	2	T	773
377	NaN	15.6362	19.2547	19.3366	25.5566	19.258	19.1207	19.6316	0.979703	0.0250211	43.233	12.059	43.233	-0.025355	61785000	278	0.621308	1	-2.81593	-1.27538	278	H9JHY8	ARDAQCYNNKPLVTNTMGLSPDKS	PLVT(0.982)NT(0.98)MGLS(0.036)PI	1241	Lipase domain-containing protein OS	UID3032	2	T	773
378	17.9828	18.5942	18.4592	19.6372	18.3624	NaN	17.3127	18.3163	0.922962	0.0388925	51.127	17.234	51.127	-2.2147	4402500	500	0.621693	1	0.67122	1.11463	500	E9JEG5	KWGSRPSKLTTQKSITISTDVLKDIIP	PS(0.369)KLT(0.308)T(0.309)QKS(0.(	855	Acinus OS	UID4436		T	97
379	17.9828	18.5942	18.4592	19.6372	18.3624	NaN	17.3127	18.3163	0.834334	0.0388925	51.127	17.234	51.127	-2.2147	4402500	503	0.621693	1	0.67122	1.11463	503	E9JEG5	SRPSKLTTQKSITISTDVLKDIIPNVKI	PS(0.369)KLT(0.308)T(0.309)QKS(0.(	856	Acinus OS	UID4437		T	97
380	16.7027	17.3535	16.8044	NaN	NaN	16.6318	16.4887	NaN	1	0.0375537	46.001	20.155	46.001	-3.3564	582070	275	0.626521	1	0.393248	1.07344	275	H9IZF6	IKPENKMTTIGAKYISKKDLVLMKLF	MT(1)T(1)IGAKY(1)IS(1)KK	169	Uncharacterized protein OS	UID3750	4	S	295
381	16.7027	17.3535	16.8044	NaN	NaN	16.6318	16.4887	NaN	1	0.0375537	46.001	20.155	46.001	-3.3564	582070	267	0.626521	1	0.393248	1.07344	267	H9IZF6	ETKLALYTIKPENKMTTIGAKYISKKI	MT(1)T(1)IGAKY(1)IS(1)KK	959	Uncharacterized protein OS	UID4540	4	T	295
382	16.7027	17.3535	16.8044	NaN	NaN	16.6318	16.4887	NaN	1	0.0375537	46.001	20.155	46.001	-3.3564	582070	268	0.626521	1	0.393248	1.07344	268	H9IZF6	TKLALYTIKPENKMTTIGAKYISKKDI	MT(1)T(1)IGAKY(1)IS(1)KK	960	Uncharacterized protein OS	UID4541	4	T	295
383	16.7027	17.3535	16.8044	NaN	NaN	16.6318	16.4887	NaN	1	0.0375537	46.001	20.155	46.001	-3.3564	582070	273	0.626521	1	0.393248	1.07344	273	H9IZF6	YTIKPENKMTTIGAKYISKKDLVLMI	MT(1)T(1)IGAKY(1)IS(1)KK	1555	Uncharacterized protein OS	UID5136	4	Y	295
384	20.1576	22.6854	21.9655	21.9822	24.9113	22.2797	22.4941	21.7708	0.933632	0.0271647	111.07	7.0025	111.07	-1.2559	89995000	10	0.629142	1	-1.16631	-1.18598	10	H9J4Z2	MLSLGSGVGTEPKSRWGR	MLSLGS(0.028)GVGT(0.934)EPKS(0.	1049	Fork-head domain-containing protein OS	UID1050	1	T	436
385	19.4157	17.6658	18.2385	19.4266	NaN	17.7852	17.3478	NaN	1	0.0388299	42.647	17.036	42.647	-4.201	9571500	618	0.765719	0.91882	1.12014	1.44855	618	H9JCA0	TLQGAVSPVSGRRMRSVLVSPNYS	MRS(1)VLVS(1)PNY(1)S(1)LRDQLR	386	WD_REPEATS_REGION domain-containing protein	UID3967		S	640
386	19.4157	17.6658	18.2385	19.4266	NaN	17.7852	17.3478	NaN	1	0.0388299	42.647	17.036	42.647	-4.201	9571500	622	0.765719	0.904	1.12014	1.44855	622	H9JCA0	AVSPVSGRRMRSVLVSPNYSLRDQ	MRS(1)VLVS(1)PNY(1)S(1)LRDQLR	387	WD REPEATS REGION domain-containing protein	UID3968		S	640
387	19.4157	17.6658	18.2385	19.4266	NaN	17.7852	17.3478	NaN	1	0.0388299	42.647	17.036	42.647	-4.201	9571500	626	0.765719	0.889651	1.12014	1.44855	626	H9JCA0	VSGRRMRSVLVSPNYSLRDQLRTFI	MRS(1)VLVS(1)PNY(1)S(1)LRDQLR	388	WD REPEATS REGION domain-containing protein	UID3969		S	640
388	19.4157	17.6658	18.2385	19.4266	NaN	17.7852	17.3478	NaN	1	0.0388299	42.647	17.036	42.647	-4.201	9571500	625	0.765719	0.934133	1.12014	1.44855	625	H9JCA0	PVSGRRMRSVLVSPNYSLRDQLRTF	MRS(1)VLVS(1)PNY(1)S(1)LRDQLR	1635	WD REPEATS REGION domain-containing protein	UID5216		Y	640
389	21.0123	20.9682	20.3438	21.6975	22.2481	20.3062	22.6829	22.7901	0.998216	2.74E-98	293.53	251.57	268.9	1.5061	537900000	1073	0.770636	0.900375	-1.0014	-1.35039	1073	P07856	GASSSSASTAKSDAASSEDGFWWV	SDAAS(0.998)S(0.002)EDGFWWWNI	793	Sericin 1 OS	UID794	1	S	1174
390	19.2438	19.5185	19.2015	19.29	NaN	19.2748	20.1292	19.6195	1	0.00411742	116.77	64.925	116.77	3.2794	11219000	174	0.775449	1	-0.361023	-1.11479	174	H9JF91	CDLPVKKMNIIIDIETDPDPRLRLNT	KMNIIIDIET(1)DPDPR	1211	Uncharacterized protein OS	UID1212	1	T	714
391	23.7492	23.5792	23.2564	23.6082	23.9463	25.1764	25.0246	23.1377	0.997835	0.0339655	43.799	17.923	43.799	-4.4283	359620000	637	0.77794	0.968985	-0.773009	-1.30713	637	H9J4Z5	LKASVEYKDSTKAQVYIQAIGNLGH	AS(0.002)VEY(0.061)KDS(0.47)T(0.4'	1591	Uncharacterized protein OS	UID3382	2	Y	437
392	22.5137	22.2814	22.2528	21.2103	NaN	NaN	23.9916	22.3849	0.75105	0.0260754	44.582	14.448	44.582	2.1995	70169000	69	0.788979	0.909724	-1.1237	-1.48367	69	H9J2T7	PPEPPRVYAYDAVFDSNTSQCILRY	VY(0.784)AY(0.697)DAVFDS(0.751)N	231	Kinesin motor domain-containing protein OS	UID3812	4	S	378
393	22.5137	22.2814	22.2528	21.2103	NaN	NaN	23.9916	22.3849	0.784077	0.0260754	44.582	14.448	44.582	2.1995	70169000	61	0.788979	0.894305	-1.1237	-1.48367	61	H9J2T7	AVTRNNVTPPEPPRVYAYDAVFDSI	VY(0.784)AY(0.697)DAVFDS(0.751)N	1575	Kinesin motor domain-containing protein OS	UID5156	4	Y	378

394	NaN	17.2695	NaN	16.681	20.8849	18.4465	NaN	18.2299	0.996669	0.0301065	54.223	14.637	54.223	-2.7203	3295600	81	0.844945	0.653571	-2.21184	-1.81215	81	H9JAI3	DSYVGSTLVLKGLRRSDMGTYLCIA	GLRRS(0.997)DMGT(0.003)YLCIAAN	361	Uncharacterized protein OS	UID362	1	S	596
395	NaN	19.6419	18.3842	21.2157	NaN	20.3227	22.3573	23.8794	1	0.0146705	103.26	26.063	103.26	-0.18975	29870000	66	0.86178	0.625891	-2.43921	-1.72237	66	H9JR36	ERFPSQNWKNNDLVHYLDALRRFS	NNDLVHY(1)LDALRR	1727	Uncharacterized protein OS	UID1728	1	Y	970
396	NaN	21.6369	20.8118	18.3489	NaN	22.3135	21.5598	24.5807	1	1.51E-64	221.51	170.02	221.51	0.65985	119140000	95	0.886515	0.596453	-2.55212	-1.77052	95	P05790	MIKTFVITTDSDGNESIVEEDVLMK	TFVITTDSDGNES(1)IVEEDVLMK	785	Fibroin heavy chain OS	UID786	1;2	S	1172
397	18.2319	18.0211	17.2923	17.7363	18.9956	17.4991	19.5449	18.646	1	0.0177485	86.189	17.563	86.189	2.6888	11946000	9	0.902606	0.785754	-0.850985	-1.47302	9	H9JWD7	MISDLNIISKIFPQPRVTLN	ISDLNIIS(1)K	739	Uncharacterized protein OS	UID740		S	1111
398	21.5423	NaN	20.1469	17.8671	23.8412	NaN	22.0753	21.2704	0.965582	0.0242974	75.017	18.977	75.017	0.42583	62473000	31	0.90393	0.644	-2.5435	-1.80022	31	H9JL75	AHDAVLWRARVGSAYSNSGGTMY	ARVGS(0.003)AY(0.005)S(0.966)NS(	529	Peptidase S1 domain-containing protein OS	UID2320		S	847
399	21.5423	NaN	20.1469	17.8671	23.8412	NaN	22.0753	21.2704	0.787095	0.0242974	75.017	18.977	75.017	0.42583	62473000	36	0.90393	0.658311	-2.5435	-1.80022	36	H9JL75	LWRARVGSAYSNSGGTMYIISRITP	ARVGS(0.003)AY(0.005)S(0.966)NS(	1284	Peptidase S1 domain-containing protein OS	UID3075		T	847
400	18.7547	NaN	19.5074	17.3852	NaN	16.0529	17.1822	NaN	0.958227	0.0281547	56.551	14.991	56.551	-1.1722	4629700	860	0.911167	0.62672	1.93151	1.92064	860	H9J7V2	NDNASSVVRRVYVYMSVTNRSISSI	RVY(0.001)VY(0.043)MS(0.958)VT(0	316	ASH domain-containing protein OS	UID2107	2	S	515
401	18.7547	NaN	19.5074	17.3852	NaN	16.0529	17.1822	NaN	0.997623	0.0281547	56.551	14.991	56.551	-1.1722	4629700	862	0.911167	0.614431	1.93151	1.92064	862	H9J7V2	NASSVVRRVYVYMSVTNRSISSIEVI	RVY(0.001)VY(0.043)MS(0.958)VT(0	1096	ASH domain-containing protein OS	UID2887	2	T	515
402	21.9502	20.9582	NaN	22.8037	NaN	20.4862	20.2464	NaN	0.982057	0.00615619	86.777	18.898	79.471	-2.048	86475000	184	0.944804	0.655404	1.53772	1.93625	184	H9JTW5	MLLKQASLDTSLRTITDEQPTLYYAL	T(0.973)IT(0.982)DEQPT(0.041)LY(0.	1393	Uncharacterized protein OS	UID3184	2	T	1051
403	16.5215	17.1177	19.1408	18.9831	19.2095	18.3431	20.1819	19.7572	1	0.00186202	144.12	57.986	144.12	-2.984	7139600	24	0.950453	0.602615	-1.43217	-1.6469	24	H9JYJ2	TIVKKRMRHVLLVQKSKDSQKCPD	MRHVLLVQKS(1)K	781	Uncharacterized protein OS	UID782	1	S	1166
404	17.4659	20.9026	22.7756	22.2115	23.5558	NaN	NaN	26.012	1	0.0190744	99.834	55.728	99.834	-1.1824	318750000	201	0.952676	0.588	-3.94499	-1.93563	201	H9IZH2	VRTKLSPNSRRAELNYDVKVFAEHG	RAELNY(1)DVKVFAEHGR	1556	Uncharacterized protein OS	UID1557	1	Y	297
405	20.2096	20.5249	21.2413	21.6703	21.2023	21.098	23.4251	23.544	0.999991	0.00876164	62.028	25.597	62.028	-1.2867	73426000	694	0.955395	0.63951	-1.40585	-1.64976	694	H9JUX0	HCNLINQSFALSEEASADAIKKAQQ	NHCNLINQSFALSEEAS(1)ADAIKK	714	THAP-type domain-containing protein OS	UID715	1	S	1079
406	NaN	17.4474	16.8081	18.4536	18.6123	17.7785	19.1161	19.9008	0.9994	0.0267606	54.501	13.981	40.237	1.8743	12940000	376	0.956672	0.587778	-1.28221	-1.6828	376	H9J2R9	MQKDLEHYKSEIAELSRTKEKLSARV	MQKDLEHY(0.469)KS(0.532)EIAELS(	230	CAP-Gly domain-containing protein OS	UID2021	2	S	376
407	22.2516	19.1662	20.4978	NaN	22.265	NaN	22.2348	23.8194	0.987775	0.0331524	68.893	36.37	68.893	2.7111	55929000	85	0.965538	0.613636	-2.13453	-1.88	85	H9J5F2	SDDRICFNHGKELYVYVYRGVKKAA	ELY(0.011)VY(0.988)VY(0.001)RGVK	1594	WD_REPEATS_REGION domain-containing protein	UID1595		Y	457
408	NaN	17.3976	16.8081	18.4536	18.6123	17.7785	19.1161	19.9008	0.998675	0.033023	49.559	23.085	43.024	0.047392	8705100	370	0.968626	0.647333	-1.29883	-1.70189	370	H9J2R9	KHEMQKMQKDLEHYKSEIAELSRTI	MQKDLEHY(0.999)KS(0.999)EIAELS(	229	CAP-Gly domain-containing protein OS	UID2020	2	S	376
409	18.2711	22.9549	21.3505	23.9751	23.8398	23.2591	25.0509	25.745	0.989644	0.0167627	51.044	9.0021	42.863	-3.5767	286670000	709	1.07852	0.588485	-2.8358	-1.93238	709	H9IZ62	KLDNYVELWNDGRWHSLMLTLAP	WHS(0.99)LMLT(0.99)LAPDS(0.018)	164	Uncharacterized protein OS	UID3745	3	S	286
410	18.2711	22.9549	21.3505	23.9751	23.8398	23.2591	25.0509	25.745	0.767672	0.0167627	51.044	9.0021	42.863	-3.5767	286670000	731	1.07852	0.606875	-2.8358	-1.93238	731	H9IZ62	PDSLVLSMDSRAVHTSKRLRFLTGS	WHS(0.99)LMLT(0.99)LAPDS(0.018)	165	Uncharacterized protein OS	UID3746	3	S	286
411	18.2711	22.9549	21.3505	23.9751	23.8398	23.2591	25.0509	25.745	0.989563	0.0167627	51.044	9.0021	42.863	-3.5767	286670000	713	1.07852	0.626452	-2.8358	-1.93238	713	H9IZ62	YVELWNDGRWHSLMLTLAPDSLVI	WHS(0.99)LMLT(0.99)LAPDS(0.018)	953	Uncharacterized protein OS	UID4534	3	T	286
412	22.0941	22.1942	21.2623	20.8761	NaN	NaN	22.4172	23.6769	0.780519	0.0317572	48.771	15.917	48.771	-1.9176	58123000	710	1.0982	0.61773	-1.44041	-2.00972	710	H9JWU4	SVQQNTCELDPAAVQTSERKLAELE	S(0.001)T(0.001)MRGRMGS(0.489)S	1424	Structural maintenance of chromosomes protein C	UID3215	2	T	1120
413	17.9647	18.2707	16.8951	18.1603	22.4874	18.423	19.5491	18.9534	1	0.0285948	71.692	7.0384	71.692	0.99564	15791000	67	1.1021	0.585294	-2.03049	-1.9138	67	H9J555	VEHDLRVMGIDWAPNTNRIVTCSV	VMGIDWAPNT(1)NR	1054	Actin-related protein 2/3 complex subunit OS	UID1055		T	445
414	16.892	18.3243	17.6015	17.3541	19.0783	NaN	18.396	NaN	0.872299	0.00590808	60.528	34.593	60.528	-0.54745	4551900	93	1.13486	0.56	-1.19417	-2.00746	93	H9J8M7	KLELEDGDVVMLKKNSRHYLPTSEC	NS(0.872)RHY(0.15)LPT(0.978)S(0.9	337	Uncharacterized protein OS	UID3918	4	S	550
415	16.892	18.3243	17.6015	17.3541	19.0783	NaN	18.396	NaN	0.998874	0.00590808	60.528	34.593	60.528	-0.54745	4551900	100	1.13486	0.574	-1.19417	-2.00746	100	H9J8M7	DVVMLKKNSRHYLPTSECQTLIRQG	NS(0.872)RHY(0.15)LPT(0.978)S(0.9	338	Uncharacterized protein OS	UID3919	4	S	550
416	16.892	18.3243	17.6015	17.3541	19.0783	NaN	18.396	NaN	0.978369	0.00590808	60.528	34.593	60.528	-0.54745	4551900	99	1.13486	0.588718	-1.19417	-2.00746	99	H9J8M7	GDVVMLKKNSRHYLPTSECQTLIRC	NS(0.872)RHY(0.15)LPT(0.978)S(0.9	1108	Uncharacterized protein OS	UID4689	4	T	550
417	16.892	18.3243	17.6015	17.3541	19.0783	NaN	18.396	NaN	0.999996	0.00590808	60.528	34.593	60.528	-0.54745	4551900	104	1.13486	0.604211	-1.19417	-2.00746	104	H9J8M7	LKKNSRHYLPTSECQTLIRQGILKQI	NS(0.872)RHY(0.15)LPT(0.978)S(0.9	1109	Uncharacterized protein OS	UID4690	4	T	550
418	17.8329	NaN	15.8146	16.8959	18.3061	17.5461	18.1741	18.159	0.934579	0.0247081	65.038	28.621	65.038	-3.351	3881000	96	1.14061	0.6368	-1.1985	-1.90927	96	H9J6N8	NLSNTKILILCQDNRTCSQLNHFLTN	T(0.935)CS(0.066)QLNHFLT(1)MGAN	1071	Uncharacterized protein OS	UID2862	2	T	480
419	17.8329	NaN	15.8146	16.8959	18.3061	17.5461	18.1741	18.159	0.999741	0.0247081	65.038	28.621	65.038	-3.351	3881000	105	1.14061	0.619111	-1.1985	-1.90927	105	H9J6N8	LCQDNRTCSQLNHFLTMGANRYLL	T(0.935)CS(0.066)QLNHFLT(1)MGAN	1072	Uncharacterized protein OS	UID2863	2	T	480
420	NaN	17.411	NaN	17.2021	23.1646	20.6153	NaN	NaN	0.977747	0.0240244	43.752	16.697	43.752	3.0822	13992000	10	1.15611	0.56352	-4.58344	-3.32391	10	H9IZV7	MSAVVLFGASGFLLYKLFIY	MS(0.02)AVVLFGAS(0.978)GFLLY(0.	178	Uncharacterized protein OS	UID1969	2	S	306
421	NaN	17.411	NaN	17.2021	23.1646	20.6153	NaN	NaN	0.998795	0.0240244	43.752	16.697	43.752	3.0822	13992000	20	1.15611	0.542769	-4.58344	-3.32391	20	H9IZV7	VLFGASGFLLYKLFIYRHFIGVNVEYI	MS(0.02)AVVLFGAS(0.978)GFLLY(0.	1560	Uncharacterized protein OS	UID3351	2	Y	306
422	18.3696	20.5464	20.1074	21.9384	21.8239	21.0286	23.1534	24.0333	0.999263	0.0264888	69.024	22.238	69.024	-3.3111	70327000	13	1.20168	0.555556	-2.26933	-2.07135	13	H9JLZ8	MALSKRKPSKARSSSSYVFTDG	ARS(0.999)S(0.194)S(0.564)S(0.196)	541	Uncharacterized protein OS	UID4122	3	S	867
423	18.3696	20.5464	20.1074	21.9384	21.8239	21.0286	23.1534	24.0333	0.971988	0.0264888	69.024	22.238	69.024	-3.3111	70327000	20	1.20168	0.535714	-2.26933	-2.07135	20	H9JLZ8	KRKPSKARSSSSYVFTDGTLKLLRIR	ARS(0.999)S(0.194)S(0.564)S(0.196)	1296	Uncharacterized protein OS	UID4877	3	T	867
424	17.481	19.5812	18.1652	17.7599	NaN	17.0523	16.9373	16.7167	1	0.0364705	44.377	8.0595	44.377	-0.88214	2995300	2	1.21553	0.599867	1.34474	2.04359	2	H9JSX6	MYFPIGWPKVLK	Y(1)FPIGWPKVLK	1747	Uncharacterized protein OS	UID1748		Y	1017
425	19.6405	19.1652	18.7762	19.3296	20.3745	19.1925	20.642	20.0826	0.999979	0.0203618	63.894	28.995	60.937	-0.032398	47186000	414	1.23176	0.567524	-0.845048	-1.82637	414	H9JDJ7	GRMGNLGVATSFFNDTNRGLARD	MGNLGVATSFFNDT(1)NR	1182	Uncharacterized protein OS	UID1183	1	T	671
426	NaN	17.1798	17.1051	17.6847	NaN	18.2726	17.9373	19.2234	0.826678	0.0198063	66.674	22.062	66.674	-2.5429	3242000	3	1.27133	0.597103	-1.15455	-2.1948	3	H9IVH2	MASSRLQVTARQ	MAS(0.827)S(0.173)RLQVTARQLSK	90	Uncharacterized protein OS	UID91		S	189
427	NaN	17.8984	NaN	17.1356	NaN	NaN	19.2822	19.8547	0.999845	0.034421	44.511	10.129	44.511	2.0726	2495400	4	1.30091	0.467333	-2.05147	-3.55626	4	H9J039	MFLSPGEVERLLRE	FLS(1)PGEVERLLREYR	188	Uncharacterized protein OS	UID189	1	S	313
428	22.8675	21.2197	21.0001	21.6617	24.7882	22.192	25.5092	22.9607	1	0.0362329	40.589	14.609	40.589	-1.7138	264330000	228	1.3186	0.470545	-2.17526	-2.22356	228	A4ZWB1	MADFGFRKIMVFLGYSSFPKLMKK	IMVFLGY(1)S(1)S(1)FPKLMK	0	Cytochrome P450 OS	UID3581	3	S	1
429	22.8675	21.2197	21.0001	21.6617	24.7882	22.192	25.5092	22.9607	1	0.0362329	40.589	14.609	40.589	-1.7138	264330000	229	1.3186	0.450087	-2.17526	-2.22356	229	A4ZWB1	ADFGFRKIMVFLGYSSFPKLMKKF	IMVFLGY(1)S(1)S(1)FPKLMK	1	Cytochrome P450 OS	UID3582	3	S	1
430	22.8675	21.2197	21.0001	21.6617	24.7882	22.192	25.5092	22.9607	1	0.0362329	40.589	14.609	40.589	-1.7138	264330000	227	1.3186	0.492952	-2.17526	-2.22356	227	A4ZWB1	DMADFGFRKIMVFLGYSSFPKLMK	IMVFLGY(1)S(1)S(1)FPKLMK	1468	Cytochrome P450 OS	UID5049	3	Y	1
431	17.851	18.4386	17.6159	NaN	21.958	19.1741	18.9751	21.1228	0.998906	0.00164592	70.412	38.137	70.412	3.1844	14121000	9	1.32938	0.55225	-2.33902	-2.35976	9	H9J010	MGTLNLRLSYHDMRMFA	LS(0.999)Y(0.999)HDMRMFAS(0.002	185	Uncharacterized protein OS	UID3766	3	S	310
432	17.851	18.4386	17.6159	NaN	21.958	19.1741	18.9751	21.1228	0.999972	0.00164592	70.412	38.137	70.412	3.1844	14121000	22	1.32938	0.519765	-2.33902	-2.35976	22	H9J010	RLSYHDMRMFASMLQSLPVQVRA	LS(0.999)Y(0.999)HDMRMFAS(0.002	186	Uncharacterized protein OS	UID3767	3	S	310
433	17.851	18.4386	17.6159	NaN	21.958	19.1741	18.9751	21.1228	0.999094	0.00164592	70.412	38.137	70.412	3.1844	14121000	10	1.32938	0.589067	-2.33902	-2.35976	10	H9J010	MGTLNLRLSYHDMRMFAS	LS(0.999)Y(0.999)HDMRMFAS(0.002	1561	Uncharacterized protein OS	UID5142	3	Y	310
434	NaN	19.3988	18.5719	19.2232	20.6025	20.1172	21.8192	23.5365	1	0.0322478	55.707	11.909	55.707	-0.01605	32828000	499	1.34331	0.660615	-2.45419	-2.39271	499	E9JEJ4	TLRGVYEEGGRILAVTELCRGGELLE	ILAVT(1)ELCRGGELLEHIT(1)QR	859	Ribosomal protein S6 kinase OS	UID2650		T	101
435	NaN	19.3988	18.5719	19.2232	20.6025	20.1172	21.8192	23.5365	1	0.0322478	55.707	11.909	55.707	-0.01605	32828000	512	1.34331	0.613429	-2.45419	-2.39271	512	E9JEJ4	AVTELCRGGELLEHITQRRYLPEHEA	ILAVT(1)ELCRGGELLEHIT(1)QR	860	Ribosomal protein S6 kinase OS	UID2651		T	101
436	23.8447	23.6956	25.0348	24.3144	24.8516	25.7719	25.1146	26.862	0.999714	0.0351816	68.168	8.0652	68.168	3.4035	447030000	71	1.41691	0.4692	-1.42765	-2.23146	71	H9JH19	EGSPFLNRRGILGGVSTHTTPVLGR	GILGGVS(1)T(1)HT(0.818)T(0.183)P	455	Uncharacterized protein OS	UID4036	3	S	748
437	23.8447	23.6956	25.0348	24.3144	24.8516	25.7719	25.1146	26.862	0.999756	0.0351816	68.168	8.0652	68.168	3.4035	447030000	72	1.41691	0.493895	-1.42765	-2.23146	72	H9JH19	GSPFLNRRGILGGVSTHTTPVLGRR	GILGGVS(1)T(1)HT(0.818)T(0.183)P	1232	Uncharacterized protein OS	UID4813	3	T	748
438	23.8447	23.6956	25.0348	24.3144	24.8516	25.7719	25.1146	26.862	0.817531	0.0351816	68.168	8.0652	68.168	3.4035	447030000	74	1.41691	0.521333	-1.42765	-2.23146	74	H9JH19	PFLNRRGILGGVSTHTTPVLGRRSD	GILGGVS(1)T(1)HT(0.818)T(0.183)P	1233	Uncharacterized protein OS	UID4814	3	T	748
439	17.7083	18.8292	17.9673	17.4682	22.4689	NaN	NaN	19.5698	0.874786	0.000434192	115.57	47.87	79.337	-0.772	21259000	272	1.41885	0.840444	-3.02614	-2.76871	272	H9IS30	LVYLEAVIKETLRMYSVVPAIARKTD	MY(0.963)S(0.875)VVPAIARKT(0.16	43	Uncharacterized protein OS	UID1834	2	S	112
440	17.7083	18.8292	17.9673	17.4682	22.4689	NaN	NaN	19.5698	0.986282	0.000434192	115.57	47.87	51.147	-0.81242	20152000	281	1.41885	0.7564	-3.02614	-2.76871	281	H9IS30	ETLRMYSVVPAIARKTDIDLKLKNYT	MY(0.476)S(0.537)VVPAIARKT(0.98	867	Uncharacterized protein OS	UID2658	2	T	112
441	NaN	18.1737	19.6045	18.7719	20.8463	NaN	NaN	21.0418	0.924771	0.0382526	43.798	6.7066	43.798	-3.4592	33290000	190	1.51642	0.981714	-2.09398	-3.26904	190	H9J1S6	PDKDKCERAFEVHKCSYEKDPDVSI	CS(0.925)Y(0.158)EKDPDVS(0.919)I	217	Uncharacterized protein OS	UID3798		S	352
442	NaN	18.1737	19.6045	18.7719	20.8463	NaN	NaN	21.0418	0.919066	0.0382526	43.798	6.7066	43.798	-3.4592	33290000	198	1.51642	1	-2.09398	-3.26904	198	H9J1S6	AFEVHKCSYEKDPDVSIVTILNVREK	CS(0.925)Y(0.158)EKDPDVS(0.919)I	218	Uncharacterized protein OS	UID3799		S	352
443	NaN	18.1737	19.6045	18.7719	20.8463	NaN	NaN	21.0418	0.99862	0.0382526	43.798	6.7066	43.798	-3.4592	33290000	201	1.51642	0.859	-2.09398	-3.26904	201	H9J1S6	VHKCSYEKDPDVSIVTILNVREK	CS(0.925)Y(0.158)EKDPDVS(0.919)I	995	Uncharacterized protein OS	UID4576		T	352
444	18.8363	NaN	18.8579	18.444	16.9085	NaN	NaN	17.7112	1	0.0386285	47.189	8.1843	47.189	4.3818	2969000	362	1.56859	0.630333	1.40291	3.14645	362	H9J482	FMQATSELRRTDRRATDPQHLLYIA	RAT(1)DPQHLLY(1)IAAK	1027	Helitron like N domain-containing protein OS	UID2818	2	T	407
445	18.8363	NaN	18.8579	18.444	16.9085	NaN	NaN	17.7112	1	0.0386285	47.189	8.1843	47.189	4.3818	2969000	369	1.56859	0.687636	1.40291	3.14645	369	H9J482	LRRTDRRATDPQHLLYIAAKIMKLR	RAT(1)DPQHLLY(1)IAAK	1583	Helitron like N domain-containing protein OS	UID3374	2	Y	407
446	20.6483	21.3768	21.315	19.9496	22.6843	21.9607	23.1434	22.6204	0.99996	0.0267072	55.112	13.145	55.112	1.0014	188400000	52	2.29417	0.332	-1.7798	-3.46481	52	H9JML7	DPRFLSFTVDPKYLFSTSEKYKRKEC	Y(1)LFS(1)T(1)S(1)EKYKR	554	Uncharacterized protein OS	UID4135	4	S	884
447	20.6483	21.3768	21.315	19.9496	22.6843	21.9607	23.1434	22.6204	0.999956	0.0267072	55.112	13.145	55.112	1.0014	188400000	54	2.29417	0.415	-1.7798	-3.46481	54	H9JML7	RFLSFTVDPKYLFSTSEKYKRKECLC	Y(1)LFS(1)T(1)S(1)EKYKR	555	Uncharacterized protein OS	UID4136	4	S	884
448	20.6483	21.3768	21.315	19.9496	22.6843	21.9607	23.1434	22.6204	0.99996	0.0267072	55.112	13.145	55.112	1.0014	188400000	53	2.29417	0.83	-1.7798	-3.46481	53	H9JML7	PRFLSFTVDPKYLFSTSEKYKRKECL	Y(1)LFS(1)T(1)S(1)EKYKR	1305	Uncharacterized protein OS	UID4886	4	T	884
449	20.6483	21.3768	21.315	19.9496	22.6843	21.9607	23.1434	22.6204	0.999983	0.0267072	55.112	13.145	55.112	1.0014	188400000	49	2.29417	0.553333	-1.7798	-3.46481	49	H9JML7	NVVDPRFLSFTVDPKYLFSTSEKYKR	Y(1)LFS(1)T(1)S(1)EKYKR	1701	Uncharacterized protein OS	UID5282	4	Y	884
450	NaN	NaN	NaN	NaN	NaN	NaN	NaN	NaN	1	0.0359143	68.383	13.843	68.383	-2.3497	5647500	2	NaN	1	0	NaN	2	D3JSV9	MSKIIKFDLRELK	MS(1)KIIKFDLR	33	RPA43_N protein OS	UID34	1	S	91
451	NaN	NaN	NaN	NaN	NaN	NaN	NaN	NaN	1	0.0343372	76.927	13.852	76.927	-3.3728	2862500	205	NaN	1	0	NaN	205	H9IV67	RIDDCISSFQWFRGASPAMKLELEK	GAS(1)PAMKLELEK	87	MFS domain-containing protein OS	UID88	1	S	182
452	NaN	NaN	NaN	NaN	NaN	NaN	NaN	NaN	1	0.0319031	55.401	29.289	45.433	-2.7282	490960	68	NaN	1	0	NaN	68	H9IZC4	ARPCAVIGPDRVIPPSQNDCYLKKA	VIPPS(1)QNDCY(1)LKK	168	Uncharacterized protein OS	UID1959		S	289
453	NaN	NaN	NaN	NaN	NaN	NaN	NaN	NaN	1	0.0365634	49.368	29.529	49.368	-2.1805	17476000	9	NaN	1	0	NaN	9	H9JJD6	MAAALAHDSPKYRGSLIC	MAAALAHDS(1)PKY(1)R	493	Phorbol-ester/DAG-type domain-containing protei	UID2284	2	S	804
454	NaN	NaN	NaN	NaN	NaN	NaN	NaN	NaN	1	0.0160861	101.97	38.691	101.97	2.2891	6354100	535	NaN	1	0	NaN	535	H9JKF4	SLDRNPTPQQRQLLESKPNISINKSL	QLLES(1)KPNIS(1)INK	512	PHD-type domain-containing protein OS	UID2303	2	S	828
455	NaN	NaN	NaN	NaN	NaN	NaN	NaN	NaN	1	0.0160861	101.97	38.691	101.97	2.2891	6354100	540	NaN	1	0	NaN	540	H9JKF4	PTPQQRQLLESKPNISINKSLQTPFP	QLLES(1)KPNIS(1)INK	513	PHD-type domain-containing protein OS	UID2304	2	S	828
456	NaN	NaN	NaN	NaN	NaN	NaN	NaN	NaN	0.988521	0.0199741	56.936	23.744	56.936	3.5566	3034700	6	NaN	1	0	NaN	6	H9JUC7	MFMPFSKTIQALGQS	MFMPFS(0.989)KT(0.954)IQALGQS(	703	Uncharacterized protein OS	UID2494	2	S	1069
457	NaN	NaN	NaN	NaN	NaN	NaN	NaN	NaN	0.999998	0.0209432	71.806	21.584	71.806	4.159	2164900	61	NaN	1	0	NaN	61	H9JUW9	SELLDLLKYVAFMPFSDITRLSTLIKD	TSELLDLLKYVAFMPFS(1)DIT(1)R	713	Uncharacterized protein OS	UID2504	2	S	1078
458	NaN	NaN	NaN	NaN	NaN	NaN	NaN	NaN	0.953923	0.0199741	56.936	23.744	56.936	3.5566	3034700	8	NaN	1	0	NaN	8	H9JUC7	MFMPFSKTIQALGQSVT	MFMPFS(0.989)KT(0.954)IQALGQS(	1401	Uncharacterized protein OS	UID3192	2	T	1069
459	NaN	NaN	NaN	NaN	NaN	NaN	NaN	NaN	0.999998	0.0209432	71.806	21.584	71.806	4.159	2164900	64	NaN	1	0	NaN	64	H9JUW9	LDLLKYVAFMPFSDITRLSTLIKDKF	TSELLDLLKYVAFMPFS(1)DIT(1)R	1403	Uncharacterized protein OS	UID3194	2	T	1078
460	NaN	NaN	NaN	NaN	NaN	NaN	NaN	NaN	1	0.0319031	55.401	29.289	45.433	-2.7282	490960	73	NaN	1	0	NaN	73	H9IZC4	VIGPDRVIPPSQNDCYLKKARVEPW	VIPPS(1)QNDCY(1)LKK	1553	Uncharacterized protein OS	UID3344		Y	289
461	NaN	NaN	NaN	NaN	NaN	NaN	NaN	NaN	1	0.0365634	49.368	29.529	49.368	-2.1805	17476000	12	NaN	1	0	NaN	12	H9JJD6	MAAALAHDSPKYRGSLICCNP	MAAALAHDS(1)PKY(1)R	1677	Phorbol-ester/DAG-type domain-containing protei	UID3468	2	Y	804
462	NaN	NaN	NaN	NaN	NaN	NaN	NaN	NaN	0.994792	0.0374454	51.135	20.137	51.135	4.0571	515820	244	NaN	1	0	NaN	244	H9IUI5	LKARQLRQNFKQTNKSLTERSSQTL	S(0.995)LT(0.995)ERS(0.014)S(0.963	74	Uncharacterized protein OS	UID3655		S	167
463	NaN	NaN	NaN	NaN	NaN	NaN	NaN	NaN	0.963014	0.0374454	51.135	20.137	51.135	4.0571	515820	250	NaN	1	0	NaN	250	H9IUI5	RQNFKQTNKSLTERSSQTLNSRERA	S(0.995)LT(0.995)ERS(0.014)S(0.963	75	Uncharacterized protein OS	UID3656		S	167
464	NaN	NaN	NaN	NaN	NaN	NaN	NaN	NaN	0.96406	0.0376979	48.423	16.92	48.423	1.4241	2170700	281	NaN	1	0	NaN	281	H9IXX2	HFETIPKQITLYIQKSKDGYITPVKDV	QIT(0.561)LY(0.475)IQKS(0.964)KDG	135	Uncharacterized protein OS	UID3716		S	258
465	NaN	NaN	NaN	NaN	NaN	NaN	NaN	NaN	0.925956	0.0330723	45.267	23.738	45.267	0.84347	125980000	1345	NaN	1	0	NaN	1345	H9JDB6	AYFWVANDFADEAVASTPSDVSSK	NET(0.056)KAY(0.955)FWVANDFADE	403	Uncharacterized protein OS	UID3984	4	S	663
466	NaN	NaN	NaN	NaN	NaN	NaN	NaN	NaN	0.998792	0.0129786	61.167	16.728	49.973	-0.70629	1959300	145	NaN	1	0	NaN	145	H9JKW5	ILAVATVEGKLKMFVSRLIKFNEFAL	T(0.664)T(0.664)LDT(0.664)ILAVAT(	523	Uncharacterized protein OS	UID4104	3	S	839
467	NaN	NaN	NaN	NaN	NaN	NaN	NaN	NaN	1	0.0317814	58.596	7.9892	58.596	-0.39278	837240	137	NaN	1	0	NaN	137	H9JNY1	HVSLADGTARVAFLPSTVTPREIADI	VAFLPS(1)T(1)VT(1)PR	583	Uncharacterized protein OS	UID4164	3	S	915
468	NaN	NaN	NaN	NaN	NaN	NaN	NaN	NaN	0.999995	0.0383285	63.091	22.595	63.091	-3.5597	3038000	291	NaN	1	0	NaN	291	H9JX46	VSALYEKRFNKSIITSIIYPKNKNKVP	FNKSIIT(1)S(1)IIY(1)PK	752	Calpain catalytic domain-containing protein OS	UID4333	3	S	1126
469	NaN	NaN	NaN	NaN	NaN	NaN	NaN	NaN	1	0.0140218	84.31	10.679	84.31	-3.0036	53034000	10	NaN	1	0	NaN	10	H9JY16	MLDETVVPESESDVMVRR	MLDET(1)VVPES(1)ES(1)DVMVRR	763	Uncharacterized protein OS	UID4344	3	S	1152
470	NaN	NaN	NaN	NaN	NaN	NaN	NaN	NaN	1	0.0140218	84.31	10.679	84.31	-3.0036	53034000	12	NaN	1	0	NaN	12	H9JY16	MLDETVVPESESDVMVRRAR	MLDET(1)VVPES(1)ES(1)DVMVRR	764	Uncharacterized protein OS	UID4345	3	S	1152
471	NaN	NaN	NaN	NaN	NaN	NaN	NaN	NaN	0.936726	0.0331453	52.72	18.528	52.72	-4.394	309050	421	NaN	1	0	NaN	421	H9JYA1	LEKIYLQTFRCNSPKSFRSSTSSVDS	S(0.937)FRS(0.932)S(0.932)T(0.209)	769	Tcp10_C domain-containing protein OS	UID4350		S	1157
472	NaN	NaN	NaN	NaN	NaN	NaN	NaN	NaN	0.932183	0.0331453	52.72	18.528	52.72	-4.394	309050	424	NaN	1	0	NaN	424	H9JYA1	IYLQTFRCNSPKSFRSSTSSVDSNSN	S(0.937)FRS(0.932)S(0.932)T(0.209)	770	Tcp10_C domain-containing protein OS	UID4351		S	1157
473	NaN	NaN	NaN	NaN	NaN	NaN	NaN	NaN	0.93221	0.0331453	52.72	18.528	52.72	-4.394	309050	425	NaN	1	0	NaN	425	H9JYA1	YLQTFRCNSPKSFRSSTSSVDSNSNI	S(0.937)FRS(0.932)S(0.932)T(0.209)	771	Tcp10_C domain-containing protein OS	UID4352		S	1157
474	NaN	NaN	NaN	NaN	NaN	NaN	NaN	NaN	0.766701	0.0331453	52.72	18.528	52.72	-4.394	309050	427	NaN	1	0	NaN	427	H9JYA1	QTFRCNSPKSFRSSTSSVDSNSNIQ	S(0.937)FRS(0.932)S(0.932)T(0.209)	772	Tcp10_C domain-containing protein OS	UID4353		S	1157
475	NaN	NaN	NaN	NaN	NaN	NaN	NaN	NaN	0.99525	0.0374454	51.135	20.137	51.135	4.0571	515820	246	NaN	1	0	NaN	246	H9IUI5	ARQLRQNFKQTNKSLTERSSQTLNS	S(0.995)LT(0.995)ERS(0.014)S(0.963	892	Uncharacterized protein OS	UID4473		T	167
476	NaN	NaN	NaN	NaN	NaN	NaN	NaN	NaN	0.937114	0.0374454	51.135	20.137	51.135	4.0571	515820	252	NaN	1	0	NaN	252	H9IUI5	NFKQTNKSLTERSSQTLNSRERAQ	S(0.995)LT(0.995)ERS(0.014)S(0.963	893	Uncharacterized protein OS	UID4474		T	167
477	NaN	NaN	NaN	NaN	NaN	NaN	NaN	NaN	0.999864	0.0376979	48.423	16.92	48.423	1.4241	2170700	287	NaN	1	0	NaN	287	H9IXX2	KQITLYIQKSKDGYITPVKDVTIVDLS	QIT(0.561)LY(0.475)IQKS(0.964)KDG	935	Uncharacterized protein OS	UID4516		T	258
478	NaN	NaN	NaN	NaN	NaN	NaN	NaN	NaN	0.876451	0.0330723	45.267	23.738	45.267	0.84347	125980000	1346	NaN	1	0	NaN	1346	H9JDB6	YFWVANDFADEAVASTPSDVSSKS	NET(0.056)KAY(0.955)FWVANDFADE	1176	Uncharacterized protein OS	UID4757	4	T	663
479	NaN	NaN	NaN	NaN	NaN	NaN	NaN	NaN	1	0.0317814	58.596	7.9892	58.596	-0.39278	837240	138	NaN	1	0	NaN	138	H9JNY1	VSLADGTARVAFLPSTVTPREIADLI	VAFLPS(1)T(1)VT(1)PR	1319	Uncharacterized protein OS	UID4900	3	T	915
480	NaN	NaN	NaN	NaN	NaN	NaN	NaN	NaN	1	0.0317814	58.596	7.9892	58.596	-0.39278	837240	140	NaN	1	0	NaN	140	H9JNY1	LADGTARVAFLPSTVTPREIADLYEI	VAFLPS(1)T(1)VT(1)PR	1320	Uncharacterized protein OS	UID4901	3	T	915
481	NaN	NaN	NaN	NaN	NaN	NaN	NaN	NaN	0.993695	0.0342231	41.911	14.357	41.911	-0.071278	1302700	61	NaN	1	0	NaN	61	H9JQ12	MKQLAKDPNMDFSKRTAVYVGGY	T(0.994)AVY(0.994)VGGY(0.889)MD	1335	Lipase domain-containing protein OS	UID4916		T	950
482	NaN	NaN	NaN	NaN	NaN	NaN	NaN	NaN	0.999954	0.0383285	63.091	22.595	63.091	-3.5597	3038000	290	NaN	1	0	NaN	290	H9JX46	AVSALYEKRFNKSIITSIIYPKNKNKV	FNKSIIT(1)S(1)IIY(1)PK	1426	Calpain catalytic domain-containing protein OS	UID5007	3	T	1126
483	NaN	NaN	NaN	NaN	NaN	NaN	NaN	NaN	1	0.0140218	84.31	10.679	84.31	-3.0036	53034000	5	NaN	1	0	NaN	5	H9JY16	MLDETVVPESESDV	MLDET(1)VVPES(1)ES(1)DVMVRR	1437	Uncharacterized protein OS	UID5018	3	T	1152
484	NaN	NaN	NaN	NaN	NaN	NaN	NaN	NaN	0.999908	0.0376979	48.423	16.92	48.423	1.4241	2170700	285	NaN	1	0	NaN	285	H9IXX2	IPKQITLYIQKSKDGYITPVKDVTIVD	QIT(0.561)LY(0.475)IQKS(0.964)KDG	1541	Uncharacterized protein OS	UID5122		Y	258
485	NaN	NaN	NaN	NaN	NaN	NaN	NaN	NaN	0.95531	0.0330723	45.267	23.738	45.267	0.84347	125980000	1331	NaN	1	0	NaN	1331	H9JDB6	TSEMEISPMKNETKAYFWVANDFA	NET(0.056)KAY(0.955)FWVANDFADE	1645	Uncharacterized protein OS	UID5226	4	Y	663
486	NaN	NaN	NaN	NaN	NaN	NaN	NaN	NaN	0.993656	0.0342231	41.911	14.357	41.911	-0.071278	1302700	64	NaN	1	0	NaN	64	H9JQ12	LAKDPNMDFSKRTAVYVGGYMDS	T(0.994)AVY(0.994)VGGY(0.889)MD	1721	Lipase domain-containing protein OS	UID5302		Y	950
487	NaN	NaN	NaN	NaN	NaN	NaN	NaN	NaN	0.88881	0.0342231	41.911	14.357	41.911	-0.071278	1302700	68	NaN	1	0	NaN	68	H9JQ12	PNMDFSKRTAVYVGGYMDSPSYP	T(0.994)AVY(0.994)VGGY(0.889)MD	1722	Lipase domain-containing protein OS	UID5303		Y	950
488	NaN	NaN	NaN	NaN	NaN	NaN	NaN	NaN	0.999995	0.0383285	63.091	22.595	63.091	-3.5597	3038000	294	NaN	1	0	NaN	294	H9JX46	LYEKRFNKSIITSIIYPKNKNKVPVYN	FNKSIIT(1)S(1)IIY(1)PK	1776	Calpain catalytic domain-containing protein OS	UID5357	3	Y	1126
